# Comprehensive transcriptome analysis reveals novel genes involved in cardiac glycoside biosynthesis and mlncRNAs associated with secondary metabolism and stress response in *Digitalis purpurea*

**DOI:** 10.1186/1471-2164-13-15

**Published:** 2012-01-10

**Authors:** Bin Wu, Ying Li, Haixia Yan, Yimian Ma, Hongmei Luo, Lichai Yuan, Shilin Chen, Shanfa Lu

**Affiliations:** 1 Institute of Medicinal Plant Development, Chinese Academy of Medical Sciences & Peking Union Medical College, No.151, Malianwa North Road, Haidian District, Beijing 100193, China

## Abstract

**Abstract:**

**Background**

*Digitalis purpurea *is an important ornamental and medicinal plant. There is considerable interest in exploring its transcriptome.

**Results**

Through high-throughput 454 sequencing and subsequent assembly, we obtained 23532 genes, of which 15626 encode conserved proteins. We determined 140 unigenes to be candidates involved in cardiac glycoside biosynthesis. It could be grouped into 30 families, of which 29 were identified for the first time in *D. purpurea*. We identified 2660 mRNA-like npcRNA (mlncRNA) candidates, an emerging class of regulators, using a computational mlncRNA identification pipeline and 13 microRNA-producing unigenes based on sequence conservation and hairpin structure-forming capability. Twenty five protein-coding unigenes were predicted to be targets of these microRNAs. Among the mlncRNA candidates, only 320 could be grouped into 140 families with at least two members in a family. The majority of *D. purpurea *mlncRNAs were species-specific and many of them showed tissue-specific expression and responded to cold and dehydration stresses. We identified 417 protein-coding genes with regions significantly homologous or complementary to 375 mlncRNAs. It includes five genes involved in secondary metabolism. A positive correlation was found in gene expression between protein-coding genes and the homologous mlncRNAs in response to cold and dehydration stresses, while the correlation was negative when protein-coding genes and mlncRNAs were complementary to each other.

**Conclusions:**

Through comprehensive transcriptome analysis, we not only identified 29 novel gene families potentially involved in the biosynthesis of cardiac glycosides but also characterized a large number of mlncRNAs. Our results suggest the importance of mlncRNAs in secondary metabolism and stress response in *D. purpurea*.

## Background

*Digitalis purpurea *L. (common foxglove, purple foxglove or lady's glove) is an herbaceous biennial flowering plant species. It is native to Europe and has been widely introduced into other parts of the world. Now, this plant becomes naturalized in many countries, such as China, Canada, USA, and New Zealand [1]. *D. purpurea *is known for its campanulate flowers produced on a tall spike. The flower color varies from purple to pink, yellow or white. Because of the showy flowers, *D. purpurea *has an ornamental value and is cultivated world widely. However, this plant is highly poisonous to humans and may be fatal if ingested, so it is suggested to be banished from the table [2,3]. Although *D. purpurea *is toxic, it has a high medical value in the therapy of congestive heart failure, particularly arrhythmia for over 200 years [1]. Many patients benefited from the use of this plant and its extracts. The main active compounds of *D. purpurea *are cardiac glycosides (cardenolides), which are a group of cardio-active agents with the ability to inhibit Na^+^/K^+^-ATPase and have been proved to be the most effective drugs to treat heart failure during the past two centuries [1,4,5]. *D. purpurea *is also a potential source of anti-tumor drugs because of its high cytotoxicity against human cancer cell lines [6]. The discovery of cardio-active agents in *D. purpurea *by the 18th century Scottish physician William Withering is thought to help launching the development of modern pharmacology and the pharmaceutical industry, further suggesting the importance of *D. purpurea *to medicine [7].

*D. purpurea *tolerates many environmental stresses, such as cold, drought, heat, flooding, full sun and poor soil, although it prefers rich and well-drained acidic soils and half shade and moist environments. This plant can survive in most temperate climates of the world, including the cold temperate in Alaska of the United States and south-western Norway and the hot and drought conditions in some areas of Africa [1,8]. Being a biennial plant, *D. purpurea *is able to withstand the winter cold and the summer drought [9]. Significant damage to *D. purpurea *leaves were happened only at -12°C or below and the threshold for buds and roots was as low as -15°C and -18°C, respectively [9]. Thus, *D. purpurea *seems to be an ideal species for studying plant responses to drought and cold stresses.

Although *D. purpurea *exhibits significance in ornament, medicine and biological study, little has been done about genes involved in the growth and development of this plant species. No EST sequences are available and only 77 nucleotide records are found for *D. purpurea *in the NCBI nucleotide database http://www.ncbi.nlm.nih.gov/nucleotide/. The major efforts to study the molecular aspects of *D. purpurea *were focused on the purification and characterization of malonyl-coenzyme A: 21-hydroxypregnane 21-*O*-malonyltransferase (Dp21MaT) and the identification of genes encoding progesterone 5β-reductase (5β-POR) and Δ^5^-3β-hydroxysteroid dehydrogenase (3βHSD), which are involved in the biosynthesis of cardiac glycosides [5,10-12].

Non-protein-coding RNAs (npcRNAs), also known as noncoding RNAs (ncRNAs), are transcripts that have neither experimental nor evolutionary evidence for an open reading frame encoding a functional protein [13]. They are produced in both eukaryotes and prokaryotes and represent the vast majority of all transcripts in a cell as demonstrated by genomic tiling arrays, whole transcriptome analysis, reverse transcription-RCR (RT-PCR) and computational prediction [14-26]. In human, more than 90% of the total genome sequence is likely to be transcribed, but only about 1.5% of the genome codes for proteins, suggesting the abundance of human npcRNA species [15,27]. The high percentage of transcription in non-protein-coding regions was also reported for other eukaryotes, such as mouse, yeast and *Drosophila melanogaster *[13,28-32]. Now, npcRNA is a research hotspot of RNA biology and the number of identified npcRNAs is increasing rapidly.

Results from gene expression profiling and functional analysis strongly suggested that most of the npcRNAs could be biologically meaningful rather than merely transcriptional "noise" [13,32]. Based on the expression profiles and predicted functions, npcRNAs can be classified into two groups, including housekeeping npcRNAs and regulatory npcRNAs [13]. Housekeeping npcRNAs are usually expressed constitutively and include rRNAs, tRNAs, snRNAs and snoRNAs, whereas regulatory npcRNAs are differentially expressed and developmentally regulated and can be subdivided into short regulatory npcRNAs (or small RNAs, sRNAs, < 40 nucleotides), such as microRNAs (miRNAs) and small interfering RNAs (siRNAs), and large regulatory npcRNAs (or long npcRNAs, lncRNAs, > 40 nucleotides) that may or may not be polyadenylated. A subset of lncRNAs, which can undergo splicing and are capped and polyadenylated as conventional mRNAs, are called mRNA-like npcRNAs (mlncRNAs) [33].

Recently, sRNAs, particularly miRNAs, have been intensely studied [34]. The number of identified miRNAs has reached to 16772 according to the most recent release of miRBase (release 17, http://www.mirbase.org/). It includes 3362 miRNAs from plants, 237 from viruses, and more than 13,000 from animals. In plants, miRNAs play crucial roles in organ development and defense responses by targeting other RNA molecules for cleavage, or, in a few cases, for translational repression [35-38]. lncRNAs are much less studied as compared with mRNAs and sRNAs and represent a major unexplored transcript species [13]. So far, only a small number of lncRNAs have been functionally characterized [32]. The known roles of lncRNAs in animals include transcriptional regulation, epigenetic gene regulation, and disease response [13]. Studies of plant lncRNAs were mainly concentrated on systematic searches of mlncRNAs in *Arabidopsis thaliana*, wheat, and *Medicago truncatula *[39-46]. Only few were identified in other plant species [47-53]. Several of the identified plant mlncRNAs, including *Arabidopsis *AtIPS1/At4 and COLDAIR, *Cucumis sativus *CsM10, *Zea mays *Zm401 and *M. truncatula *enod40, were functionally characterized. The results suggested important regulatory roles of plant mlncRNAs in phosphate deprivation response, vernalization response, pollen development, gender-biased expression, and nodulation [47,49-57]. Functions of the other plant mlncRNAs are largely unknown.

In this study, we performed a comprehensive analysis of *D. purpurea *transcriptome that had never been explored. We obtained a high-quality unigene set and determined conserved and non-conserved protein-coding genes, including candidates for about 75% of the total cardiac glycoside biosynthesis-associated gene families in *D. purpurea*. We identified a large number of mlncRNA candidates and 13 microRNA-producing unigenes and predicted 25 microRNA targets. Using a comprehensive approach, we revealed some important characteristics of mlncRNAs and took efforts to identify mlncRNA-regulated protein-coding genes. These results enhance our knowledge of cardiac glycoside biosynthesis and provide useful information for understanding the function of mlncRNAs.

## Results

### High-throughput sequencing and assembly of *D. purpurea *transcriptome

**Figure 1:**
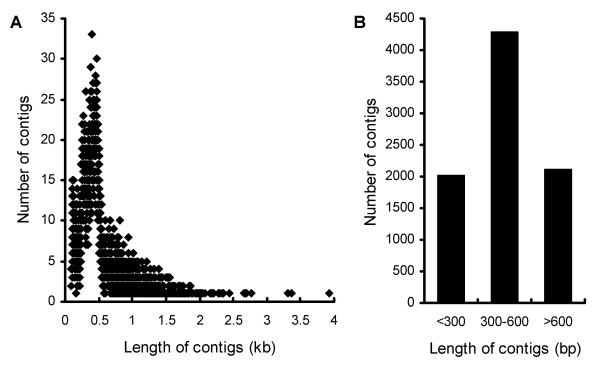
**Size distribution of contigs assembled from 454 pyrosequencing data**. The number of contigs with same length (A) and the number of contigs with length less than 300 bp, 300-600 bp and larger than 600 bp (B) are shown.

In order to explore the transcriptome of *D. purpurea*, we constructed a cDNA library of the leaves of one-year-old plants and sequenced it using the 454 ultra-high-throughput pyrosequencing platform, the Genome Sequencer FLX System. We obtained a total of 66103 high-quality reads [GenBank SRA database series identifier SRX084241] with an average length of 402 bases. Pre-processing and *de novo *assembly using the GS *De Novo *Assembler Software clustered 51056 reads (77.2% of the total high-quality reads) into 8413 contigs [GenBank TSA database accession nos. JO460003-JO467642] and left 15119 singletons, yielding a total of 23532 unigenes. Original reads of contigs ranged from 2 to 268 with an average 6, and lengths ranged from 94 bp to 3927 bp with an average 519 bp. The size distribution showed that most of contigs ranged from 300 bp to 600 bp and the percentage of contigs no less than 300 bp was 76% (Figure 1). These data suggested that most of the pyrosequencing data had been successfully assembled into relatively long contigs.

### Functional annotation and gene ontology analysis

Functional annotation of unigenes was carried out by sequence similarity searches against the NCBI non-redundant (Nr) protein database using the Basic Local Alignment Search Tool (BLAST) [58]. We applied a generous e-value cutoff of 10^-5 ^to the BLAST homolog recognition. 15626 (66.4%) of total unigenes were annotated according to the proposed function of the best match for each unigene in the Nr protein database. These annotated unigenes were protein-coding genes conserved in other species. The remaining 7906 unigenes (33.6%), which did not show significant similarity to any sequences in the Nr protein database, could be npcRNAs, UTR regions of known protein-coding genes, or novel protein-coding transcripts.

**Figure 2:**
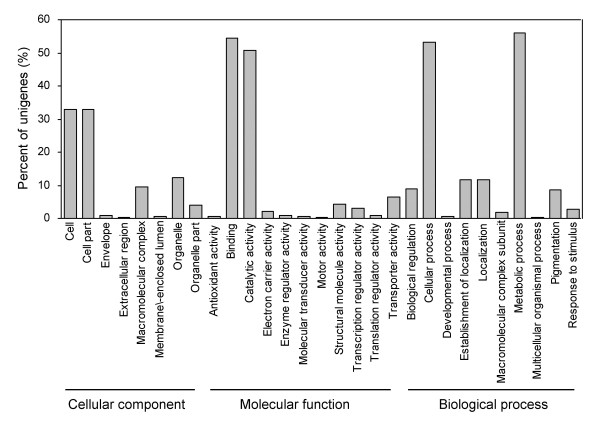
**Gene ontology analysis of *D. purpurea *unigenes**. Percent of unigenes assigned to 29 subcategories of the cellular location, molecular function and biological process categories is shown.

The *D. purpurea *unigenes were further analyzed and categorized using the Gene Ontology (GO), which had been widely used to standardize the representation of gene and gene product attributes across species and to describe gene products in terms of their associated biological processes, cellular locations and molecular functions in a species-independent manner [59]. GO analysis annotated a total of 6462 unigenes and assigned one or more GO terms to each of them (Figure 2). The category of molecular function, which included 5750 unigenes, was the largest, followed by the biological process category (4607 unigenes) and the cellular location category (2144 unigenes). GO assignments of 29 subcategories of the three categories were shown in Figure 2. Metabolic process, protein binding, cellular process and catalytic activity were the largest four subcategories.

### Identification of protein-coding genes involved in cardiac glycoside biosynthesis

**Figure 3:**
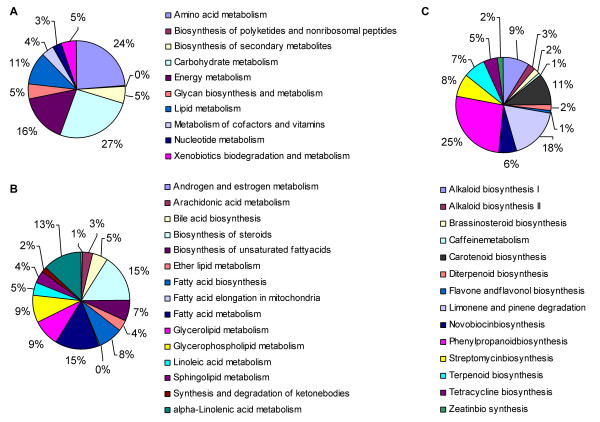
**Percentages of *D. purpurea *unigenes in 10 subcategories of the metabolic pathway category (A), 15 sub-sub-categories of the lipid metabolism subcategory (B), and 14 sub-sub-categories of the biosynthesis of secondary metabolites subcategory (C)**.

*D. purpurea *is a useful medicinal plant species in heart failure treatment [1]. Isolation and characterization of genes involved in the biosynthesis of secondary metabolites, such as cardiac glycosides, is particularly valuable. However, little research had been done on this area. With the aim of identifying cardiac glycoside biosynthesis-associated genes on a transcriptome-wide scale, we first analyzed the 23532 *D. purpurea *unigenes by sequence similarity searches against the Kyoto Encyclopedia of Genes and Genomes database (KEGG) and then searched for unigenes associated with metabolic pathways [60]. Using an E-value cutoff of 10^-5^, we computationally annotated 12921 unigenes (54.9%), of which 1400 were assigned to the KEGG metabolic pathway category, suggesting a number of unigenes associated with metabolic pathways of *D. purpurea *were obtained. Within the metabolic pathway category, unigenes were separated into 10 subcategories (Figure 3A).

Further analysis of unigene assignment in the subcategories showed that 797 of the 1400 unigenes each were classified to a subcategory, while the other 603 each were assigned to two or more subcategories. Among the 10 subcategories of metabolic pathway, carbohydrate metabolism was assigned the largest amount of unigenes (Figure 3A), followed by amino acid metabolism, energy metabolism, lipid metabolism, biosynthesis of secondary metabolites, and the others (Figure 3A). The biosynthesis of steroids and terpenoids were well-represented by unigenes in the subcategories of lipid metabolism and biosynthesis of secondary metabolites, allowing the effective identification of cardiac glycoside biosynthesis-related genes in *D. purpurea *(Figure 3B, C).

*Digitali*s cardiac glycosides are a class of plant secondary metabolites consisting of a steroid nucleus and a sugar side chain varied in length [61]. The putative biosynthetic pathway of plant cardiac glycosides roughly comprises terpenoid backbone biosynthesis, steroid biosynthesis and cardenolide biosynthesis three stages, involving about 40 gene families, of which only a few had been characterized in *D. purpurea*. Examination of unigenes assigned to the subcategories of lipid metabolism and biosynthesis of secondary metabolites allowed us to identify a total of 140 unigenes that could be associated with the biosynthesis of terpenoid backbone, steroids and cardenolides (see Additional file 1). These unigenes were grouped into 30 gene families, representing about 75% of the total cardiac glycoside biosynthesis-associated gene families.

Among the 140 unigenes, 24 assembled from 103 original 454 reads were involved in the biosynthesis of terpenoid backbone, and represented 13 of the 16 terpenoid backbone biosynthesis-associated gene families (see Additional file 1). Twenty unigenes assembled from 118 original 454 reads were identified to represent 12 of the 13 putative steroid biosynthesis-associated gene families, suggesting genes involved in the steroid biosynthesis were also well-represented in the unigene set (see Additional file 1).

Different from that involved in the biosynthesis of terpenoid backbone and steroids, genes associated with cardenolide biosynthesis were not well-represented. The putative pathway of cardenolide biosynthesis involved at least 11 gene families, of which only 5 were represented by unigenes. It includes those encoding Δ^5^-3β-hydroxysteroid dehydrogenases (3β-HSD), progesterone 5β-reductases (5β-POR), mono-oxygenases, cardenolide 16'-O-glucohydrolases (CGH), and glycosyltransferases (GT) (see Additional file 1). *3β-HSD *and *5β-POR *and mono-oxygenase genes are involved in the initial steps of cardenolide biosynthesis toward cardenolide genin formation, *GT *genes are responsible for addition of sugars to the genins, whereas CGH is involved in the degradation of primary cardiac glycosides. The gene families of mono-oxygenases and GTs are usually super-large in plants and play crucial roles in various cellular and metabolic processes. Consistently, in the 454 EST database, mono-oxygenases were represented by 36 unigenes assembled from 100 original 454 reads, while GTs were represented by 56 unigenes from 133 reads. The exact mono-oxygenase and *GT *genes involved in cardenolide biosynthesis remained to elucidate. It is worthy to note that the unigene encoding CGH was listed among the 25 most abundant unigenes (see Additional file 2). Low representativeness of unigenes for cardenolide biosynthesis-associated gene families in our 454 EST database and high expression of the gene involved in primary cardiac glycoside hydrolysis were consistent with overall low cardiac glycoside content in *D. purpurea *leaves, although relative to other organs leaves have the highest content of cardiac glycosides [62]. These data greatly enhanced our knowledge of *Digitalis *cardiac glycoside biosynthesis and provided candidate genes for improving the production of active medicinal compounds in *D. purpurea *through genetic engineering.

### Transcriptome-wide identification of *D. purpurea *mlncRNA candidates

**Figure 4:**
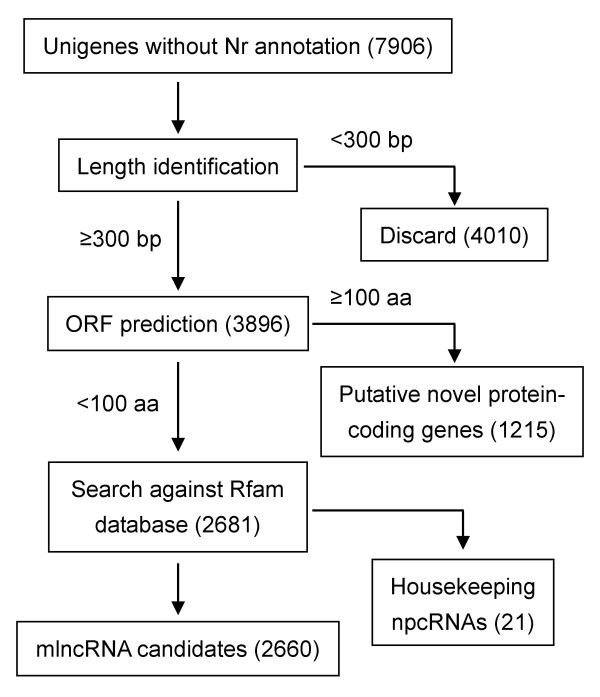
**Schematic outline of novel protein-coding genes and mlncRNA candidates**. The number of unigenes is shown in parentheses. ORF, open reading frame.

Although npcRNA had been found in various organisms, systematically searches of plant mlncRNAs were carried out only in *A. thaliana*, wheat and *M. truncatula *[39-46]. In *D. purpurea*, among the 23532 unigenes analyzed, 7906 (33.6%) could be novel protein-coding transcripts or npcRNAs because they did not show significant similarity to any sequences in the Nr protein database. Among the 7906 unigenes, 4010, which were not further analyzed, were less than 300 bp in length. The other 3896 with sizes at least 300 bp were subjected to detailed characterization using the pipeline summarized in Figure 4.

**Figure 5:**
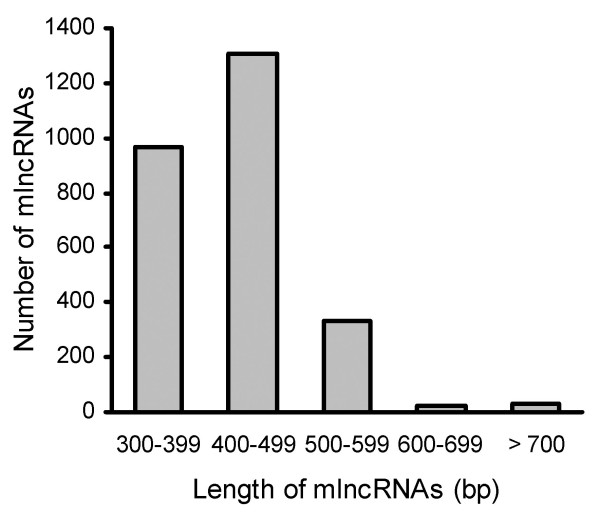
**Size distribution of mlncRNA candidates**.

The pipeline we used is similar to those reported previously for systematic screens of mlncRNAs from the EST databases of *Arabidopsis *[39,40,42], *Medicago *[45], *Sus scrofa *[63], *Drosophila *[64], and human [65]. A cutoff of 100 amino acids was applied in this study to distinguish npcRNAs from protein-coding transcripts as suggested by Rymarquis et al (2008). The results showed that 1215 of the 3896 unigenes with sizes at least 300 bp had the potential to encode proteins, while the other 2681 unigenes were npcRNA candidates. It includes 2660 mlncRNAs and 21 housekeeping npcRNAs (a tRNA precursor and 20 snoRNA precursors) (see Additional file 3). Low number of non-polyadenylated tRNA and snoRNAs are consistent with our experimental strategy to select polyadenylated RNAs for library construction. Being consistent with the size distribution of contigs, most of the identified mlncRNA candidates have sizes ranged between 300 bp and 600 bp (Figure 5). The others include 22 with 600-699 bp in length and 30 over 700 bp (Table 1). The long ones, such as those with sizes over 600 bp, are very likely to be authentic mlncRNAs, whereas we cannot rule out the possibility that some of the short ones are UTRs of protein coding genes or contain partial ORFs and UTRs of protein coding genes. Further experimental cloning of full-length transcripts may help verify those short mlncRNA candidates. These results suggest the existence of a large number of mlncRNAs in *D. purpurea*.

**Table 1:** *D. purpurea *mlncRNA candidates with sizes over 700 bp

npcRNA name	Unigene ID	Length (bp)	number of 454 reads
mlncR1	JO460015	3923	102
mlncR2	JO460538	2159	33
mlncR3	JO467387	2028	41
mlncR4	JO460228	1264	13
mlncR5	JO460314	1252	21
mlncR6	JO460006	1221	6
mlncR7	JO460072	1163	11
mlncR8	JO463019	1112	8
mlncR9	JO460086	1095	45
mlncR10	JO460027	1081	8
mlncR11	JO463036	1022	10
mlncR12	JO464553	967	5
mlncR13	JO461863	919	6
mlncR14	JO460868	884	4
mlncR15	JO460756	877	17
mlncR16	JO461597	811	10
mlncR17	JO464502	801	6
mlncR18	JO460757	800	15
mlncR19	JO461384	784	14
mlncR20	JO462548	774	5
mlncR21	JO460109	770	23
mlncR22	JO463958	767	8
mlncR23	JO460543	753	4
mlncR24	JO460800	748	7
mlncR25	JO463091	734	10
mlncR26	JO467391	731	15
mlncR27	JO463416	727	5
mlncR28	JO460815	709	5
mlncR29	JO460003	708	4
mlncR30	JO462174	706	9

### Identification of 13 *D. purpurea *microRNAs

**Figure 6:**
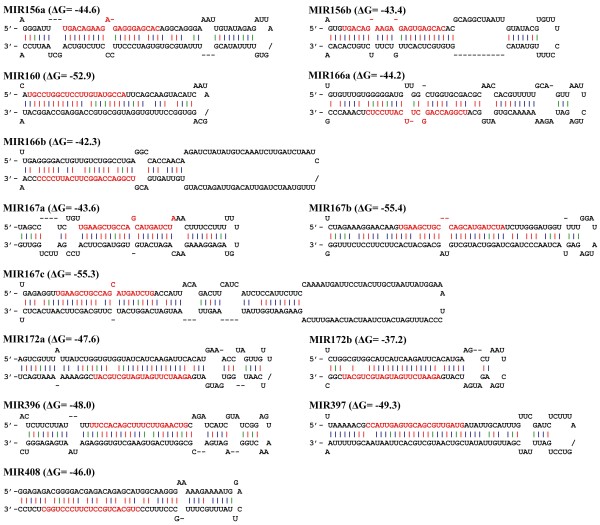
**Predicted hairpin structures of *D. purpurea *miRNA precursors**. Mature miRNA sequences are indicated in red. The red, green and blue vertical lines indicate G:C, G:U and A:U pairing, respectively.

microRNAs (miRNAs) are a class of small noncoding RNAs derived from long primary miRNAs and play crucial roles in organ development and defense responses [35-38]. Many miRNAs are deeply conserved across species boundaries. It allows us to identify *D. purpurea *miRNAs by computational search of our unigene set based on the conservation of miRNA sequences and the secondary structure of unigenes [66]. We, therefore, searched the unigene set for sequences similar to the miRNAs included in the most recent release of miRBase (release 17, http://www.mirbase.org/) [67] using the BLASTN program [58], and then performed mfold analysis of hairpin structures for those unigenes with regions no more than three mismatches to known miRNAs [68]. As a result, we identified a total of 13 miRNA-producing unigenes, which counted for 0.06% of total *D. purpurea *unigenes (Table 2). Except FXAT9O005F5R10, which is 209 bp in length, the other 12 miRNA-producing unigenes are mlncRNAs with sizes at least 300 bp. It includes JO460086 (mlncR9), one of the mlncRNAs with sizes over 700 bp (Table 1). Based on the predicted miRNA sequences, these unigenes were classified into eight families, of which four, including *MIR160, MIR396, MIR397 *and *MIR408*, were each represented by a single member, while the others, including *MIR156, MIR166, MIR167 *and *MIR172*, each had two or three members identified (Figure 6 and Table 2). In absence of a small RNA database for *D. purpurea*, we are not able to reveal those mlncRNAs that produce species-specific miRNAs, but even so, the ratio (~0.06%) of miRNA-producing unigenes to total *D. purpurea *transcripts is much higher than the ratios (~0.01%) reported previously for other plant species, suggesting the importance of miRNAs in *D. purpurea *plants [66].

**Table 2:** Identification of 13 *D. purpurea *miRNAs

miRNA gene	miRNA sequence	Unigene ID	MFEI*	Length (bp)	454 reads
MIR156a	UGACAGAAGAGAGGGAGCAC	JO463945	0.99	494	6
MIR156b	UGACAGAAGAGAGUGAGCAC	FXAT9O005F5R10	1.11	209	1
MIR160	UGCCUGGCUCCUUGUAUGCCA	FXAT9O005F2EIB	1.18	425	1
MIR166a	UCGGACCAGGCUUCAUUCCUC	JO467104	0.82	356	5
MIR166b	UCGGACCAGGCUUCAUUCCCC	JO462464	0.74	595	6
MIR167a	UGAAGCUGCCAGCAUGAUCUA	JO465767	1.12	379	3
MIR167b	UGAAGCUGCCAGCAUGAUCUA	FXAT9O005F011M	1.07	518	1
MIR167c	UGAAGCUGCCAGCAUGAUCUG	FXAT9O005F5PKB	0.85	300	1
MIR172a	AGAAUCUUGAUGAUGCUGCAU	JO462274	1.25	610	4
MIR172b	AGAAUCUUGAUGAUGCUGCAU	FXAT9O005GBAO8	1.09	399	1
MIR396	UUCCACAGCUUUCUUGAACUG	FXAT9O005FNTBE	1.02	398	1
MIR397	CCAUUGAGUGCAGCGUUGAUG	FXAT9O005F4N47	1.33	438	1
MIR408	CUGCACUGCCUCUUCCCUGGC	JO460086	0.90	1095	45

* MFEI, minimal folding free energy index of the hairpin structures.

### Identification of 25 *D. purpurea *miRNA targets

The regulatory role of plant miRNAs was achieved mainly through targeting other RNA molecules for cleavage [35-38]. Perfect or near-perfect complementarities were found between plant miRNAs and their targets, allowing an effective prediction of target sequences through computation [69]. A useful web server for prediction of plant miRNA targets, known as psRNATarget, has been developed [70]. It searches potential miRNA targets using an improved iterative parallel Smith-Waterman algorithm and a weighted scoring schema [71]. Penalty scores were calculated for mismatched patterns in the miRNA:mRNA duplexes within a 20-base sequence window. Using psRNATarget and setting the penalty score cutoff threshold 0-3, we identified a total of 25 targets from our unigene set for seven *D. purpurea *miRNA families, which are *MIR156, MIR160, MIR166, MIR167, MIR172, MIR396*, and *MIR*408 (Table 3). The number of targets for each miRNA families range between one and seven. No targets were predicted for *MIR397*, indicating a very low expression level of miR397 targets in *D. purpurea *leaves.

**Table 3:** Identification of miRNA targets in *D. purpurea*

miRNA family	Function of targets	Unigene ID of targets (penalty score)	Length (bp)
MIR156	unknown	FXAT9O005F20XA (0.5)	369
		FXAT9O005GEKYB (3)	296
	PRLI-interacting factor L	FXAT9O005FO94E (3)	427
MIR160	ARF	JO460174 (1.5)	932
		FXAT9O005F7ZVB (2.5)	460
	glyoxalase I	JO466078 (3)	623
MIR166	unknown	FXAT9O005F0WN0 (2.5)	441
	VQ-motif containing protein	FXAT9O005FXWFT (3)	506
MIR167	unknown	FXAT9O005F7P6T (3)	463
MIR172	AP2	FXAT9O005FMQGW (1.5)	376
	serine/threonine kinase	FXAT9O005F9IN5 (2.5)	464
	hydrolase	FXAT9O005FV544 (3)	195
		FXAT9O005F4DOL (3)	489
	sec14 cytosolic factor	JO460887 (3)	1402
	unknown	FXAT9O005F2EA1 (3)	534
		JO465008 (3)	479
MIR408	KH domain-containing protein	FXAT9O005F4K0N (3)	319
		JO464508(3)	1292
MIR396	GRF	FXAT9O005FZB6N (2)	445
	global transcription factor	FXAT9O005FZLA6 (3)	453
	dead box ATP-dependent RNA	JO461297 (3)	703
	helicase	JO464891 (3)	398
	Heat shock protein	FXAT9O005F8VKM (2.5)	336
	unknown	FXAT9O005F0GS0 (3)	438
		FXAT9O005FSKAG (3)	373

As expected, some of the predicted targets are deeply conserved among various plant species. It includes two auxin response factor (*ARF*) genes regulated by miR160, an *APETALA2 *(*AP2*) gene targeted by miR172, and a growth-regulating factor (*GRF*) gene cleaved by miR396 (Table 3). All of them encode transcription factors that are important to plant development, suggesting the conserved roles of miRNAs in different plant species. Twenty one of the 25 predicted targets appear to be not conserved targets of miRNAs. It is consistent with the previous results showing species-specific targets for many conserved miRNAs in other plant species, such as *Populus trichocarpa *and *Pinus taeda *[23,72,73]. Among the twenty one targets, twelve are involved in metabolism, RNA process, transcriptional regulation and signal transduction, while the other nine are function-unknown, indicating possible new roles of miRNAs in *D. purpure*a (Table 3). Computational comparison of gene functions showed that many of these species-specific targets could be important in plant responses to biotic and abiotic stresses. For instance, the glyoxalase I gene targeted by miR160 has 75% identity with a member of *Arabidopsis *calmodulin-binding proteins, At1g08110, which is involved in plant responses to phytoprostane treatment [74,75]. The two KH domain-containing proteins regulated by miR408 show at least 75% identity with At5g56140 that is involved in light responses in *Arabidopsis *plants growing at low temperature [76]. The dead box ATP-dependent RNA helicase gene targted by miR396 is a member of a large gene family involved in defense responses against pathogen infection and various abiotic stresses [77-80]. The heat shock protein encoded by contig05310, a predicted target of miR396, shows 91% identity to *Arabidopsis *HSP90.1 and HSP81 that are involved in various biotic and abiotic stresses [81,82]. These results suggested the roles of miRNAs in the development and defense responses of *D. purpurea *plants. Further experimental validation of the predicted miRNA targets may help add new insights into the regulatory mechanisms of miRNAs in *D. purpurea*.

### Indentification of mlncRNA families based on sequence homology

Among the 2660 *D. purpurea *mlncRNA candidates with sizes at least 300 bp, 12 produce miRNAs (Table 2), while functions of the other 2648 mlncRNAs are poorly understood. As a first step toward elucidating the roles of these mlncRNAs, we searched sequence homology among them using BLASTN [58]. By applying an e-value cutoff of 10^-5 ^to the homolog recognition, we revealed that 320 of the 2648 mlncRNAs could be grouped into 140 families (see Additional file 4). Number of members for these mlncRNA families is between 2 and 8. The existence of mlncRNA families with multiple members could be a first indication that they actually are important in *D. purpurea*. No homologs were found for the other 2328 mlncRNAs, indicating that most of the mlncRNAs could be single copy. These results provide a basis for further demonstrating mlncRNA functions.

### Conservation of *D. purpurea *mlncRNAs

The conservation of mlncRNAs was analyzed by searching *D. purpurea *mlncRNA candidates against the NONCODE database of known npcRNAs using the BLASTN program [58,83]. An e-value cutoff of 10^-5 ^was applied. Among the 2648 non-microRNA-producing mlncRNA candidates, only eight were found to be conserved (see Additional file 5). It suggests the majority of *D. purpurea *mlncRNAs are species-specific. The low degree of evolutionary constraint of mlncRNAs was also found in other plant species including *M. truncatula, Arabidopsis*, wheat, and animals such as *Drosophila *and mouse, indicating low conservation of mlncRNAs is a common phenomenon in organisms [42,45,46,64,84]. Among the eight conserved mlncRNAs, two are homologues of GUT15, an npcRNA probably involved in hormone response in *Nicotiana tabacum *and *Arabidopsis *[85,86]. Functions of the other six conserved mlncRNAs are currently unknown.

### Tissue-specific expression of mlncRNAs in *D. purpurea*

**Figure 7:**
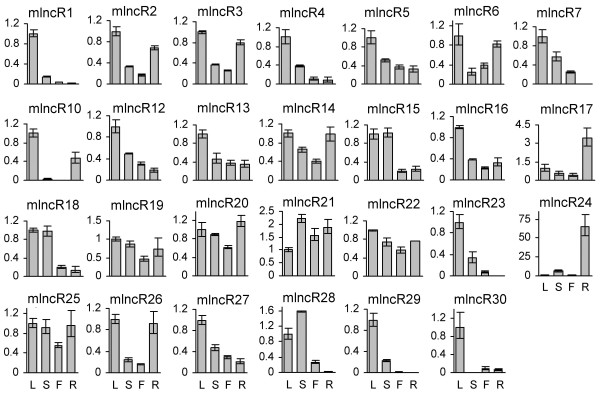
**Expression of mlncRNAs in leaves (L), stems (S), flowers (F) and roots (R) of one-year-old *D. purpurea *plants**. Fold changes of mlncRNA levels were shown. Expression levels were quantified by qPCR. Ubiquitin was used as a reference gene. The mlncRNA levels in leaves were arbitrarily set to 1. Error bars represent the standard deviations of three PCR replicates.

Except miR408-producing npcR9 (JO460086), expression patterns of the other 29 mlncRNAs with sizes over 700 bp were analyzed in one-year-old, greenhouse grown *D. purpurea *plants by quantitative real-time RT-PCR (qPCR). Ubiquitin gene was selected as a reference since it showed stable expression in the *D. purpurea *tissues analyzed compared with actin and 18S rRNA (see Additional file 6) [87]. Among the 29 mlncRNAs analyzed, two, including mlncR8 and mlncR11, were undetected, indicating low levels in the tissues analyzed. The other 27 were found to be expressed in at least one tissue (Figure 7). mlncR1, mlncR4, mlncR10, mlncR23, mlncR29 and mlncR30 are abundant in leaves, while mlncR17 and mlncR24 are expressed mainly in roots. The levels of mlncR2, mlncR3, mlncR6, mlncR14 and mlncR26 are higher in leaves and roots than stems and flowers, while mlncR15, mlncR18 and mlncR28 exhibit high expression in leaves and stems compared with that in flowers and roots. Differential expression was also found for the other mlncRNAs (Figure 7). It suggests the level of mlncRNAs is developmentally regulated and indicates the importance of mlncRNAs in *D. purpurea *growth and development.

### Identification of 24 cold- and 27 dehydration-responsive mlncRNAs

**Figure 8:**
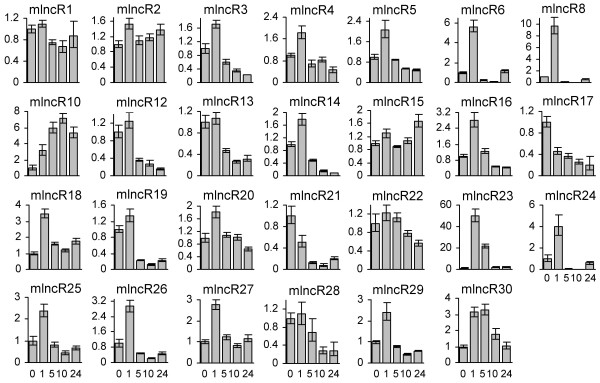
**Expression of mlncRNAs in *D. purpurea *plantlets treated with cold stress for 0, 1, 5, 10 and 24 hours**. Fold changes of mlncRNA levels were shown. Expression levels were quantified by qPCR. Ubiquitin was used as a reference gene. The mlncRNA levels in leaves were arbitrarily set to 1. Error bars represent the standard deviations of three PCR replicates.

**Figure 9:**
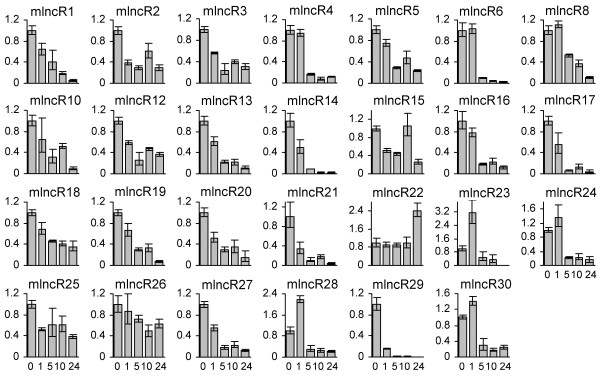
**Expression of mlncRNAs in *D. purpurea *plantlets treated with dehydration stress for 0, 1, 5, 10 and 24 hours**. Fold changes of mlncRNA levels were shown. Expression levels were quantified by qPCR. Ubiquitin was used as a reference gene. The mlncRNA levels in leaves were arbitrarily set to 1. Error bars represent the standard deviations of three PCR replicates.

*D. purpurea *plants tolerate various environmental stresses [1,8,9]. To address whether mlncRNAs are involved in plant response to cold and dehydration stresses, we analyzed the levels of 29 non-miRNA-producing mlncRNAs with sizes over 700 bp in *D. purpurea *plantlets treated with cold and dehydration for 1, 5, 10, 24 hours and compared them with the levels in untreated plantlets. We were able to detect 27 mlncRNAs in plantlets, except mlncR7, an mlncRNA detected in leaves, stems and flowers of one-year old mature plants, and mlncR11, which was also undetected in the analyzed tissues of mature plants (Figure 7, 8 and 9). *D. purpurea *mlncR7 exhibited opposite expression patterns with mlncR8, which expressed in plantlets but not in the analyzed tissues of one-year-old plants. It suggests that the expression of both mlncR7 and mlncR8 is developmentally regulated, while their biological functions are distinct. *D. purpurea *mlncR11 appears to play a temporary role in *D. purpurea *because it is undetected in all of the tissues analyzed.

Among the 27 mlncRNAs expressed in plantlets, 24 and 27 showed more than 2-fold changes between at least two time-points in plantlets treated with cold and dehydration, respectively, and thus was considered as cold- or dehydration-responsive mlncRNAs (Figure 8 and 9). It includes 3 responsive to dehydration only and 24 responsive to cold and dehydration, suggesting all of the mlncRNAs analyzed are stress-responsive and the majority is not only cold-responsive but also dehydration-responsive. It demonstrates the importance of mlncRNAs in stress responses and indicates the existence of a crosstalk among mlncRNAs in response to cold and dehydration stresses. The results of almost all analyzed mlncRNAs to be cold- and/or dehydration-responsive indicate that the plant materials were probably exposed to environmental stresses before being used for EST library construction.

Based on the expression patterns in response to cold stress, mlncRNAs can be roughly categorized into three major types (Figure 8). Type A mlncRNAs, including mlncR1, mlncR2 and mlncR15, showed less than 2-fold changes between any two time-points. These mlncRNAs appears to be not responsive to cold. Type B, such as mlncR17 and mlncR21, exhibited an immediate decrease after treatment for 1 hour. The decrease continued for at least 10 hours and then some of them, such as mlncR21, showed a trend of recovering to the level in untreated tissues. By contrast, type C mlncRNAs, accounting for more than 80% or 22 of the total 27 mlncRNAs, showed a rapid increase after treatment. The highest level was reached, for most type C mlncRNAs, at 1 h after stress, or in a few cases, at 5 (mlncR30) or 10 hours (mlncR10). After reaching to a maximum, the mlncRNA levels quickly declined to near, or in most cases, far below the levels in untreated tissues. Rapid changes of expression level indicate the importance of type B and type C mlncRNAs in cold-stress response, although the underlying biological functions can be distinct between type B and type C mlncRNAs.

Similarly, based on the expression patterns in response to dehydration stress, mlncRNAs can also be roughly classified into 3 groups (Figure 9). The level of group I mlncRNAs, such as mlncR22, kept constant within the first 10 hours of dehydration stress and then increased at 24 hours, showing a relatively slow response. Group II and group III mlncRNAs showed much quicker responses to dehydration stress. The expression pattern of group II mlncRNAs in response to dehydration is similar to type B mlncRNAs in response to cold stress, exhibiting a rapid decrease at 1 hour of stress (Figure 8 and 9). Except mlncR15, which showed an increase at 10 hours, down-regulation of most group II mlncRNAs lasted for at least 24 hours. Group II, consisting of 20 mlncRNAs, is much larger than type B which includes only two, although group II and type B mlncRNAs exhibited similar expression patterns in response to dehydration and cold stress, respectively. Group III consists of 6 mlncRNAs, including mlncR6, mlncR8, mlncR23, mlncR24, mlncR28 and mlncR30. Expression of mlncRNAs in this group was induced at 1 hour of stress and then immediately down-regulated to far below the level in untreated tissues. It is similar to the pattern of type C mlncRNAs in response to cold stress (Figure 8 and 9). The similarity of mlncRNA expression patterns in response to cold and dehydration suggests that the cold signaling network and the dehydration signaling network are probably overlapped.

### Identification of 417 protein-coding genes with regions significantly homologous or complementary to 375 mlncRNAs

Genes with conserved sequences usually show structural, functional or evolutionary relationships. In order to elucidate the relationship between mlncRNAs and protein-coding genes in *D. purpurea*, we searched our unigene set for protein-coding genes with regions significantly homologous or complementary to mlncRNAs using BLASTN and applying a generous e-value cutoff of 10^-5 ^to the BLAST homolog recognition [58]. A total of 417 protein-coding genes were identified (see Additional file 7). They showed sequence similarity to 375 mlncRNAs in coding sequences (CDS), untranslated regions (UTR) or CDS-UTR junctions. Among the 417 protein-coding genes, 373 each had only one corresponding mlncRNAs, while the others each had 2-9 hits, implying protein-coding genes can be related to one or several mlncRNAs. Similarly, among the 375 mlncRNAs, 51 each showed sequence similarity to 2-10 protein-coding genes, indicating some mlncRNAs are related to multiple protein-coding genes. The results demonstrate the complex relationship between mlncRNAs and protein-coding genes in *D. purpurea*. Among the 417 protein-coding genes, many encode known proteins involved in metabolism, signal transduction, gene regulation, stress response, protein folding and degradation, or nucleic acid and chromatin modification, indicating the importance of mlncRNAs in the growth and development of *D. purpurea *plants (Table 4 see Additional file 7).

**Table 4:** mlncRNAs shown sequence homology to secondary metabolism-related protein-coding genes

mlncRNA ID	Protein-coding gene ID	Enzyme name	Pathway involved
Contig03310 (mlncR8)	JO463639	4-Hydroxy-3-methylbut-2-en-1-yl diphosphate synthase	Terpenoid backbone biosynthesis
FXAT9O005F8J63 (mlncR31)	JO463507	Solanesyl diphosphate synthase	Biosynthesis of isoprenoid side chain of ubiquinone and plastoquinone
FXAT9O005FZSI2	FXAT9O005FZ5UJ	Dihydroflavonal-4-reductase	Flavonoid biosynthesis
FXAT9O005GD1W7	FXAT9O005GCP3D	Phytoene dehydrogenase	Carotenoid biosynthesis
FXAT9O005FYD4C	FXAT9O005FK4AG	Aromatic amino acid decarboxylase	Alkaloid biosynthesis

### Expression analysis of 4 protein-coding genes homologous or complementary to mlncRNAs

**Figure 10:**
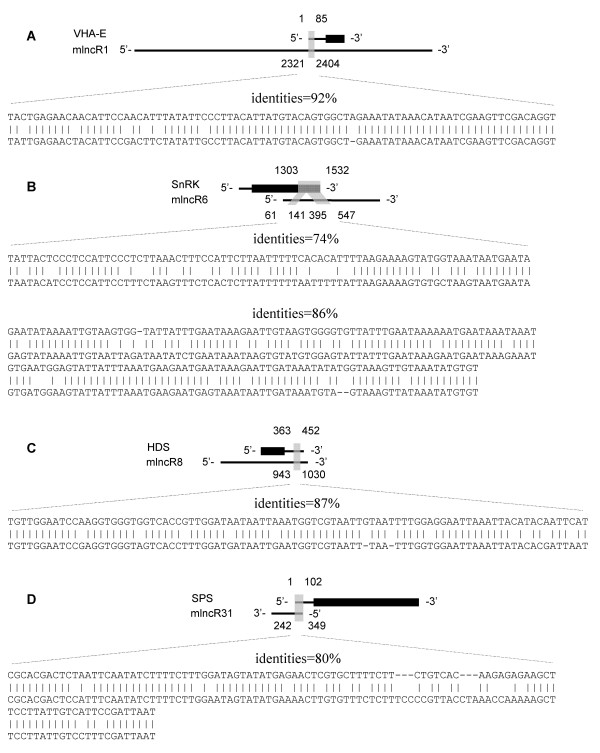
**Protein-coding genes homologous or complementary to mlncRNAs**. Protein-coding genes are presented by heavy black lines (coding sequences) and thin lines (nontranslated regions). mlncRNAs are represented by thin lines. The transcriptional direction of protein-coding genes was determined based on the direction of annotated proteins, while that of mlncRNAs was analyzed by 3' RACE. *VHA-E, SnRK *and *HDS *are homologous to mlncR1, mlncR6 and mlncR8, respectively (A, B and C). *SPS *shares an antisense homology with mlncR31 (D). Homologous or complementary regions are shaded in grey and the nucleotide positions are shown. Sequence identities and Watson-Crick pairing (vertical dashes) between protein-coding genes and the corresponding mlncRNAs are indicated.

Four mlncRNA/protein-coding gene pairs were selected for further analysis (Figure 10). It includes mlncR1 (JO460015)/vacuolar H^+^-ATP synthase subunit E gene (*VHA-E*, FXAT9O005FSUW), mlncR6 (JO460006)/SNF1-related protein kinase gene (*SnRK*, JO461197), mlncR8 (JO463019)/4-hydroxy-3-methylbut-2-en-1-yl diphosphate synthase gene (*HDS*, JO463639), and mlncR31 (FXAT9O005F8J63)/solanesyl diphosphate synthase gene (*SPS*, JO463507). We first determined the transcriptional direction of protein-coding genes based on the direction of annotated proteins, and then experimentally identified the transcriptional direction of mlncRNAs using 3' RACE. Results show that mlncR1 and mlncR6 and mlncR8 have the same transcriptional direction as their corresponding protein-coding genes in the conserved regions, suggesting these mlncRNAs are homologous to the corresponding protein-coding genes. Among them, mlncR1 and mlncR8 share homologous sequences with the 5' UTR of *VHA-E *and the 3' UTR of *HDS*, respectively, while mlncR6 has a region highly similar to the CDS of *SnRK*. In contrast, mlncR31 appears to be complementary to the 5' UTR of *SPS *gene (Figure 10).

**Figure 11:**
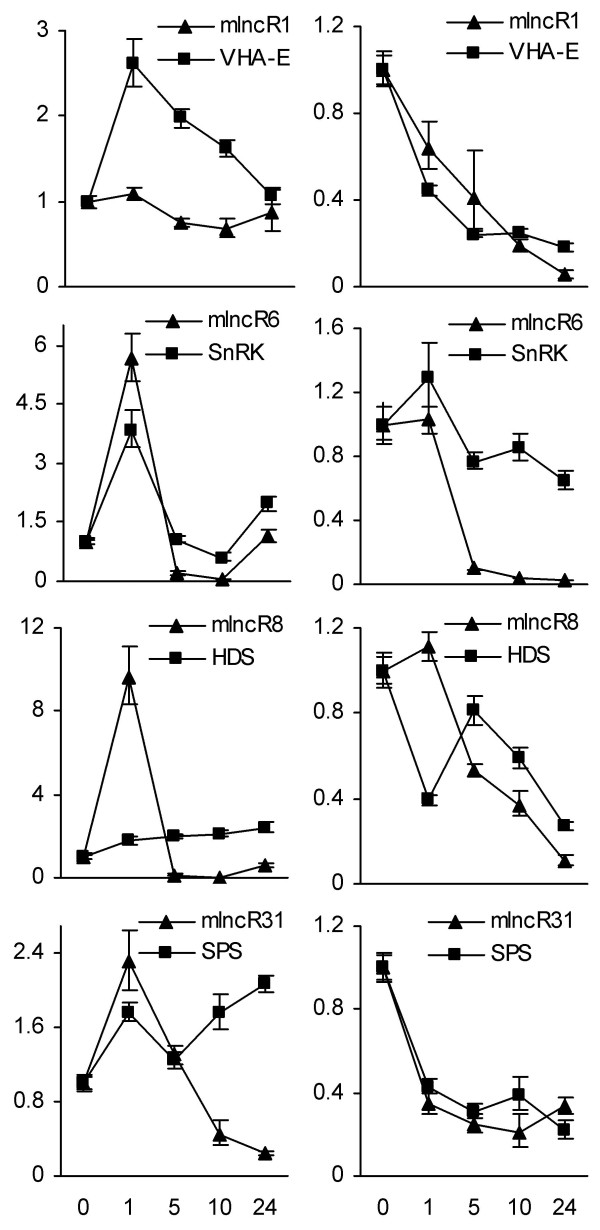
**Expression of mlncR1, mlncR6, mlncR8 and mlncR31 and the corresponding protein-coding genes, *VHA-E, SnRK, HDS *and *SPS*, in *D. purpurea *plantlets treated with cold (left panel) and dehydration (right panel) stresses for 0, 1, 5, 10 and 24 hours**. Fold changes of expression levels were shown. The levels of mlncRNAs and protein-coding genes were quantified by qPCR. Ubiquitin was used as a reference gene. The levels of mlncRNAs and protein-coding genes in untreated tissues were arbitrarily set to 1. Error bars represent the standard deviations of three PCR replicates.

We next analyzed the expression patterns of *VHA-E, SnRK, HDS *and *SPS *in response to cold and dehydration stresses and compared them with the corresponding mlncRNAs (Figure 11). In general, the expression of *VHA-E, SnRK *and *HDS *showed a positive correlation with the homologous mlncRNAs, mlncR1, mlncR6 and mlncR8, respectively, although variation was found at very early stage after treatment, such as 1 hour. The most conspicuous positive correlation of expression was found at 5, 10 and 24 hours of stress. It suggests the existence of a close relationship between protein-coding genes and the homologous mlncRNAs and indicates that some protein-coding genes can be regulated by mlncRNAs based on the sense:sense interaction through a currently unknown mechanism or the protein-coding genes and mlncRNAs may be regulated by the same cis/trans factors. In contrast, the expression of *SPS *gene showed a negative correlation with mlncR31 after 5 hours of stress, being most noticeable in plantlets treated with cold stress (Figure 11). It indicates the presence of sense:antisense interaction between protein-coding genes and the complementary mlncRNAs.

## Discussion

### Some mlncRNAs shown sense or antisense homology with protein-coding genes involved in secondary metabolism in *D. purpurea*

Transcriptomic analysis is an effective approach to discover genes particularly for those organisms without whole genome information, such as *D. purpurea*, an economically and ecologically important plant species. Through high-throughput 454 sequencing and subsequent assembly, we obtained a high-quality *D. purpurea *unigene set comprising a total of 23532 genes, which include 140 putatively involved in the biosynthesis of terpenoids/cardiac glycosides, the most effective drugs to treat heart failure during the past two centuries [1,4,5]. The 140 genes were grouped into 30 families, representing about 75% of total cardiac glycoside biosynthesis-associated gene families in *D. purpurea*. These sequence data greatly enhance our knowledge of cardiac glycoside biosynthesis and provides useful information for manipulating cardiac glycoside biosynthesis in *D. purpurea *through biotechnology. More importantly, we found that 5 mlncRNAs showed sense or antisense homology with 5 protein-coding genes involved in secondary metabolism, including one putatively involved in terpenoid biosynthesis (Table 4).

HDS is the penultimate enzyme of the seven-step DXP pathway, one of the two pathways generating the C5 backbone of all terpenoids including the hormone GA and CGs (see Additional file 8). It catalyzes the conversion of 2-C-methyl-D-erythritol 2,4-cyclodiphosphate into 1-hydroxy-2-methyl-2-butenyl 4-diphosphate and is encoded by a single gene in various plants, including *Arabidopsis, Hevea brasiliensis *and *Ginkgo biloba *[88-91]. Through sequence similarity search and subsequent transcriptional direction analysis, we found that the 3' UTR of *D. purpurea HDS *gene contained a 90 bp-region with 87% identities to an mlncRNA, mlncR8 (Figure 10). The expression of *HDS *and mlncR8 was positively correlated at 5, 10 and 24 hours of cold and dehydration stresses (Figure 11). It indicates the existence of close relationship between *HDS *and mlncR8 through a currently unknown mechanism.

*SPS *gene involved in the biosynthesis of ubiquinone and plastoquinone is related to mlncR31. It utilizes farnesyl diphosphate (FPP) and geranylgeranyl diphosphate (GGPP) as substrates and is responsible for the biosynthesis of isoprenoid side chain of ubiquinone and plastoquinone in *Arabidopsis *[92]. The 5' UTR of *D. purpurea SPS *gene harbors a 102 bp-region highly complementary to mlncR31 (Figure 10). The expression of *SPS *gene is negatively correlated with mlncR31, which is most noticeable in plantlets treated with cold stress for 5, 10 and 24 hours (Figure 11), indicating *SPS *gene is regulated by mlncR31 through RNA-mediated gene silencing in *D. purpurea *[93-95]. Both ubiquinone and plastoquinone are electron carriers. Plastoquinone is a member of the photosynthetic electron transport chain in chloroplast, while ubiquinone is a component of respiratory electron transport chain in mitochondria. Thus, mlncR31 may be important in maintaining the activities of plant cells under environmental stress conditions.

In addition, mlncRNAs are probably involved in the biosynthesis of flavonoid, carotenoid and alkaloid (Table 4). The mlncRNA, FXAT9O005FZSI2, share homology with a unigene (FXAT9O005FZ5UJ) encoding dihydroflavonal-4-reductase (DFR), which catalyze the reduction of dihydroflavonol into flavan-3,4-diol (leucoanthocyanin), a key step in the biosynthesis of anthocyanins, proanthocyanidins and other flavonoids important for plant survival and human nutrition [96]. FXAT9O005GD1W7 appears the other mlncRNA involved in secondary metabolism. It shows sequence similarity to a unigene (FXAT9O005GCP3D) encoding phytoene dehydrogenase (PDS). PDS, which catalyzes the desaturation of phytoene into phytofluene, is a rate-limiting enzyme in carotenoid biosynthesis in plants [97]. The mlncRNA probably involved in alkaloid biosynthesis is FXAT9O005FYD4C, which shares homology with the coding region of aromatic amino acid decarboxylase (*AADC*) gene (FXAT9O005FK4AG). AADC catalyzes the decarboxylation of tyrosine into tyramine, a key step of plant alkaloid biosynthesis [98,99].

Taken together, these results strongly suggest the importance of mlncRNAs in secondary metabolism in *D. purpurea*.

### *D. purpurea *mlncRNAs exhibit species-specific characteristics and are important in plant development and stress responses

Using a computational mlncRNA identification pipeline, we identified 2660 *D. purpurea *mlncRNA candidates, of which only about 12% can be grouped into families with at least two members in a family, while the others are single gene family members. Furthermore, the conservation of mlncRNAs is very low. Searching the NONCODE database of known npcRNAs, we found that only about 0.3% of the mlncRNAs analyzed was conserved, suggesting the vast majority of *D. purpurea *mlncRNAs are species-specific. These results are consistent with those obtained from other plant species including *M. truncatula, Arabidopsis*, wheat, and animals such as *Drosophila *and mouse [42,45,46,64,84], indicating mlncRNAs may be evolved with a low degree of constraint and many of them are probably undergoing frequent birth and death. It is particularly true for the mlncRNAs which produce non-conserved microRNA families represented by single genes [100].

In this study, we identified a total of 13 microRNA-producing mlncRNAs based on the conservation of miRNA sequences and the secondary structure of unigenes [66-68]. The mature microRNAs were classified into eight families, including *MIR156, MIR160, MIR166, MIR167, MIR172, MIR396, MIR397 *and *MIR408 *(Figure 6, Table 2). They were predicted to regulate at least 25 protein-coding genes, of which 4 encode conserved transcriptional factors, including two ARFs, an AP2 and a GRF (Table 3). ARFs play regulatory roles in the development of plant organs, such as seed, gynoecium, embryo, hypocotyl, root and petal in *Arabidopsis *[101-107]. AP2 is involved in floral transition and floral development [108]. GRF control cell proliferation in *Arabidopsis *leaves [109,110]. It suggests the importance of microRNAs in the development of *D. purpurea *plants. Additionally, among the 417 protein-coding genes shown sense or antisense homology with mlncRNAs, several, such as JO464993, FXAT9O005FR21D, FXAT9O005GE5AF, FXAT9O005F8DZ7, FXAT9O005F4WF9 and FXAT9O005GCSS5, encode transcription factors and other development-related proteins, indicating some non-microRNA-producing mlncRNAs are also probably involved in the development of *D. purpurea *plants (see Additional file 7). It is consistent with the findings showing the involvement of mlncRNA (Zm401) in pollen development in *Zea mays *and the regulatory role of mlncRNA (enod40) in symbiotic nitrogen-fixing nodule formation in legumes [49,111,112].

Expressional analysis of mlncRNAs allowed us to identify 24 cold- and 27 dehydration-responsive mlncRNAs, suggesting the importance of mlncRNAs in stress responses in *D. purpurea *(Figure 8 and 9). In addition, the majority of mlncRNAs analyzed were responsive to both cold and dehydration and many of them exhibited similar expression patterns under cold and dehydration conditions, indicating the cold signaling network and the dehydration signaling network are probably overlapped. Stress responses of mlncRNAs have been reported previously in various plants, including *Arabidopsis *[39,44,113] and wheat [46]. For example, *Arabidopsis *IPS1 and *M. truncatula *Mt4 mlncRNAs are involved in phosphate starvation through fine-tuning the expression of miR399/PHO2 gene [54,56,113]. *Arabidopsis *COOLAIR (cold induced long antisense intragenic RNA) and COLDAIR (COLD ASSISTED INTRONIC NONCODING RNA) play significant roles in cold response and are required for the vernalization-mediated epigenetic repression of FLC [44,55]. Several wheat mlncRNAs are responsive to powdery mildew infection and/or heat stress [46]. Thus, mlncRNAs seem to be very important for the whole plant kingdom to survive in the stressful environments.

### Functional mechanisms of mlncRNAs in *D. purpurea*

mlncRNAs have been found to interact with RNA, DNA and protein molecules [13,114,115]. A small portion of the identified *D. purpurea *mlncRNAs produce conserved microRNAs. Since no small RNA sequences are available and experimental setup does not allow us to detect the processed miRNAs, we are not able to identify those producing non-conserved microRNAs from the set of *D. purpurea *mlncRNA candidates. However, it is rational to believe the existence of non-conserved microRNA-producing *D. purpurea *mlncRNAs based on the increasing number of non-conserved microRNAs in the miRBase [67]. These mlncRNAs play crucial roles in organ development and defense responses through producing conserved or non-conserved microRNAs, which target other RNA molecules for cleavage, or, in a few cases, for translational repression [35-38]. Additionally, some mlncRNAs play regulatory roles in plants through generating siRNAs. These mlncRNAs can be antisense transcripts of protein-coding genes, ta-siRNA precursors, repeat-associated transcripts, or transcripts sharing antisense homology with protein-coding genes [116-118]. It is also possible that some mlncRNAs may function through inhibiting the action of microRNAs. For example, the *Arabidopsis *mlncRNA, IPS1, contains a 24 nt region complementary to miR399, a microRNA involved in phosphate starvation by targeting *PHO2 *gene for cleavage. IPS1 mimics *PHO2*, except that the base-pairing is interrupted by a mismatched loop at the miR399 cleavage. Therefore, IPS1 is not cleaved but instead sequesters miR399, resulting in inhibition of miR399 activity on *PHO2 *[113]. The siRNA-generating mlncRNAs and the mimics of microRNA targets in our mlncRNA set remain to be identified.

In this study, we predicted a total of 2660 *D. purpurea *mlncRNA candidates using a computational mlncRNA identification pipeline. According to the sequence similarity between mlncRNAs and protein-coding genes and the location of homologous regions in mlncRNAs and protein-coding genes, the majority of mlncRNAs candidates appear to be not the fragments of UTRs of protein-coding genes (Figure 11; see Additional file 7). Among the 2660 mlncRNA candidates, 375 mlncRNAs share sense or antisense homology with 417 protein-coding genes, indicating that some mlncRNAs and the corresponding protein-coding gene are probably homologous genes derived from a common ancestral gene, or these mlncRNAs are new born genes originated from the corresponding protein-coding genes, as the case of long non-coding RNA, *Xist*, and the protein-coding gene, *Lnx3*, in mammals [119,120]. The mlncRNAs sharing antisense homology with protein-coding genes, such as *D. purpurea *mlncR31, seems to play a role in post-transcriptional regulation of the corresponding protein-coding genes. It is evidenced by the negative correlation of expression between mlncR31 and the *SPS *gene after 5 hours of stress (Figure 11). The roles in post-transcriptional regulation have been previously reported for some long non-coding RNAs identified from various animal species, such as human and mouse [13,93,95]. It is not well-known for the mechanisms of mlncRNAs acting on protein-coding genes with sense homology, such as mlncR1, mlncR6 and mlncR8 and their homologous protein-coding genes, *VHA-E, SnRK *and *HDS*, respectively. One of the possible mechanisms is co-suppression, a phenomenon in plants in which a sense transgene sometimes cause suppression of both the endogenous gene and the transgene [121-123]. Further identification of the interaction mechanism between mlncRNAs and protein-coding genes will give us a much clearer picture of how mlncRNAs function in *D. purpurea*.

## Conclusions

The comprehensive analysis of transcriptome data from high-throughput sequencing allow us to discover many novel unigenes involved in the biosynthesis of secondary metabolites, such as cardiac glycosides, ubiquinone, plastoquinone, flavonoid, carotenoid and alkaloid, and to identify a large number of mlncRNA candidates in *D. purpurea*. Detailed analysis suggests that the majority of *D. purpurea *mlncRNAs are species-specific and most mlncRNA families are single gene families. These mlncRNAs exhibited tissue-specific expression and responded to cold and dehydration stresses. Since at least 24 mlncRNAs were found to be not only cold-responsive but also dehydration-responsive, a crosstalk could exist among mlncRNAs in response to cold and dehydration stresses. The identified mlncRNAs appears to be involved in many aspects, such as plant development, stress response and secondary metabolism. The regulatory role is associated with protein-coding genes sharing sequence similarity with the mlncRNAs. These results provide novel and significant information for understanding the biosynthesis of secondary metabolites and the function of mlncRNAs. In addition, a large number of RNAseq data of *D. purpurea *have been recently released in the NCBI SRA database under the series identifier SRP006029, which provides an additional source for the discovery of genes and mlncRNAs in *D. purpurea*.

## Methods

### Plant materials

Leaves, stems, flowers and roots were collected from one-year-old, greenhouse grown *D. purpurea *'GIANT SHIRLY' plants and then used for EST library construction and tissue-specific expression analysis of mlncRNAs. For cold and dehydration treatments, *D. purpurea *seeds were sterilized in 70% ethanol for 1 min and then in 0.1% mercuric chloride for 8 min. After washing three times in sterile water, the seeds were grown on MS medium supplemented with 3% sucrose and 0.9% agar for 3 weeks at 25°C under 14 hours light:10 hours dark. The plantlets were treated at 4°C, or removed from the agar and then dehydrated in glass dishes at 25°C under dim light, for 0, 1, 5, 10, 24 hours. Six seedlings were collected in liquid nitrogen for each example.

### EST library construction and 454 sequencing

Total RNA was extracted from *D. purpurea *leaves using the Universal Plant Total RNA Extraction kit (Bioteke). mRNA was isolated from total RNA using the Oligotex mRNA kit (Qiagen). Single-stranded cDNA was prepared from mRNA using the SMART cDNA Synthesis kit (Clontech). Double-stranded cDNA was amplified by PCR polymerase Advantage II (Clontech). PCR products less than 300 bp in length were removed using the PureLink™ PCR Purification kit (Invitrogen). Pyrosequencing was performed by 454 Life Sciences using the Genome Sequencer FLX System.

### EST assembly, annotation and classification

The GS-FLX Software (454 Life Sciences, Roche) was used for trimming adapters and filtering low-quantity sequences. High-quantity reads were assembled into unigenes by the GS *De Novo *Assembler Software, an application of the GS FLX Software. The unigenes obtained were annotated by sequence similarity searches against the NCBI non-redundant (Nr) protein database using the Basic Local Alignment Search Tool (BLAST) [58]. A generous e-value cutoff of 10^-5 ^was applied to the BLAST homolog recognition. Gene Ontology (GO) terms were assigned using InterProScan [59].

### mlncRNA identification

The procedure for computational mlncRNA identification is outlined in Figure 4. Unigenes without Nr annotation and with sizes at least 300 bp were selected for open-reading frame (ORF) prediction using ESTScan 2 [124]. Computational prediction was performed on a local server through batch analysis using the default parameters. A cutoff of 100 amino acids was applied to distinguish mlncRNAs from protein-coding transcripts as described [43]. Housekeeping npcRNAs were searched against Rfam 10.0 (January 2010) using BLASTN [58,125]. Sequence homology among the *D. purpurea *mlncRNA candidates was analyzed using BLASTN [58]. The conservation of mlncRNAs was analyzed by searching *D. purpurea *mlncRNA candidates against the NONCODE database of known npcRNAs using the BLASTN program [58,83]. An e-value cutoff of 10^-5 ^was applied to the homolog recognition.

### Analysis of conserved miRNAs

Plant microRNA sequences were downloaded from miRBase (Released 17, http://www.mirbase.org/) [67]. Unigenes with regions no more than three mismatches to known microRNAs were searched against the *D. purpurea *unigene set using BLASTN [58]. A word size of 4 was used as described [126]. The secondary structures were predicted using mfold [68]. Criteria described by Meyers et al (2008) were applied to annotate *D. purpurea *microRNAs. The minimal folding free energy index (MFEI) was calculated as described previously [127].

### Prediction of microRNA targets

Prediction of microRNA targets was performed on the psRNATarget Server using the default parameters [70].

### qPCR

Total RNA was extracted from plant tissues using the Concert™ Plant RNA Reagent (Invitrogen) and then digested with RNase-free DNase I (Promega) to remove the genomic DNA contamination. Reverse transcription was performed on 2 μg total RNA for each example by 200 U SuperScript III Reverse Transcriptase (Invitrogen) in a 20 μl volume. The reaction was carried out at 65°C for 5 minutes, 50°C for 60 minutes and 70°C for 15 minutes. The resulting cDNA was diluted to 1,600 μl with sterile water. qPCR was carried out in triplicate reactions using the BIO-RAD CFX system (BIO-RAD). Gene-specific primers were listed in (see Additional file 9). Ubiquitin gene was selected as a reference. PCR was carried out in a 20 μl volume containing 2 μl diluted cDNA, 250 nM forward primer, 250 nM reverse primer, and 1 × SYBR Premix Ex Taq II (TaKaRa) using the following conditions: 95°C for 3 min, 40 cycles of 95°C for 15 sec, 60°C for 15 sec and 72°C for 15 sec. Specificity of the amplification was verified by the Bio-Rad CFX Manage software based on dissociation curves. The results from gene-specific amplification were analyzed using the comparative Cq method which uses an arithmetic formula, 2^-ΔΔCq^, to achieve results for relative quantification [128]. Cq represents the threshold cycle.

### 3' RACE for determining the transcriptional direction of mlncRNAs

3' RACE was performed on total RNA isolated from *D. purpurea *plantlets treated with cold stress for 1 hour using the GeneRacer kit (Invitrogen). PCR amplifications were carried out using the GeneRacer 3' primer and the nesting gene-specific primers. Nested PCR amplifications were performed using the GeneRacer 3' nested primer and the nested gene-specific primers (see Additional file 10). PCR products were purified, cloned and sequenced.

## Abbreviations

**3βHSD: **Δ^5^-3β-hydroxysteroid dehydrogenase; **5β-POR: **progesterone 5β-reductase; **AACT: **acetyl-CoA acetyltransferases; **AADC: **aromatic amino acid decarboxylase; **AP2: **APETALA2; **ARF: **auxin response factor; **BLAST: **Basic Local Alignment Search Tool; **BLASTN: **nucleotide BLAST; **CDS: **coding sequences; **CGH:**16'-O-glucohydrolases; **DFR: **dihydroflavonal-4-reductase: **Dp21MaT: **malonyl-coenzyme A: 21-hydroxypregnane 21-*O*-malonyltransferas: **EST: **expressed sequence tag; **FLC: **FLOWERING LOCUS C; **FPP: **farnesyl diphosphate; **GGPP: **geranylgeranyl diphosphate; **GO: **Gene Ontology; **GRF: **growth-regulating factor; **GT: **glycosyltransferases; **HDS: **4-hydroxy-3-methylbut-2-en-1-yl diphosphate synthase; **HMGS: **3-hydroxyl-3-methyl-glutaryl-CoA synthases; **KEGG: **Kyoto Encyclopedia of Genes and Genomes database; **lncRNAs: **long npcRNAs **;MCT: **2-C-methyl-D-erythritol 4-phosphate cytidylyltransferases; **MEP: **2-C-methyl-D-erythritol 4-phosphate; **miRNA: **microRNA; **mlncRNA: **mRNA-like npcRNA; **MVA: **mevalonate; **ncRNAs: **noncoding RNAs; **npcRNAs: **non-protein-coding RNAs; **Nr: **non-redundant; **ORF: **open-reading frame; **PDS:**phytoene dehydrogenase; **psRNATarget: **A Plant Small RNA Target Analysis Server; **RACE: **rapid amplification of cDNA ends; **RT-PCR: **reverse transcription-RCR; **siRNAs: **small interfering RNAs; **snoRNAs: **Small nucleolar RNAs; **SnRK: **SNF1-related protein kinase; **snRNAs: **small nuclear ribonucleic acid; **SPS: **solanesyl diphosphate synthase; **sRNAs: **small RNAs; **ta-siRNA: **trans small interfering RNAs; **UTR: **untranslated regions; **VHA-E: **vacuolar H^+^-ATP synthase subunit E.

## Competing interests

The authors declare that they have no competing interests.

## Authors' contributions

BW analyzed the data, performed qRT-PCR and RACE, and participated in writing the manuscript. YL and HL constructed the EST library. YL, HY, YM and LY participated in data analysis. SC participated in designing the experiment. SL designed the experiment and wrote the manuscript. All authors have read and approved the version of manuscript.

## Supplementary Material

Additional file 1 **Unigenes probably involved in cardiac glycoside biosynthesis**. Complete set of unigenes probably involved in cardiac glycoside biosynthesis. Click here for file

Additional file 2 **The 25 most abundant unigenes in *D. purpurea***. Complete set of the 25 most abundant unigenes in *D. purpurea*. Click here for file

Additional file 3 **Housekeeping npcRNAs identified**. Complete set of the housekeeping npcRNAs identified. Click here for file

Additional file 4 ***D. purpurea *mlncRNA families**. Complete set of the *D. purpurea *mlncRNA families. Click here for file

Additional file 5 **Conserved mlncRNAs**. Complete set of the conserved mlncRNAs. Click here for file

Additional file 6 **Expression of housekeeping genes**. (A) Cq value of ubiquitin, actin and 18s rRNA in leaves (L), stems (S), flowers (F) and roots (R) of one-year-old *D. purpurea *plants and plantlets treated with cold (C0, C1, C5, C10 and C24) and dehydration (D0, D1, D5, D10 and D24) stresses for 0, 1, 5, 10 and 24 hours. Expression levels were quantified by qPCR. The mean Cq value of three PCR replicates is shown. (B) The expression stability value (M) was calculated by geNorm software [87]. Click here for file

Additional file 7 **Protein-coding genes significantly homologous or complementary to mlncRNAs**. Complete set of the protein-coding genes significantly homologous or complementary to mlncRNAs. Click here for file

Additional file 8 **The putative biosynthetic pathway of cardiac glycosides in *Digitalis purpurea***. The pathway roughly comprises terpenoid backbone biosynthesis, steroid biosynthesis and cardenolide biosynthesis three stages. The enzymes with corresponding unigenes are framed. Click here for file

Additional file 9 **Primers used in qPCR**. Complete set of the primers used in qPCR. Click here for file

Additional file 10 **Primers used for indentifying the transcriptional direction of mlncRNAs**. Complete set of the primers used for indentifying the transcriptional direction of mlncRNAs. Click here for file

## References

[B1] Warren B Digitalis purpurea Am J Cardiol 2005 95 544 10.1016/j.amjcard.2004.09.063 15695152

[B2] Lin CC Yang CC Phua DH Deng JF Lu LH An outbreak of foxglove leaf poisoning J Chin Med Assoc 2010 73 97 100 10.1016/S1726-4901(10)70009-5 20171590

[B3] Maffe S Cucchi L Zenone F Bertoncelli C Beldi F Colombo ML Bielli M Paino AM Parravicini U Paffoni P Dellavesa P Perucca A Pardo NF Signorotti F Didino C Zanetta M *Digitalis *must be banished from the table: a rare case of acute accidental *Digitalis *intoxication of a whole family J Cardiovasc Med (Hagerstown) 2009 10 727 732 10.2459/JCM.0b013e32832c2314 19491701

[B4] Hauptman PJ Garg R Kelly RA Cardiac glycosides in the next millennium Prog Cardiovasc Dis 1999 41 247 254 10.1053/pcad.1999.0410247 10362347

[B5] Kuate SP Padua RM Eisenbeiss WF Kreis W Purification and characterization of malonyl-coenzyme A: 21-hydroxypregnane 21-O-malonyltransferase (Dp21MaT) from leaves of *Digitalis purpurea *L Phytochemistry 2008 69 619 626 10.1016/j.phytochem.2007.08.025 17945319

[B6] Lopez-Lazaro M Palma De La Pena N Pastor N Martin-Cordero C Navarro E Cortes F Ayuso MJ Toro MV Anti-tumour activity of *Digitalis purpurea *L. subsp. heywoodii Planta Med 2003 69 701 704 10.1055/s-2003-42789 14531018

[B7] Lesney MS Flowers for the heart Mod Drug Discov 2002 5 46 48

[B8] Sletvold N Rydgren K Population dynamics in *Digitalis purpurea*: the interaction of disturbance and seed bank dynamics J Ecol 2007 95 1346 1359

[B9] Bruelheide H Heinemeyer A Climatic factors controlling the eastern and altitudinal Bruelheide boundary of *Digitalis purpurea *L. in Germany Flora 2002 197 475 490

[B10] Gavidia I Tarrio R Rodriguez-Trelles F Perez-Bermudez P Seitz HU Plant progesterone 5beta-reductase is not homologous to the animal enzyme. Molecular evolutionary characterization of P5betaR from *Digitalis purpurea* Phytochemistry 2007 68 853 864 10.1016/j.phytochem.2006.11.019 17184799

[B11] Herl V Frankenstein J Meitinger N Muller-Uri F Kreis W Delta 5-3beta-hydroxysteroid dehydrogenase (3 beta HSD) from *Digitalis lanata*. Heterologous expression and characterisation of the recombinant enzyme Planta Med 2007 73 704 710 10.1055/s-2007-981537 17564944

[B12] Perez-Bermudez P Garcia AA Tunon I Gavidia I *Digitalis purpurea *P5 beta R2, encoding steroid 5 beta-reductase, is a novel defense-related gene involved in cardenolide biosynthesis New Phytol 2010 185 687 700 10.1111/j.1469-8137.2009.03080.x 19895417

[B13] Ponting CP Oliver PL Reik W Evolution and functions of long noncoding RNAs Cell 2009 136 629 641 10.1016/j.cell.2009.02.006 19239885

[B14] Bertone P Stolc V Royce TE Rozowsky JS Urban AE Zhu X Rinn JL Tongprasit W Samanta M Weissman S Gerstein M Snyder M Global identification of human transcribed sequences with genome tiling arrays Science 2004 306 2242 2246 10.1126/science.1103388 15539566

[B15] Birney E Stamatoyannopoulos JA Dutta A Guigo R Gingeras TR Margulies EH Weng Z Snyder M Dermitzakis ET Thurman RE Kuehn MS Taylor CM Neph S Koch CM Asthana S Malhotra A Adzhubei I Greenbaum JA Andrews RM Flicek P Boyle PJ Cao H Carter NP Clelland GK Davis S Day N Dhami P Dillon SC Dorschner MO Fiegler H Giresi PG Goldy J Hawrylycz M Haydock A Humbert R James KD Johnson BE Johnson EM Frum TT Rosenzweig ER Karnani N Lee K Lefebvre GC Navas PA Neri F Parker SC Sabo PJ Sandstrom R Shafer A Vetrie D Weaver M Wilcox S Yu M Collins FS Dekker J Lieb JD Tullius TD Crawford GE Sunyaev S Noble WS Dunham I Denoeud F Reymond A Kapranov P Rozowsky J Zheng D Castelo R Frankish A Harrow J Ghosh S Sandelin A Hofacker IL Baertsch R Keefe D Dike S Cheng J Hirsch HA Sekinger EA Lagarde J Abril JF Shahab A Flamm C Fried C Hackermuller J Hertel J Lindemeyer M Missal K Tanzer A Washietl S Korbel J Emanuelsson O Pedersen JS Holroyd N Taylor R Swarbreck D Matthews N Dickson MC Thomas DJ Weirauch MT Gilbert J Drenkow J Bell I Zhao X Srinivasan KG Sung WK Ooi HS Chiu KP Foissac S Alioto T Brent M Pachter L Tress ML Valencia A Choo SW Choo CY Ucla C Manzano C Wyss C Cheung E Clark TG Brown JB Ganesh M Patel S Tammana H Chrast J Henrichsen CN Kai C Kawai J Nagalakshmi U Wu J Lian Z Lian J Newburger P Zhang X Bickel P Mattick JS Carninci P Hayashizaki Y Weissman S Hubbard T Myers RM Rogers J Stadler PF Lowe TM Wei CL Ruan Y Struhl K Gerstein M Antonarakis SE Fu Y Green ED Karaoz U Siepel A Taylor J Liefer LA Wetterstrand KA Good PJ Feingold EA Guyer MS Cooper GM Asimenos G Dewey CN Hou M Nikolaev S Montoya-Burgos JI Loytynoja A Whelan S Pardi F Massingham T Huang H Zhang NR Holmes I Mullikin JC Ureta-Vidal A Paten B Seringhaus M Church D Rosenbloom K Kent WJ Stone EA Batzoglou S Goldman N Hardison RC Haussler D Miller W Sidow A Trinklein ND Zhang ZD Barrera L Stuart R King DC Ameur A Enroth S Bieda MC Kim J Bhinge AA Jiang N Liu J Yao F Vega VB Lee CW Ng P Yang A Moqtaderi Z Zhu Z Xu X Squazzo S Oberley MJ Inman D Singer MA Richmond TA Munn KJ Rada-Iglesias A Wallerman O Komorowski J Fowler JC Couttet P Bruce AW Dovey OM Ellis PD Langford CF Nix DA Euskirchen G Hartman S Urban AE Kraus P Van Calcar S Heintzman N Kim TH Wang K Qu C Hon G Luna R Glass CK Rosenfeld MG Aldred SF Cooper SJ Halees A Lin JM Shulha HP Xu M Haidar JN Yu Y Iyer VR Green RD Wadelius C Farnham PJ Ren B Harte RA Hinrichs AS Trumbower H Clawson H Hillman-Jackson J Zweig AS Smith K Thakkapallayil A Barber G Kuhn RM Karolchik D Armengol L Bird CP de Bakker PI Kern AD Lopez-Bigas N Martin JD Stranger BE Woodroffe A Davydov E Dimas A Eyras E Hallgrimsdottir IB Huppert J Zody MC Abecasis GR Estivill X Bouffard GG Guan X Hansen NF Idol JR Maduro VV Maskeri B McDowell JC Park M Thomas PJ Young AC Blakesley RW Muzny DM Sodergren E Wheeler DA Worley KC Jiang H Weinstock GM Gibbs RA Graves T Fulton R Mardis ER Wilson RK Clamp M Cuff J Gnerre S Jaffe DB Chang JL Lindblad-Toh K Lander ES Koriabine M Nefedov M Osoegawa K Yoshinaga Y Zhu B de Jong PJ Identification and analysis of functional elements in 1% of the human genome by the ENCODE pilot project Nature 2007 447 799 816 10.1038/nature05874 PMC2212820 17571346

[B16] Carninci P Kasukawa T Katayama S Gough J Frith MC Maeda N Oyama R Ravasi T Lenhard B Wells C Kodzius R Shimokawa K Bajic VB Brenner SE Batalov S Forrest AR Zavolan M Davis MJ Wilming LG Aidinis V Allen JE Ambesi-Impiombato A Apweiler R Aturaliya RN Bailey TL Bansal M Baxter L Beisel KW Bersano T Bono H Chalk AM Chiu KP Choudhary V Christoffels A Clutterbuck DR Crowe ML Dalla E Dalrymple BP de Bono B Della Gatta G di Bernardo D Down T Engstrom P Fagiolini M Faulkner G Fletcher CF Fukushima T Furuno M Futaki S Gariboldi M Georgii-Hemming P Gingeras TR Gojobori T Green RE Gustincich S Harbers M Hayashi Y Hensch TK Hirokawa N Hill D Huminiecki L Iacono M Ikeo K Iwama A Ishikawa T Jakt M Kanapin A Katoh M Kawasawa Y Kelso J Kitamura H Kitano H Kollias G Krishnan SP Kruger A Kummerfeld SK Kurochkin IV Lareau LF Lazarevic D Lipovich L Liu J Liuni S McWilliam S Madan Babu M Madera M Marchionni L Matsuda H Matsuzawa S Miki H Mignone F Miyake S Morris K Mottagui-Tabar S Mulder N Nakano N Nakauchi H Ng P Nilsson R Nishiguchi S Nishikawa S Nori F Ohara O Okazaki Y Orlando V Pang KC Pavan WJ Pavesi G Pesole G Petrovsky N Piazza S Reed J Reid JF Ring BZ Ringwald M Rost B Ruan Y Salzberg SL Sandelin A Schneider C Schonbach C Sekiguchi K Semple CA Seno S Sessa L Sheng Y Shibata Y Shimada H Shimada K Silva D Sinclair B Sperling S Stupka E Sugiura K Sultana R Takenaka Y Taki K Tammoja K Tan SL Tang S Taylor MS Tegner J Teichmann SA Ueda HR van Nimwegen E Verardo R Wei CL Yagi K Yamanishi H Zabarovsky E Zhu S Zimmer A Hide W Bult C Grimmond SM Teasdale RD Liu ET Brusic V Quackenbush J Wahlestedt C Mattick JS Hume DA Kai C Sasaki D Tomaru Y Fukuda S Kanamori-Katayama M Suzuki M Aoki J Arakawa T Iida J Imamura K Itoh M Kato T Kawaji H Kawagashira N Kawashima T Kojima M Kondo S Konno H Nakano K Ninomiya N Nishio T Okada M Plessy C Shibata K Shiraki T Suzuki S Tagami M Waki K Watahiki A Okamura-Oho Y Suzuki H Kawai J Hayashizaki Y The transcriptional landscape of the mammalian genome Science 2005 309 1559 1563 10.1126/science.1112014 16141072

[B17] Cheng J Kapranov P Drenkow J Dike S Brubaker S Patel S Long J Stern D Tammana H Helt G Sementchenko V Piccolboni A Bekiranov S Bailey DK Ganesh M Ghosh S Bell I Gerhard DS Gingeras TR Transcriptional maps of 10 human chromosomes at 5-nucleotide resolution Science 2005 308 1149 1154 10.1126/science.1108625 15790807

[B18] Geissmann T Chevalier C Cros MJ Boisset S Fechter P Noirot C Schrenzel J Francois P Vandenesch F Gaspin C Romby P A search for small noncoding RNAs in *Staphylococcus aureus *reveals a conserved sequence motif for regulation Nucleic Acids Res 2009 37 7239 7257 10.1093/nar/gkp668 PMC2790875 19786493

[B19] He H Wang J Liu T Liu XS Li T Wang Y Qian Z Zheng H Zhu X Wu T Shi B Deng W Zhou W Skogerbo G Chen R Mapping the *C. elegans *noncoding transcriptome with a whole-genome tiling microarray Genome Res 2007 17 1471 1477 10.1101/gr.6611807 PMC1987347 17785534

[B20] Jia H Osak M Bogu GK Stanton LW Johnson R Lipovich L Genome-wide computational identification and manual annotation of human long noncoding RNA genes RNA 2010 16 1478 1487 10.1261/rna.1951310 PMC2905748 20587619

[B21] Kapranov P Cheng J Dike S Nix DA Duttagupta R Willingham AT Stadler PF Hertel J Hackermuller J Hofacker IL Bell I Cheung E Drenkow J Dumais E Patel S Helt G Ganesh M Ghosh S Piccolboni A Sementchenko V Tammana H Gingeras TR RNA maps reveal new RNA classes and a possible function for pervasive transcription Science 2007 316 1484 1488 10.1126/science.1138341 17510325

[B22] Li Z Liu M Zhang L Zhang W Gao G Zhu Z Wei L Fan Q Long M Detection of intergenic non-coding RNAs expressed in the main developmental stages in *Drosophila melanogaster* Nucleic Acids Res 2009 37 4308 4314 10.1093/nar/gkp334 PMC2715228 19451167

[B23] Lu S Sun YH Amerson H Chiang VL MicroRNAs in loblolly pine (*Pinus taeda *L.) and their association with fusiform rust gall development Plant J 2007 51 1077 1098 10.1111/j.1365-313X.2007.03208.x 17635765

[B24] Tran TT Zhou F Marshburn S Stead M Kushner SR Xu Y *De novo *computational prediction of non-coding RNA genes in prokaryotic genomes Bioinformatics 2009 25 2897 2905 10.1093/bioinformatics/btp537 PMC2773258 19744996

[B25] Vercruysse M Fauvart M Cloots L Engelen K Thijs IM Marchal K Michiels J Genome-wide detection of predicted non-coding RNAs in *Rhizobium etli *expressed during free-living and host-associated growth using a high-resolution tiling array BMC Genomics 2010 11 53 10.1186/1471-2164-11-53 PMC2881028 20089193

[B26] Voss B Georg J Schon V Ude S Hess WR Biocomputational prediction of non-coding RNAs in model *cyanobacteria* BMC Genomics 2009 10 123 10.1186/1471-2164-10-123 PMC2662882 19309518

[B27] International Human Genome Sequencing Consortium Finishing the euchromatic sequence of the human genome Nature 2004 431 931 945 10.1038/nature03001 15496913

[B28] Faulkner GJ Kimura Y Daub CO Wani S Plessy C Irvine KM Schroder K Cloonan N Steptoe AL Lassmann T Waki K Hornig N Arakawa T Takahashi H Kawai J Forrest AR Suzuki H Hayashizaki Y Hume DA Orlando V Grimmond SM Carninci P The regulated retrotransposon transcriptome of mammalian cells Nat Genet 2009 41 563 571 10.1038/ng.368 19377475

[B29] Kapranov P Willingham AT Gingeras TR Genome-wide transcription and the implications for genomic organization Nat Rev Genet 2007 8 413 423 10.1038/nrg2083 17486121

[B30] Nagalakshmi U Wang Z Waern K Shou C Raha D Gerstein M Snyder M The transcriptional landscape of the yeast genome defined by RNA sequencing Science 2008 320 1344 1349 10.1126/science.1158441 PMC2951732 18451266

[B31] Wilhelm BT Marguerat S Watt S Schubert F Wood V Goodhead I Penkett CJ Rogers J Bahler J Dynamic repertoire of a eukaryotic transcriptome surveyed at single-nucleotide resolution Nature 2008 453 1239 1243 10.1038/nature07002 18488015

[B32] Wilusz JE Sunwoo H Spector DL Long noncoding RNAs: functional surprises from the RNA world Genes Dev 2009 23 1494 1504 10.1101/gad.1800909 PMC3152381 19571179

[B33] Griffiths-Jones S Annotating noncoding RNA genes Annu Rev Genomics Hum Genet 2007 8 279 298 10.1146/annurev.genom.8.080706.092419 17506659

[B34] Griffiths-Jones S Saini HK van Dongen S Enright AJ miRBase: tools for microRNA genomics Nucleic Acids Res 2008 36 D154 158 10.1093/nar/gkm952 PMC2238936 17991681

[B35] Bartel DP MicroRNAs: genomics, biogenesis, mechanism, and function Cell 2004 116 281 297 10.1016/s0092-8674(04)00045-5 14744438

[B36] Chen X Small RNAs and their roles in plant development Annu Rev Cell Dev Biol 2009 25 21 44 10.1146/annurev.cellbio.042308.113417 PMC5135726 19575669

[B37] Jones-Rhoades MW Bartel DP Bartel B MicroRNAs and their regulatory roles in plants Annu Rev Plant Biol 2006 57 19 53 10.1146/annurev.arplant.57.032905.105218 16669754

[B38] Voinnet O Origin, biogenesis, and activity of plant microRNAs Cell 2009 136 669 687 10.1016/j.cell.2009.01.046 19239888

[B39] Ben Amor B Wirth S Merchan F Laporte P d'Aubenton-Carafa Y Hirsch J Maizel A Mallory A Lucas A Deragon JM Vaucheret H Thermes C Crespi M Novel long non-protein coding RNAs involved in *Arabidopsis *differentiation and stress responses Genome Res 2009 19 57 69 10.1101/gr.080275.108 PMC2612962 18997003

[B40] Hirsch J Lefort V Vankersschaver M Boualem A Lucas A Thermes C d'Aubenton-Carafa Y Crespi M Characterization of 43 non-protein-coding mRNA genes in *Arabidopsis*, including the *MIR162a*-derived transcripts Plant Physiol 2006 140 1192 1204 10.1104/pp.105.073817 PMC1435803 16500993

[B41] Kurihara Y Matsui A Hanada K Kawashima M Ishida J Morosawa T Tanaka M Kaminuma E Mochizuki Y Matsushima A Toyoda T Shinozaki K Seki M Genome-wide suppression of aberrant mRNA-like noncoding RNAs by NMD in *Arabidopsis* Proc Natl Acad Sci USA 2009 106 2453 2458 10.1073/pnas.0808902106 PMC2650177 19181858

[B42] MacIntosh GC Wilkerson C Green PJ Identification and analysis of *Arabidopsis *expressed sequence tags characteristic of non-coding RNAs Plant Physiol 2001 127 765 776 PMC129250 11706161

[B43] Rymarquis LA Kastenmayer JP Huttenhofer AG Green PJ Diamonds in the rough: mRNA-like non-coding RNAs Trends Plant Sci 2008 13 329 334 10.1016/j.tplants.2008.02.009 18448381

[B44] Swiezewski S Liu F Magusin A Dean C Cold-induced silencing by long antisense transcripts of an *Arabidopsis *Polycomb target Nature 2009 462 799 802 10.1038/nature08618 20010688

[B45] Wen J Parker BJ Weiller GF *In silico *identification and characterization of mRNA-like noncoding transcripts in *Medicago truncatula* In Silico Biol 2007 7 485 505 18391239

[B46] Xin M Wang Y Yao Y Song N Hu Z Qin D Xie C Peng H Ni Z Sun Q Identification and characterization of wheat long non-protein coding RNAs responsive to powdery mildew infection and heat stress by using microarray analysis and SBS sequencing BMC Plant Biol 2011 11 61 10.1186/1471-2229-11-61 PMC3079642 21473757

[B47] Cho J Koo DH Nam YH Han CK Lim HT Bang JW Hur Y Isolation and characterization of cDNA clones expressed under male sex expression conditions in a monoecious cucumber plant (*Cucumis sativus *L.cv. Winter Long) Euphytica 2005 146 271 281

[B48] Kouchi H Takane K So RB Ladha JK Reddy PM Rice *ENOD40*: isolation and expression analysis in rice and transgenic soybean root nodules Plant J 1999 18 121 129 10.1046/j.1365-313x.1999.00432.x 10363365

[B49] Ma J Yan B Qu Y Qin F Yang Y Hao X Yu J Zhao Q Zhu D Ao G *Zm401*, a short-open reading-frame mRNA or noncoding RNA, is essential for tapetum and microspore development and can regulate the floret formation in maize J Cell Biochem 2008 105 136 146 10.1002/jcb.21807 18465785

[B50] Rohrig H John M Schmidt J Modification of soybean sucrose synthase by S-thiolation with ENOD40 peptide A Biochem Biophys Res Commun 2004 325 864 870 10.1016/j.bbrc.2004.10.100 15541370

[B51] Rohrig H Schmidt J Miklashevichs E Schell J John M Soybean *ENOD40 *encodes two peptides that bind to sucrose synthase Proc Natl Acad Sci USA 2002 99 1915 1920 10.1073/pnas.022664799 PMC122294 11842184

[B52] Sugiyama R Kazama Y Miyazawa Y Matsunaga S Kawano S *CCLS96.1*, a member of a multicopy gene family, may encode a non-coding RNA preferentially transcribed in reproductive organs of *Silene latifolia* DNA Res 2003 10 213 220 10.1093/dnares/10.5.213 14686583

[B53] Vleghels I Hontelez J Ribeiro A Fransz P Bisseling T Franssen H Expression of *ENOD40 *during tomato plant development Planta 2003 218 42 49 10.1007/s00425-003-1081-9 14508686

[B54] Burleigh SH Harrison MJ The down-regulation of *Mt4*-like genes by phosphate fertilization occurs systemically and involves phosphate translocation to the shoots Plant Physiol 1999 119 241 248 10.1104/pp.119.1.241 PMC32226 9880366

[B55] Heo JB Sung S Vernalization-mediated epigenetic silencing by a long intronic noncoding RNA Science 2011 331 76 79 10.1126/science.1197349 21127216

[B56] Liu C Muchhal US Raghothama KG Differential expression of *TPS11*, a phosphate starvation-induced gene in tomato Plant Mol Biol 1997 33 867 874 10.1023/a:1005729309569 9106510

[B57] Shin H Shin HS Chen R Harrison MJ Loss of *At4 *function impacts phosphate distribution between the roots and the shoots during phosphate starvation Plant J 2006 45 712 726 10.1111/j.1365-313X.2005.02629.x 16460506

[B58] Altschul SF Madden TL Schaffer AA Zhang J Zhang Z Miller W Lipman DJ Gapped BLAST and PSI-BLAST: a new generation of protein database search programs Nucleic Acids Res 1997 25 3389 3402 10.1093/nar/25.17.3389 PMC146917 9254694

[B59] Ashburner M Ball CA Blake JA Botstein D Butler H Cherry JM Davis AP Dolinski K Dwight SS Eppig JT Harris MA Hill DP Issel-Tarver L Kasarskis A Lewis S Matese JC Richardson JE Ringwald M Rubin GM Sherlock G Gene ontology: tool for the unification of biology. The Gene Ontology Consortium Nat Genet 2000 25 25 29 10.1038/75556 PMC3037419 10802651

[B60] Kanehisa M Goto S KEGG: kyoto encyclopedia of genes and genomes Nucleic Acids Res 2000 28 27 30 10.1093/nar/28.1.27 PMC102409 10592173

[B61] Kreis W Wink M Biochemistry of sterols, cardiac glycosides, brassinosteroids, phytoecdysteroids and steroid saponins Annual plant reviews 2010 40 2 Oxford: Garsington Road 304 363

[B62] Usai M Atzei AD Marchetti M Cardenolides content in wild Sardinian *Digitalis purpurea *L. populations Nat Prod Res 2007 21 798 804 10.1080/14786410701218291 17654283

[B63] Xiao B Zhang X Li Y Tang Z Yang S Mu Y Cui W Ao H Li K Identification, bioinformatic analysis and expression profiling of candidate mRNA-like non-coding RNAs in *Sus scrofa* J Genet Genomics 2009 36 695 702 10.1016/S1673-8527(08)60162-9 20129396

[B64] Inagaki S Numata K Kondo T Tomita M Yasuda K Kanai A Kageyama Y Identification and expression analysis of putative mRNA-like non-coding RNA in *Drosophila* Genes Cells 2005 10 1163 1173 10.1111/j.1365-2443.2005.00910.x 16324153

[B65] Szell M Bata-Csorgo Z Kemeny L The enigmatic world of mRNA-like ncRNAs: their role in human evolution and in human diseases Semin Cancer Biol 2008 18 141 148 10.1016/j.semcancer.2008.01.007 18282717

[B66] Zhang BH Pan XP Wang QL Cobb GP Anderson TA Identification and characterization of new plant microRNAs using EST analysis Cell Res 2005 15 336 360 10.1038/sj.cr.7290302 15916721

[B67] Kozomara A Griffiths-Jones S miRBase: integrating microRNA annotation and deep-sequencing data Nucleic Acids Res 2011 39 D152 157 10.1093/nar/gkq1027 PMC3013655 21037258

[B68] Zuker M Mfold web server for nucleic acid folding and hybridization prediction Nucleic Acids Res 2003 31 3406 3415 10.1093/nar/gkg595 PMC169194 12824337

[B69] Rhoades MW Reinhart BJ Lim LP Burge CB Bartel B Bartel DP Prediction of plant microRNA targets Cell 2002 110 513 520 10.1016/s0092-8674(02)00863-2 12202040

[B70] Dai X Zhuang Z Zhao PX Computational analysis of miRNA targets in plants: current status and challenges Briefings in bioinformatics 2011 12 115 121 10.1093/bib/bbq065 20858738

[B71] Zhang Y miRU: an automated plant miRNA target prediction server Nucleic Acids Res 2005 33 W701 704 10.1093/nar/gki383 PMC1160144 15980567

[B72] Lu S Sun YH Chiang VL Stress-responsive microRNAs in *Populus* Plant J 2008 55 131 151 10.1111/j.1365-313X.2008.03497.x 18363789

[B73] Lu S Sun YH Shi R Clark C Li L Chiang VL Novel and mechanical stress-responsive microRNAs in *Populus trichocarpa *that are absent from *Arabidopsis* Plant Cell 2005 17 2186 2203 10.1105/tpc.105.033456 PMC1182482 15994906

[B74] Loeffler C Berger S Guy A Durand T Bringmann G Dreyer M von Rad U Durner J Mueller MJ B1-phytoprostanes trigger plant defense and detoxification responses Plant Physiol 2005 137 328 340 10.1104/pp.104.051714 PMC548863 15618427

[B75] Reddy VS Ali GS Reddy AS Genes encoding calmodulin-binding proteins in the *Arabidopsis *genome J Biol Chem 2002 277 9840 9852 10.1074/jbc.M111626200 11782485

[B76] Soitamo AJ Piippo M Allahverdiyeva Y Battchikova N Aro EM Light has a specific role in modulating *Arabidopsis *gene expression at low temperature BMC Plant Biol 2008 8 13 10.1186/1471-2229-8-13 PMC2253524 18230142

[B77] Gong Z Dong CH Lee H Zhu J Xiong L Gong D Stevenson B Zhu JK A DEAD box RNA helicase is essential for mRNA export and important for development and stress responses in *Arabidopsis* Plant Cell 2005 17 256 267 10.1105/tpc.104.027557 PMC544503 15598798

[B78] Kant P Kant S Gordon M Shaked R Barak S STRESS RESPONSE SUPPRESSOR1 and STRESS RESPONSE SUPPRESSOR2, two DEAD-box RNA helicases that attenuate *Arabidopsis *responses to multiple abiotic stresses Plant Physiol 2007 145 814 830 10.1104/pp.107.099895 PMC2048787 17556511

[B79] Kim JS Kim KA Oh TR Park CM Kang H Functional characterization of DEAD-box RNA helicases in *Arabidopsis thaliana *under abiotic stress conditions Plant Cell Physiol 2008 49 1563 1571 10.1093/pcp/pcn125 18725370

[B80] Li D Liu H Zhang H Wang X Song F OsBIRH1, a DEAD-box RNA helicase with functions in modulating defence responses against pathogen infection and oxidative stress J Exp Bot 2008 59 2133 2146 10.1093/jxb/ern072 PMC2413282 18441339

[B81] Ascencio-Ibanez JT Sozzani R Lee TJ Chu TM Wolfinger RD Cella R Hanley-Bowdoin L Global analysis of *Arabidopsis *gene expression uncovers a complex array of changes impacting pathogen response and cell cycle during geminivirus infection Plant Physiol 2008 148 436 454 10.1104/pp.108.121038 PMC2528102 18650403

[B82] Yabe N Takahashi T Komeda Y Analysis of tissue-specific expression of *Arabidopsis thaliana *HSP90-family gene *HSP81* Plant Cell Physiol 1994 35 1207 1219 10.1093/oxfordjournals.pcp.a078715 7697294

[B83] He S Liu C Skogerbo G Zhao H Wang J Liu T Bai B Zhao Y Chen R NONCODE v2.0: decoding the non-coding Nucleic Acids Res 2008 36 D170 172 10.1093/nar/gkm1011 PMC2238973 18000000

[B84] Numata K Kanai A Saito R Kondo S Adachi J Wilming LG Hume DA Hayashizaki Y Tomita M Identification of putative noncoding RNAs among the RIKEN mouse full-length cDNA collection Genome Res 2003 13 1301 1306 10.1101/gr.1011603 PMC403720 12819127

[B85] Taylor CB Green PJ Identification and characterization of genes with unstable transcripts (GUTs) in tobacco Plant Mol Biol 1995 28 27 38 10.1007/BF00042035 7787185

[B86] van Hoof A Kastenmayer JP Taylor CB Green PJ *GUT15 *cDNAs from tobacco (Accession No. U84972) and *Arabidopsis *(Accession No. U84973) correspond to transcripts with unusual metabolism and a short conserved ORF (PGR97-048) Plant Physiol 1997 113 1004

[B87] Vandesompele J De Preter K Pattyn F Poppe B Van Roy N De Paepe A Speleman F Accurate normalization of real-time quantitative RT-PCR data by geometric averaging of multiple internal control genes Genome Biol 2002 3 RESEARCH0034 10.1186/gb-2002-3-7-research0034 PMC126239 12184808

[B88] Gil MJ Coego A Mauch-Mani B Jorda L Vera P The *Arabidopsis csb3 *mutant reveals a regulatory link between salicylic acid-mediated disease resistance and the methyl-erythritol 4-phosphate pathway Plant J 2005 44 155 166 10.1111/j.1365-313X.2005.02517.x 16167903

[B89] Kim SM Kim SU Characterization of 1-hydroxy-2-methyl-2-(E)-butenyl-4-diphosphate synthase (HDS) gene from *Ginkgo biloba* Mol Biol Rep 2010 37 973 979 10.1007/s11033-009-9771-4 19728152

[B90] Querol J Campos N Imperial S Boronat A Rodriguez-Concepcion M Functional analysis of the *Arabidopsis thalian*a GCPE protein involved in plastid isoprenoid biosynthesis FEBS Lett 2002 514 343 346 10.1016/s0014-5793(02)02402-x 11943178

[B91] Sando T Takeno S Watanabe N Okumoto H Kuzuyama T Yamashita A Hattori M Ogasawara N Fukusaki E Kobayashi A Cloning and characterization of the 2-C-methyl-D-erythritol 4-phosphate (MEP) pathway genes of a natural-rubber producing plant, *Hevea brasiliensis* Biosci Biotechnol Biochem 2008 72 2903 2917 10.1271/bbb.80387 18997428

[B92] Hirooka K Bamba T Fukusaki E Kobayashi A Cloning and kinetic characterization of *Arabidopsis thaliana *solanesyl diphosphate synthase Biochem J 2003 370 679 686 10.1042/BJ20021311 PMC1223189 12437513

[B93] Khachane AN Harrison PM Mining mammalian transcript data for functional long non-coding RNAs PLoS One 2010 5 e10316 10.1371/journal.pone.0010316 PMC2859052 20428234

[B94] Pagano A Castelnuovo M Tortelli F Ferrari R Dieci G Cancedda R New small nuclear RNA gene-like transcriptional units as sources of regulatory transcripts PLoS Genet 2007 3 e1 10.1371/journal.pgen.0030001 PMC1790723 17274687

[B95] Royo H Cavaille J Non-coding RNAs in imprinted gene clusters Biol Cell 2008 100 149 166 10.1042/BC20070126 18271756

[B96] Xie DY Jackson LA Cooper JD Ferreira D Paiva NL Molecular and biochemical analysis of two cDNA clones encoding dihydroflavonol-4-reductase from *Medicago truncatula* Plant Physiol 2004 134 979 994 10.1104/pp.103.030221 PMC389921 14976232

[B97] Bartley GE Scolnik PA Plant carotenoids: pigments for photoprotection, visual attraction, and human health Plant Cell 1995 7 1027 1038 10.1105/tpc.7.7.1027 PMC160905 7640523

[B98] Facchini PJ Huber-Allanach KL Tari LW Plant aromatic L-amino acid decarboxylases: evolution, biochemistry, regulation, and metabolic engineering applications Phytochemistry 2000 54 121 138 10.1016/s0031-9422(00)00050-9 10872203

[B99] Lehmann T Pollmann S Gene expression and characterization of a stress-induced tyrosine decarboxylase from *Arabidopsis thaliana* FEBS Lett 2009 583 1895 1900 10.1016/j.febslet.2009.05.017 19450582

[B100] Fahlgren N Howell MD Kasschau KD Chapman EJ Sullivan CM Cumbie JS Givan SA Law TF Grant SR Dangl JL Carrington JC High-throughput sequencing of *Arabidopsis *microRNAs: evidence for frequent birth and death of *MIRNA *genes PLoS One 2007 2 e219 10.1371/journal.pone.0000219 PMC1790633 17299599

[B101] Gutierrez L Bussell JD Pacurar DI Schwambach J Pacurar M Bellini C Phenotypic plasticity of adventitious rooting in Arabidopsis is controlled by complex regulation of AUXIN RESPONSE FACTOR transcripts and microRNA abundance Plant Cell 2009 21 3119 3132 10.1105/tpc.108.064758 PMC2782293 19820192

[B102] Hardtke CS Berleth T The *Arabidopsis *gene *MONOPTEROS *encodes a transcription factor mediating embryo axis formation and vascular development EMBO J 1998 17 1405 1411 10.1093/emboj/17.5.1405 PMC1170488 9482737

[B103] Harper RM Stowe-Evans EL Luesse DR Muto H Tatematsu K Watahiki MK Yamamoto K Liscum E The *NPH4 *locus encodes the auxin response factor ARF7, a conditional regulator of differential growth in aerial *Arabidopsis *tissue Plant Cell 2000 12 757 770 10.1105/tpc.12.5.757 PMC139925 10810148

[B104] Schruff MC Spielman M Tiwari S Adams S Fenby N Scott RJ The *AUXIN RESPONSE FACTOR 2 *gene of *Arabidopsis *links auxin signalling, cell division, and the size of seeds and other organs Development 2006 133 251 261 10.1242/dev.02194 16339187

[B105] Sessions A Nemhauser JL McColl A Roe JL Feldmann KA Zambryski PC ETTIN patterns the *Arabidopsis *floral meristem and reproductive organs Development 1997 124 4481 4491 10.1242/dev.124.22.4481 9409666

[B106] Tatematsu K Kumagai S Muto H Sato A Watahiki MK Harper RM Liscum E Yamamoto KT *MASSUGU2 *encodes Aux/IAA19, an auxin-regulated protein that functions together with the transcriptional activator NPH4/ARF7 to regulate differential growth responses of hypocotyl and formation of lateral roots in *Arabidopsis thaliana* Plant Cell 2004 16 379 393 10.1105/tpc.018630 PMC341911 14729917

[B107] Varaud E Brioudes F Szecsi J Leroux J Brown S Perrot-Rechenmann C Bendahmane M *AUXIN RESPONSE FACTOR8 *regulates *Arabidopsis *petal growth by interacting with the bHLH transcription factor BIGPETALp Plant Cell 2011 23 973 983 10.1105/tpc.110.081653 PMC3082276 21421811

[B108] Yant L Mathieu J Dinh TT Ott F Lanz C Wollmann H Chen X Schmid M Orchestration of the floral transition and floral development in *Arabidopsis *by the bifunctional transcription factor *APETALA2* Plant Cell 2010 22 2156 2170 10.1105/tpc.110.075606 PMC2929098 20675573

[B109] Liu D Song Y Chen Z Yu D Ectopic expression of miR396 suppresses GRF target gene expression and alters leaf growth in *Arabidopsis* Physiol Plant 2009 136 223 236 10.1111/j.1399-3054.2009.01229.x 19453503

[B110] Rodriguez RE Mecchia MA Debernardi JM Schommer C Weigel D Palatnik JF Control of cell proliferation in *Arabidopsis thaliana *by microRNA miR396 Development 2010 137 103 112 10.1242/dev.043067 PMC2796936 20023165

[B111] Campalans A Kondorosi A Crespi M *Enod40*, a short open reading frame-containing mRNA, induces cytoplasmic localization of a nuclear RNA binding protein in *Medicago truncatula* Plant Cell 2004 16 1047 1059 10.1105/tpc.019406 PMC412876 15037734

[B112] Dai X Yu J Ma J Ao G Zhao Q Overexpression of *Zm401*, an mRNA-like RNA, has distinct effects on pollen development in maize Plant Growth Regul 2007 52 229 139

[B113] Franco-Zorrilla JM Valli A Todesco M Mateos I Puga MI Rubio-Somoza I Leyva A Weigel D Garcia JA Paz-Ares J Target mimicry provides a new mechanism for regulation of microRNA activity Nat Genet 2007 39 1033 1037 10.1038/ng2079 17643101

[B114] Kim DH Zografos BR Sung S Mechanisms underlying vernalization-mediated *VIN3 *induction in *Arabidopsis* Plant Signal Behav 2010 5 10.4161/psb.5.11.13465 PMC3115254 21051939

[B115] Szymanski M Barciszewska MZ Zywicki M Barciszewski J Noncoding RNA transcripts J Appl Genet 2003 44 1 19 12590177

[B116] Carthew RW Sontheimer EJ Origins and mechanisms of miRNAs and siRNAs Cell 2009 136 642 655 10.1016/j.cell.2009.01.035 PMC2675692 19239886

[B117] Chen X Small RNAs - secrets and surprises of the genome Plant J 2010 61 941 958 10.1111/j.1365-313X.2009.04089.x PMC3062250 20409269

[B118] Vazquez F Legrand S Windels D The biosynthetic pathways and biological scopes of plant small RNAs Trends Plant Sci 2010 15 337 345 10.1016/j.tplants.2010.04.001 20427224

[B119] Duret L Chureau C Samain S Weissenbach J Avner P The *Xist *RNA gene evolved in *eutherians *by pseudogenization of a protein-coding gene Science 2006 312 1653 1655 10.1126/science.1126316 16778056

[B120] Elisaphenko EA Kolesnikov NN Shevchenko AI Rogozin IB Nesterova TB Brockdorff N Zakian SM A dual origin of the *Xist *gene from a protein-coding gene and a set of transposable elements PLoS One 2008 3 e2521 10.1371/journal.pone.0002521 PMC2430539 18575625

[B121] Napoli C Lemieux C Jorgensen R Introduction of a chimeric chalcone synthase gene into *Petunia *results in reversible co-suppression of homologous genes in trans Plant Cell 1990 2 279 289 10.1105/tpc.2.4.279 PMC159885 12354959

[B122] Smith CJ Watson CF Bird CR Ray J Schuch W Grierson D Expression of a truncated tomato polygalacturonase gene inhibits expression of the endogenous gene in transgenic plants Mol Gen Genet 1990 224 477 481 10.1007/BF00262443 2266949

[B123] van der Krol AR Mur LA Beld M Mol JN Stuitje AR Flavonoid genes in petunia: addition of a limited number of gene copies may lead to a suppression of gene expression The Plant cell 1990 2 291 299 10.1105/tpc.2.4.291 PMC159886 2152117

[B124] Lottaz C Iseli C Jongeneel CV Bucher P Modeling sequencing errors by combining Hidden Markov models Bioinformatics 2003 19 Suppl 2 ii103 112 10.1093/bioinformatics/btg1067 14534179

[B125] Gardner PP Daub J Tate J Moore BL Osuch IH Griffiths-Jones S Finn RD Nawrocki EP Kolbe DL Eddy SR Bateman A Rfam: Wikipedia, clans and the "decimal" release Nucleic Acids Res 2011 39 D141 145 10.1093/nar/gkq1129 PMC3013711 21062808

[B126] Lelandais-Briere C Naya L Sallet E Calenge F Frugier F Hartmann C Gouzy J Crespi M Genome-wide *Medicago truncatula *small RNA analysis revealed novel microRNAs and isoforms differentially regulated in roots and nodules Plant Cell 2009 21 2780 2796 10.1105/tpc.109.068130 PMC2768930 19767456

[B127] Zhang BH Pan XP Cox SB Cobb GP Anderson TA Evidence that miRNAs are different from other RNAs Cell Mol Life Sci 2006 63 246 254 10.1007/s00018-005-5467-7 PMC11136112 16395542

[B128] Livak KJ Schmittgen TD Analysis of relative gene expression data using real-time quantitative PCR and the 2(-Delta Delta C(T)) method Methods 2001 25 402 408 10.1006/meth.2001.1262 11846609

