# Supplementary material for: Comprehensive transcriptome analysis reveals novel genes involved in cardiac glycoside biosynthesis and mlncRNAs associated with secondary metabolism and stress response in Digitalis purpurea
Source: BMC Genomics. 2012 Jan 10;13:15. doi: 10.1186/1471-2164-13-15 (PMC3269984; doi:10.1186/1471-2164-13-15)
Supplement: Additional file 1 — Unigenes probably involved in cardiac glycoside biosynthesis. Complete set of unigenes probably involved in cardiac glycoside biosynthesis. [file 1471-2164-13-15-S1.PDF]

Additional file 1. Unigenes probably involved in cardiac glycoside biosynthesis

Enzyme name	EC number	Unigene ID
**Terpenoid backbone biosynthesis**		
1-deoxy-D-xylulose-5-phosphate synthase (DXS)	EC:2.2.1.7	FXAT9O005F8E8Y |FXAT9O005FPLOH | FXAT9O005FSLZ0| FXAT9O005F03ZC| JO464370|FXAT9O005F9AQ7
1-deoxy-D-xylulose-5-phosphate reductoisomerase (DXR)	EC:1.1.1.267	JO462912|FXAT9O005F3VI7
4-diphosphocytidyl-2-C-methyl-D-erythritol kinase (CMK)	EC:2.7.1.148	JO466008
2-C-methyl-D-erythritol 2,4-cyclodiphosphate synthase (MDS)	EC:4.6.1.12	JO462979
4-hydroxy-3-methylbut-2-en-1-yl diphosphate synthase (HDS)	EC:1.17.7.1	JO462517 | JO462557|JO463639
4-hydroxy-3-methylbut-2-enyl diphosphate reductase (HDR)	EC:1.17.1.2	JO461294
Hydroxymethylglutaryl-CoA reductase (HMGR)	EC:1.1.1.34	JO466235 | JO461014
mevalonate kinase (MK)	EC:2.7.1.36	JO463177
Phosphomevalonate kinase (PMK)	EC:2.7.4.2	FXAT9O005FKUVA
Diphosphomevalonate decarboxylase (MPD)	EC:4.1.1.33	JO461137
Isopentenyl-diphosphate delta-isomerase (IDI)	EC:5.3.3.2	JO464842 | FXAT9O005GBLG1
Geranylgeranyl pyrophosphate synthase 1 (GGPPS1)	EC:2.5.1.1/ EC:2.5.1.10	FXAT9O005GDF6O
Farnesyl diphosphate synthase 1(FDPS1)	EC:2.5.1.1/ EC:2.5.1.10	JO461677| JO462469
**Steroid biosynthesis**		
Squalene synthase 1(SQS1)	EC:2.5.1.21	JO462207
Squalene monooxygenase(SMO)	EC:1.14.99.7	FXAT9O005F0UT8|JO464555|JO466180 |FXAT9O005F1SB9| FXAT9O005FOPQS
Cycloartenol synthase 1(CAS1)	EC:5.4.99.8	FXAT9O005F16SH
Sterol 24-C-methyltransferase (SMT1)	EC:2.1.1.4	JO467498
Cyclopropyl isomerase(CPI1)	EC:5.5.1.9	FXAT9O005FYYXO| FXAT9O005GBO91
Cytochrome P450, family 51(CYP51G1)	EC:1.14.13.7 0	JO463413|JO465891
Delta14-sterol reductase(FK)	EC:1.3.1.70	FXAT9O005FXQWD
Cholestenol delta-isomerase(HYD1)	EC:5.3.3.5	JO461562
Sterol methyltransferase 2(SMT2)	EC:2.1.1.143	JO460295| FXAT9O005F9U7K
C-5 sterol desaturase(STE1)	EC:1.14.2.6	JO466129
Sterol delta7 reductase(DWF5)	EC:1.3.1.21	JO461977
Delta24-sterol reductase (DWF1)	EC:1.3.1.72	JO460872 | FXAT9O005F0AGU

**Cardenolide biosynthesis**	
3-beta-hydroxysteroid dehydrogenase(3βHSD)	JO460585
Progesterone 5β-reductase(5βPOR)	FXAT9O005F10IY | JO462018
Monooxygenase	FXAT9O005F2C30| FXAT9O005GAGDF| FXAT9O005FORR9| FXAT9O005FZSBI| JO462256| JO464073|JO464188| JO464422| FXAT9O005GEXH1| FXAT9O005F6Z8S| FXAT9O005FRWY9| FXAT9O005FPBG0| JO466068|JO466707| JO460379| JO460249| FXAT9O005FPXJF| FXAT9O005FROM0| FXAT9O005GDDZB|FXAT9O005FWWB4| JO460673| JO463349|JO467493| JO463457| FXAT9O005FMWPM|FXAT9O005GE7YL| FXAT9O005F475A| FXAT9O005F7PYW| FXAT9O005F9RBA| FXAT9O005GCP44| FXAT9O005GFJSQ| FXAT9O005F4FBA| FXAT9O005FVK0K|FXAT9O005F2G1C JO462129| JO466049|
Cardenolide 16-O-glucohydrolase(CGHⅠ)	JO460189
Glycosyltransferase/ UDP- glycosyltransferase (GT / UGT)	JO465897| JO462628|JO461498| JO466233| FXAT9O005FP2YY| FXAT9O005GD9KP| FXAT9O005GGUB5| JO464656|JO465866| JO463730|FXAT9O005FY5DW| JO464291| FXAT9O005FTSKG|FXAT9O005FMHWC|FXAT9O005FQG6D| FXAT9O005FZP2W| FXAT9O005F4MVY| FXAT9O005GE0LB| JO465038|JO467019| FXAT9O005GE6RZ| FXAT9O005GB5WU| XAT9O005GA8E3| FXAT9O005FOR2I| FXAT9O005GD7R9| FXAT9O005GEDU6| JO464168| JO462065| JO464881| FXAT9O005FWS9V| JO461371| FXAT9O005GD23X|FXAT9O005F0A0B| JO463637|JO464407| JO460892| JO461094| JO464094|FXAT9O005FZVBK| FXAT9O005GEJ1G|FXAT9O005F8I2I| FXAT9O005FZYSS|JO460827|JO463892|FXAT9O005FMCAO| FXAT9O005GDDC3| JO464412| FXAT9O005FVBIM| FXAT9O005FVEF9|JO466031| JO464632| JO467255| JO467324| FXAT9O005FXQXB FXAT9O005F5EZU| FXAT9O005GC4ZI|
