# Supplementary material for: Comprehensive transcriptome analysis reveals novel genes involved in cardiac glycoside biosynthesis and mlncRNAs associated with secondary metabolism and stress response in Digitalis purpurea
Source: BMC Genomics. 2012 Jan 10;13:15. doi: 10.1186/1471-2164-13-15 (PMC3269984; doi:10.1186/1471-2164-13-15)
Supplement: Additional file 2 — The 25 most abundant unigenes in D. purpurea. Complete set of the 25 most abundant unigenes in D. purpurea. [file 1471-2164-13-15-S2.PDF]

Additional file 2. The 25 most abundant unigenes in *D. purpurea*.

Unigene ID	Reads	Accession number[a]	Annotation
JO460546	268	emb|CAH58646.1|	Aminocyclopropan-1-carboxylate oxidase
JO460074	266	gb|EEF30385.1|	Xyloglucan endotransglucosylase/hydrolase protein 9 precursor
JO461281	238	gb|EEE87009.1|	Predicted protein
JO460516	202	gb|ACD03218.1|	Xyloglucan endotransglucosylase/hydrolase 8
JO460075	150	ref|XP_002284968.1|	Hypothetical protein
JO460497	144	gb|EEF38130.1|	Ribulose bisphosphate carboxylase/oxygenase activase 1, chloroplast precursor
JO460083	143	sp|Q42679|DCAM_CATRO	S-adenosylmethionine decarboxylase proenzyme
JO460114	136	gb|ABB89735.1|	ERD15
JO467328	133	gb|ACD03228.1|	Xyloglucan endotransglucosylase/hydrolase 4
JO460040	129	no hit	No hit
JO467390	128	gb|ABK92496.1|	Unknown
JO460293	127	gb|ACD03228.1|	Xyloglucan endotransglucosylase/hydrolase 4
JO460110	119	gb|EEE87558.1|	Predicted protein
JO460216	118	gb|AAC25984.1|	Beta-galactosidase
JO467367	116	gb|EEF36515.1|	Conserved hypothetical protein
JO460506	111	gb|EEF38130.1|	Ribulose bisphosphate carboxylase/oxygenase activase 1, chloroplast precursor,
JO460039	109	gb|ABW74471.1|	Auxin-repressed protein
JO461875	107	emb|CAA04653.1|	Major intrinsic protein PIPB
JO460712	104	gb|AAF70822.1|AF154421_1	Beta-galactosidase
JO460015	102	no hit	No hit
JO467348	101	gb|AAA33866.1|	Ribulose 1,5-bisphosphate carboxylase small subunit
JO460189	97	emb|CAB38854.2|	Cardenolide 16-O-glucohydrolase
Contig08285	97	gb|ABK95255.1|	Unknown
Contig00124	95	no hit	No hit
JO461862	94	ref|XP_002270896.1|	Hypothetical protein

[a] The best hit in the NCBI non-redundant (Nr) protein database
