# Supplementary material for: Comprehensive transcriptome analysis reveals novel genes involved in cardiac glycoside biosynthesis and mlncRNAs associated with secondary metabolism and stress response in Digitalis purpurea
Source: BMC Genomics. 2012 Jan 10;13:15. doi: 10.1186/1471-2164-13-15 (PMC3269984; doi:10.1186/1471-2164-13-15)
Supplement: Additional file 3 — Housekeeping npcRNAs identified. Complete set of the housekeeping npcRNAs identified. [file 1471-2164-13-15-S3.PDF]

**Additional file 3.** Housekeeping npcRNAs identified.

| Unigene ID | npcRNA name | npcRNA ID in the Rfam database |
|---|---|---|
| JO460492 | snoR1 | RF00345 |
| JO460492 | SNORD25 | RF00054 |
| JO460492 | snoR77 | RF01230 |
| JO460493 | snoZ43 | RF00330 |
| JO460493 | SNORD27 | RF00086 |
| JO460713 | SNORD14 | RF00016 |
| JO462644 | snoR41 | RF00205 |
| JO463831 | tRNA | RF00005 |
| JO464257 | U3 | RF00012 |
| JO466664 | snoU36a | RF01302 |
| FXAT9O005F1RB3 | snoZ101 | RF00358 |
| FXAT9O005F1RB3 | snoR14 | RF01280 |
| FXAT9O005FVGZC | snoZ43 | RF00330 |
| FXAT9O005FP343 | SNORD36 | RF00049 |
| FXAT9O005FTAWV | snoZ107_R87 | RF00360 |
| FXAT9O005FP9JT | snoR12 | RF00204 |
| FXAT9O005FP9JT | SNORD24 | RF00069 |
| FXAT9O005GATDV | snoZ221_snoR21b | RF00300 |
| FXAT9O005FY1HK | U2 | RF00004 |
| FXAT9O005FRC8C | snoR72Y | RF01217 |
| FXAT9O005FRC8C | snoZ122 | RF00343 |
| FXAT9O005FZQ5N | snoZ103 | RF00149 |
| FXAT9O005F5AS3 | SNORD96 | RF00055 |
| FXAT9O005FXTLJ | snoR24 | RF00132 |
| FXAT9O005F6077 | snoZ101 | RF00358 |
| FXAT9O005F6077 | snoR14 | RF01280 |
| FXAT9O005FN5FP | snoR44_J54 | RF00357 |
