# Supplementary material for: Comprehensive transcriptome analysis reveals novel genes involved in cardiac glycoside biosynthesis and mlncRNAs associated with secondary metabolism and stress response in Digitalis purpurea
Source: BMC Genomics. 2012 Jan 10;13:15. doi: 10.1186/1471-2164-13-15 (PMC3269984; doi:10.1186/1471-2164-13-15)
Supplement: Additional file 4 — D. purpurea mlncRNA families. Complete set of the D. purpurea mlncRNA families. [file 1471-2164-13-15-S4.PDF]

Additional file 4. *D. purpurea* mlncRNA families.

Family No.	Unigene ID							
1	FXAT9O005F2XOS	FXAT9O005FNNS5	FXAT9O005F63SF	FXAT9O005FXA3E	FXAT9O005FW3GF	FXAT9O005FXDAT	FXAT9O005FPWUE	FXAT9O005F32MA
2	JO466327	FXAT9O005FNR9U	FXAT9O005FRATZ	FXAT9O005F3VCL	FXAT9O005FRLA9	FXAT9O005FRPH5	FXAT9O005FMFYC	
3	JO461863	JO466083	FXAT9O005FLP32	FXAT9O005GBOSL	FXAT9O005F4QZH	FXAT9O005FNZ6N		
4	JO467312	FXAT9O005FOL4X	FXAT9O005FVKXA	FXAT9O005F9YU3	FXAT9O005FRSLG	FXAT9O005FWNNN		
5	FXAT9O005F2ER6	FXAT9O005FNYUM	FXAT9O005F3FSZ	FXAT9O005FPZTR	FXAT9O005GCPNT	FXAT9O005F55I7		
6	JO460015	JO463036	FXAT9O005GBZG4	FXAT9O005FR36L				
7	JO460006	FXAT9O005FQM9N	FXAT9O005FZD0A	FXAT9O005F88IL				
8	FXAT9O005FVZ2Q	FXAT9O005GBFA7	FXAT9O005GALC7	FXAT9O005FUP4B				
9	FXAT9O005FW6CA	FXAT9O005GDFOI	FXAT9O005GA1ON	FXAT9O005F531X				
10	FXAT9O005F24GR	FXAT9O005F70YH	FXAT9O005GBNXH	FXAT9O005FVRIU				
11	FXAT9O005F2TX8	FXAT9O005GC5H4	FXAT9O005F62GT					
12	FXAT9O005F9EBN	FXAT9O005GC9X2	FXAT9O005GC0U2					
13	FXAT9O005FP5KL	FXAT9O005F0AUQ	FXAT9O005FTEQ0					
14	FXAT9O005FTJU1	FXAT9O005FVGZC	FXAT9O005FTW0R					
15	FXAT9O005FWILV	FXAT9O005GBSC8	FXAT9O005FV7O2					
16	FXAT9O005FXDND	FXAT9O005FOJI4	FXAT9O005GC2PW					
17	FXAT9O005FXOV6	FXAT9O005FX6QF	FXAT9O005F2XNC					
18	JO460014	JO460204	FXAT9O005F35KO					
19	JO460102	JO461962	FXAT9O005F12G8					
20	JO460224	FXAT9O005F6AIL	FXAT9O005F0XYW					
21	JO460538	JO467387	FXAT9O005GDT2E					
22	JO460800	FXAT9O005FMK43	FXAT9O005FR9LW					
23	JO460037	JO460178						
24	JO460239	JO460241						
25	JO460509	JO460521						
26	JO460980	JO460985						
27	JO461237	JO467341						
28	JO461652	JO461662						
29	JO461880	FXAT9O005FUJUB						
30	JO461893	JO461899						
31	JO462108	JO463555						
32	JO462445	FXAT9O005GC0AN						
33	JO462502	FXAT9O005GAKOU						
34	JO462746	FXAT9O005F185K						
35	JO462822	JO462823						
36	JO462845	JO462857						
37	JO463019	FXAT9O005FOR2W						
38	JO463149	JO463152						
39	JO463280	FXAT9O005F540N						
40	JO463570	FXAT9O005F8URZ						
41	JO463951	JO467246						

42	JO465735	JO466108						
43	JO465969	FXAT9O005GEMCP						
44	JO466357	FXAT9O005FVI5G						
45	JO466391	FXAT9O005FLV80						
46	JO466435	JO466437						
47	JO466494	JO462321						
48	JO466666	FXAT9O005FO1AQ						
49	JO466767	FXAT9O005FVLLO						
50	JO466820	FXAT9O005F8NA4						
51	JO466844	FXAT9O005F13CO						
52	JO466884	FXAT9O005FSNUQ						
53	JO467063	FXAT9O005FQZ6G						
54	FXAT9O005F00AI	FXAT9O005FWP1W						
55	FXAT9O005F05QT	FXAT9O005F6N6G						
56	FXAT9O005F08R4	FXAT9O005FPDUT						
57	FXAT9O005F09T4	FXAT9O005GCU5E						
58	FXAT9O005F0B2G	FXAT9O005FP2QQ						
59	FXAT9O005F0CDL	FXAT9O005FTVZT						
60	FXAT9O005F0CY9	FXAT9O005F5I0M						
61	FXAT9O005F0GRZ	FXAT9O005FRKR8						
62	FXAT9O005F0MPF	FXAT9O005FX2B4						
63	FXAT9O005F0VIA	FXAT9O005FSVWD						
64	FXAT9O005F10EG	FXAT9O005F4PBD						
65	FXAT9O005F12TP	FXAT9O005FLYRE						
66	FXAT9O005F1F8U	FXAT9O005F5XDF						
67	FXAT9O005F1G8B	FXAT9O005GBKV8						
68	FXAT9O005F1IJI	FXAT9O005GB37P						
69	FXAT9O005F1O2F	FXAT9O005FVPPQ						
70	FXAT9O005F1V9V	FXAT9O005GDU67						
71	FXAT9O005F1ZKP	FXAT9O005FVP0S						
72	FXAT9O005F24A3	FXAT9O005FXENV						
73	FXAT9O005F28DQ	FXAT9O005GD5H0						
74	FXAT9O005F2AKW	FXAT9O005FV0X4						
75	FXAT9O005F2C1Z	FXAT9O005F7BAY						
76	FXAT9O005F2KX9	FXAT9O005FVX2W						
77	FXAT9O005F2M3Z	FXAT9O005F7D1Y						
78	FXAT9O005F2UDI	FXAT9O005FMTJV						
79	FXAT9O005F31AZ	FXAT9O005FTUE8						
80	FXAT9O005F3B2E	FXAT9O005F72AW						
81	FXAT9O005F3LHQ	FXAT9O005GCWIG						
82	FXAT9O005F3QV5	FXAT9O005FLSVQ						
83	FXAT9O005F3RPX	FXAT9O005F8LB6						
84	FXAT9O005F4GK6	FXAT9O005F9WJ6						

85	FXAT9O005F4NPC	FXAT9O005F6Z7G						
86	FXAT9O005F5AS3	FXAT9O005FWOQ2						
87	FXAT9O005F5PNI	FXAT9O005FNUX7						
88	FXAT9O005F5WR6	FXAT9O005FQHRX						
89	FXAT9O005F63O8	FXAT9O005FNL28						
90	FXAT9O005F6GG9	FXAT9O005FS0G3						
91	FXAT9O005F6J14	FXAT9O005F7YZH						
92	FXAT9O005F6NRN	FXAT9O005GDUQA						
93	FXAT9O005F6QJZ	FXAT9O005FZKAN						
94	FXAT9O005F6SYW	FXAT9O005FQGH9						
95	FXAT9O005F78Y0	FXAT9O005FRKQJ						
96	FXAT9O005F7OI5	FXAT9O005FLHI7						
97	FXAT9O005F7OQJ	FXAT9O005FT10Z						
98	FXAT9O005F7V84	FXAT9O005FUUWW						
99	FXAT9O005F7WNT	FXAT9O005FWD2M						
100	FXAT9O005F875E	FXAT9O005FOMHH						
101	FXAT9O005F88MH	FXAT9O005FS0XY						
102	FXAT9O005F8DDZ	FXAT9O005GERZE						
103	FXAT9O005F8LI9	FXAT9O005FNN9J						
104	FXAT9O005F8SIN	FXAT9O005FWA3N						
105	FXAT9O005F8SMK	FXAT9O005FYS4V						
106	FXAT9O005F99FZ	FXAT9O005FUDB7						
107	FXAT9O005FK07L	FXAT9O005FVTXT						
108	FXAT9O005FL21T	FXAT9O005GG393						
109	FXAT9O005FLNAB	FXAT9O005FXFUZ						
110	FXAT9O005FLV6A	FXAT9O005FX1MI						
111	FXAT9O005FLVP8	FXAT9O005FXOLJ						
112	FXAT9O005FLW3Q	FXAT9O005FTDTX						
113	FXAT9O005FLYJ0	FXAT9O005FX5T3						
114	FXAT9O005FM07B	FXAT9O005GFVTV						
115	FXAT9O005FMCD0	FXAT9O005FTPYO						
116	FXAT9O005FMMDL	FXAT9O005FZSI2						
117	FXAT9O005FMMDT	FXAT9O005FMVL7						
118	FXAT9O005FMRY4	FXAT9O005FORHS						
119	FXAT9O005FN9N6	FXAT9O005FR72H						
120	FXAT9O005FO05W	FXAT9O005GEMUT						
121	FXAT9O005FOJ8U	FXAT9O005FV8KE						
122	FXAT9O005FOJK6	FXAT9O005FPQN6						
123	FXAT9O005FOS9N	FXAT9O005FUX5D						
124	FXAT9O005FPLI6	FXAT9O005GDAAV						
125	FXAT9O005FQ01H	FXAT9O005FV19Y						
126	FXAT9O005FQ2D4	FXAT9O005GET6S						
127	FXAT9O005FQC3N	FXAT9O005GDVJE						

128	FXAT9O005FQVHK	FXAT9O005FT4OR						
129	FXAT9O005FRIU4	FXAT9O005FZFUW						
130	FXAT9O005FS2SH	FXAT9O005GHABH						
131	FXAT9O005FTO94	FXAT9O005GC2P5						
132	FXAT9O005FTQMA	FXAT9O005FW8RV						
133	FXAT9O005FVTR7	FXAT9O005GCKIG						
134	FXAT9O005FWGOR	FXAT9O005FYM2K						
135	FXAT9O005FWZJG	FXAT9O005GFWAR						
136	FXAT9O005FYCUZ	FXAT9O005GEAZB						
137	FXAT9O005FYL83	FXAT9O005GBMA9						
138	FXAT9O005FZQ5N	FXAT9O005GDDK8						
139	FXAT9O005GAXGR	FXAT9O005GE7C5						
140	FXAT9O005GBJQR	FXAT9O005GDUQ8						
141	FXAT9O005GC3KU	FXAT9O005GEG21						
