# Supplementary material for: Comprehensive transcriptome analysis reveals novel genes involved in cardiac glycoside biosynthesis and mlncRNAs associated with secondary metabolism and stress response in Digitalis purpurea
Source: BMC Genomics. 2012 Jan 10;13:15. doi: 10.1186/1471-2164-13-15 (PMC3269984; doi:10.1186/1471-2164-13-15)
Supplement: Additional file 5 — Conserved mlncRNAs. Complete set of the conserved mlncRNAs. [file 1471-2164-13-15-S5.PDF]

Additional file 5. Conserved mlncRNAs.

Unigene ID	NONCODE npcRNA name
JO460538	u4661_GUT15
JO460779	u185509_mRNAlike
JO460779	u193822_mRNAlike
JO460779	u193815_mRNAlike
JO464980	u202231_mRNAlike
JO466767	u194698_mRNAlike
JO467387	u4661_GUT15
FXAT9O005FM998	u195993_mRNAlike
FXAT9O005FYKFH	u203710_mRNAlike
FXAT9O005F06LM	u198476_mRNAlike
