# Supplementary material for: Comprehensive transcriptome analysis reveals novel genes involved in cardiac glycoside biosynthesis and mlncRNAs associated with secondary metabolism and stress response in Digitalis purpurea
Source: BMC Genomics. 2012 Jan 10;13:15. doi: 10.1186/1471-2164-13-15 (PMC3269984; doi:10.1186/1471-2164-13-15)
Supplement: Additional file 6 — Expression of housekeeping genes. (A) Cq value of ubiquitin, actin and 18s rRNA in leaves (L), stems (S), flowers (F) and roots (R) of one-year-old D. purpurea plants and plantlets treated with cold (C0, C1, C5, C10 and C24) and dehydration (D0, D1, D5, D10 and D24) stresses for 0, 1, 5, 10 and 24 hours. Expression levels were quantified by qPCR. The mean Cq value of three PCR replicates is shown. (B) The expression stability value (M) was calculated by geNorm software [87]. [file 1471-2164-13-15-S6.PDF]

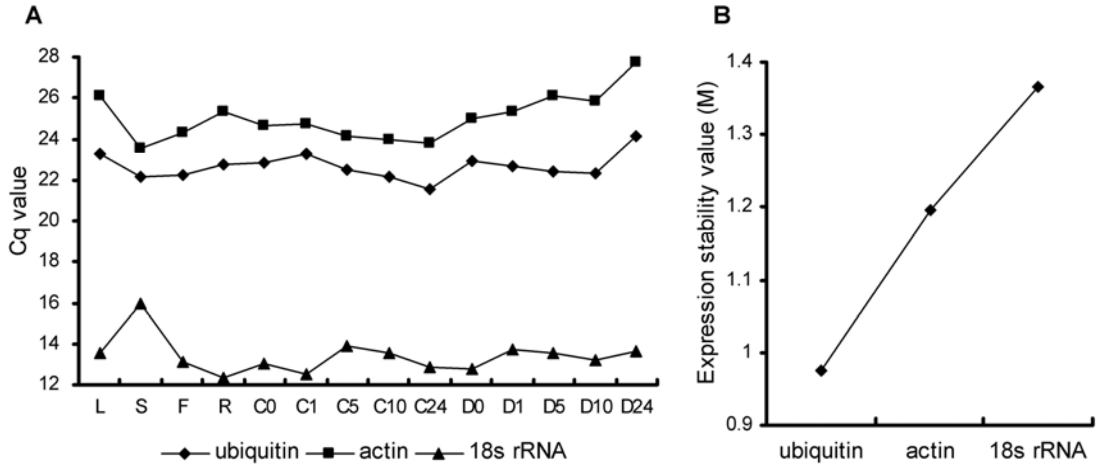

Additional file 6. Expression of housekeeping genes.(A) Cq value of ubiquitin, actin and 18s rRNA in leaves (L), stems (S), flowers (F) and roots (R) of one-year-old *D. purpurea* plants and plantlets treated with cold (C0, C1, C5, C10 and C24) and dehydration (D0, D1, D5, D10 and D24) stresses for 0, 1, 5, 10 and 24 hours. Expression levels were quantified by qPCR. The mean Cq value of three PCR replicates is shown. (B) The expression stability value (M) was calculated by geNorm software [87].
