# Supplementary material for: Comprehensive transcriptome analysis reveals novel genes involved in cardiac glycoside biosynthesis and mlncRNAs associated with secondary metabolism and stress response in Digitalis purpurea
Source: BMC Genomics. 2012 Jan 10;13:15. doi: 10.1186/1471-2164-13-15 (PMC3269984; doi:10.1186/1471-2164-13-15)
Supplement: Additional file 7 — Protein-coding genes significantly homologous or complementary to mlncRNAs. Complete set of the protein-coding genes significantly homologous or complementary to mlncRNAs. [file 1471-2164-13-15-S7.PDF]

**Additional file 7.** Protein-coding genes significantly homologous or complementary to mlncRNAs.

| npcRNA ID | Protein-coding gene ID | Location | Nr annotation |
|---|---|---|---|
| JO460006 | JO461197[b] | CDS | SNF1-related protein kinase |
| JO460015 | FXAT9O005FSUWG[b] | 5'UTR | vacuolar ATP synthase subunit E |
| FXAT9O005FR36L | FXAT9O005FSUWG | 5'UTR | vacuolar ATP synthase subunit E |
| JO460028 | FXAT9O005FWO5W | CDS | predicted protein |
| JO460325 | JO461242 | 5'UTR | conserved hypothetical protein |
| JO460509 | FXAT9O005FSUWG | 5'UTR | vacuolar ATP synthase subunit E |
| JO460521 | JO460856 | 3'UTR | unknown |
| JO460514[a] | JO460857 | CDS-3'UTR junction | hypothetical protein |
| JO460514 | JO467249 | 3'UTR | hypothetical protein |
| JO460514 | JO460955 | 3'UTR | hypothetical protein |
| JO460531 | JO464417 | CDS | hypothetical protein |
| JO460661 | JO460671 | 3'UTR | hypothetical protein |
| JO460702 | JO467278 | CDS | unnamed protein product |
| JO460757 | FXAT9O005FT79C | 3'UTR | predicted protein |
| JO460779 | FXAT9O005FTYXR | 5'UTR | ormdl |
| JO460978 | JO460974 | CDS | hypothetical protein isoform |
| JO461068 | FXAT9O005FQGNV | CDS | predicted protein |
| JO461219 | JO464044 | CDS | peptidyl-prolyl cis-trans isomerase h |
| JO461304 | JO461303 | CDS | calnexin-like protein |
| JO461479 | JO461484 | CDS-3'UTR junction | hypothetical protein |
| JO461576 | JO466590 | CDS | ATP binding protein |
| JO462017 | FXAT9O005FW83K | CDS | hypothetical protein |
| JO462108 [a] | FXAT9O005GALUG | CDS | metallothionein-1 like protein |
| JO462108 | FXAT9O005GC35A | 5'UTR | metallothionein-1 like protein |
| JO462108 | JO462310 | 5'UTR | metallothionein-1 like protein |
| JO462313 | FXAT9O005FQZ9P | 5'UTR | hypothetical protein |
| JO462695 | JO462696 | CDS-3'UTR junction | hypothetical protein |
| JO462891 | FXAT9O005FYUIN[b] | CDS | unnamed protein product |
| JO462924 | Contig03208 | CDS | proliferation-associated 2g4 |
| JO463019 | JO463639 | 3'UTR | 4-hydroxy-3-methylbut-2-en-1-yl diphosphate synthase |
| JO463149 | JO467400 | CDS | hypothetical protein |
| JO463486 | JO466170 | CDS | predicted protein |
| JO464577 | JO467461 | CDS | unknown |
| JO464582 | JO463867[b] | CDS | hypothetical protein isoform 1 |
| JO464673 | JO464672 | CDS | predicted protein |
| JO464756 | JO467430 | CDS | conserved hypothetical protein |
| JO465223 | FXAT9O005F7OVV | CDS | predicted protein |
| JO465358 | JO461680 | 5'UTR-CDS junction | hypothetical protein |
| JO465505 | JO460709 | 3'UTR | dead box ATP-dependent RNA helicase |

| | | | |
|---|---|---|---|
| JO465649 | JO466417 | 3'UTR | predicted protein |
| JO465998 | JO461820 | CDS | predicted protein |
| Contig06550 [a] | JO461396 | CDS | aminocyclopropan-1-carboxylate oxidase |
| JO466083 | JO460546 | CDS | aminocyclopropan-1-carboxylate oxidase |
| FXAT9O005F4QZH | FXAT9O005FYXUF | 3'UTR | nuclear protein skip |
| FXAT9O005FNZ6N | JO467330[b] | CDS | putative DnaJ protein |
| JO466131 | FXAT9O005F9DNM | CDS-3'UTR junction | predicted protein |
| JO466392[a] | FXAT9O005GFKYA | CDS | hypothetical protein |
| JO466338 | JO461584 | CDS | unnamed protein product |
| JO466392 | JO460579 | CDS | hypothetical protein |
| JO466743 | FXAT9O005F2IHG | 3'UTR | hypothetical protein |
| JO466773 | FXAT9O005F1JIM | 3'UTR | unnamed protein product |
| JO466832 | JO460088[b] | CDS-3'UTR junction | plasma membrane polypeptide |
| FXAT9O005FRV45[a] | JO460088 | CDS | plasma membrane polypeptide |
| JO467252 | JO460894 | CDS-3'UTR junction | T6D22.2 |
| FXAT9O005F3P2B | FXAT9O005GD9S5 | CDS | hypothetical protein |
| FXAT9O005FLWAZ | FXAT9O005F23R5[b] | CDS | hypothetical protein |
| FXAT9O005FL209 | FXAT9O005F1E1E | 5'UTR | peptidyl-prolyl cis-trans isomerase |
| FXAT9O005FODAW | FXAT9O005FTITF | CDS-3'UTR junction | unnamed protein product |
| FXAT9O005FRIBK | FXAT9O005FWT6I | 5'UTR | predicted protein |
| FXAT9O005GDIKU | FXAT9O005FPOSE | CDS | hypothetical protein |
| FXAT9O005FZ5ZA | FXAT9O005FSTT4 | CDS-3'UTR junction | hypothetical protein |
| FXAT9O005F2UDI | JO461879 | CDS-3'UTR junction | unknown |
| FXAT9O005GCWAP | FXAT9O005FWPQK | CDS-3'UTR junction | unknown |
| FXAT9O005FSP95 [a] | FXAT9O005GDWC9 | CDS-3'UTR junction | hypothetical protein |
| FXAT9O005FSP95 | FXAT9O005FUWU5 | 3'UTR | TNP1 |
| FXAT9O005FZSI2 [a] | FXAT9O005FZ5UJ | 5'UTR | dihydroflavonal-4-reductase |
| FXAT9O005FZSI2 | FXAT9O005FW6X0 | 5'UTR | Pleiotropic drug resistance protein |
| FXAT9O005FZSI2 | JO462079 | 5'UTR | hypothetical protein |
| FXAT9O005FZSI2 | FXAT9O005FSBBO[b] | 5'UTR | conserved hypothetical protein |
| FXAT9O005F2UKC | JO463099 | CDS | stress-associated protein |
| FXAT9O005FMX26 | FXAT9O005GCFE8 | 5'UTR | unnamed protein product |
| FXAT9O005FSFZD | FXAT9O005F1VAJ | CDS-3'UTR junction | hypothetical protein |
| FXAT9O005FWMI9 | FXAT9O005GE92W | 5'UTR | predicted protein |
| FXAT9O005GCMX1 | FXAT9O005FR6L3 | 3'UTR | hypothetical protein |
| FXAT9O005F06XB | FXAT9O005FVSU7 | CDS | hypothetical protein |
| FXAT9O005F610W | JO461947 | 3'UTR | unnamed protein product |
| FXAT9O005F53JJ | JO461240 | CDS-3'UTR junction | WD-repeat protein |
| FXAT9O005FWTLK | FXAT9O005GC4QX | 3'UTR | S-adenosylmethionine-dependent methyltransferase |
| FXAT9O005GCGMM [a] | JO467235 | 5'UTR | ubiquitin |
| FXAT9O005GCGMM | JO467593 | CDS | polyubiquitin containing 7 ubiquitin monomers |

| | | | |
|---|---|---|---|
| FXAT9O005GCGMM | JO465157 | CDS | polyubiquitin 2 |
| FXAT9O005GCGMM | FXAT9O005F7RR3 | CDS | polyubiquitin containing 7 ubiquitin monomers |
| FXAT9O005GCGMM | JO464779 | CDS | ubiquitin extension protein |
| FXAT9O005GCGMM | JO467592 | CDS | ubiquitin-ribosomal protein fusion S27a |
| FXAT9O005GCGMM | JO460440 | CDS | hypothetical protein OsI_24083 |
| FXAT9O005GCGMM | JO463281 | CDS | similar to polyubiquitin |
| FXAT9O005FMQ2E | FXAT9O005FZZZH[b] | 3'UTR | conserved hypothetical protein |
| FXAT9O005FNEMU | FXAT9O005F1ZQT[b] | CDS | conserved hypothetical protein |
| FXAT9O005FTT7Q | FXAT9O005FWZX1 | CDS | hypothetical protein |
| FXAT9O005FT10Z | FXAT9O005FOC13[b] | 3'UTR | predicted protein |
| FXAT9O005F7OQJ | FXAT9O005FOC13 | 3'UTR | predicted protein |
| FXAT9O005GDPWS | FXAT9O005GC0J1 | 3'UTR | hypothetical protein |
| FXAT9O005FPFI8 | FXAT9O005FS0SX | CDS | hypothetical protein |
| FXAT9O005FRK3I | JO467003 | 3'UTR | small nuclear ribonucleoprotein E |
| FXAT9O005FV18T | FXAT9O005F2EE8 | CDS | conserved hypothetical protein |
| FXAT9O005FO8NQ [a] | FXAT9O005FZCFA | CDS | UDP-glucose:glucosyltransferase |
| FXAT9O005FO8NQ | JO463830 | 5'UTR-CDS junction | UDP-glucose:glucosyltransferase |
| FXAT9O005FML93 | FXAT9O005FSBBO | CDS-3'UTR junction | conserved hypothetical protein |
| FXAT9O005FXCTQ | FXAT9O005GBERO | 5'UTR-CDS junction | Heterogeneous nuclear ribonucleoprotein A1 |
| FXAT9O005FMONJ | FXAT9O005FMAT0 | CDS | hypothetical protein |
| FXAT9O005FMBW1 | FXAT9O005FM6X0 | 3'UTR | GTP-binding protein ERG |
| FXAT9O005F1KZ8 | FXAT9O005GB3KS | CDS-3'UTR junction | conserved hypothetical protein |
| FXAT9O005F945S | FXAT9O005FRHIF | 5'UTR | hypothetical protein |
| FXAT9O005FSEN3 [a] | JO465055 | 5'UTR | seed maturation protein PM37 |
| FXAT9O005FSEN3 | JO461959 | CDS | seed maturation protein PM37 |
| FXAT9O005F09VP [a] | FXAT9O005FSWRG | 3'UTR | alpha-tubulin |
| FXAT9O005F09VP | JO467631 | CDS | alpha-tubulin |
| FXAT9O005F09VP | JO467446 | CDS-3'UTR junction | alpha-tubulin |
| FXAT9O005GAUBZ | FXAT9O005FUFZ2 | CDS-3'UTR junction | Thioredoxin domain-containing protein |
| FXAT9O005FR3O5 | FXAT9O005FZB6N | CDS | hypothetical protein |
| FXAT9O005FSEJE | JO463955 | CDS | hypothetical protein |
| FXAT9O005FZQF2 | FXAT9O005FP0ZS[b] | CDS-3'UTR junction | hypothetical protein |
| FXAT9O005GAJTH[a] | FXAT9O005F4KK2 | 3'UTR | unnamed protein product |
| FXAT9O005GAJTH | JO463948 | CDS-3'UTR junction | HIV-1 rev binding protein, hrbl |
| FXAT9O005F0B8E [a] | FXAT9O005F0HZC | 3'UTR | hypothetical protein |
| FXAT9O005F0B8E | FXAT9O005F5H3U | CDS-3'UTR junction | hypothetical protein |
| FXAT9O005FVAEC | JO461979 | 5'UTR | hypothetical protein |
| FXAT9O005F7WD7 | JO462816 | CDS | predicted protein |
| FXAT9O005FWHZW | JO460467 | CDS-3'UTR junction | unnamed protein product |
| FXAT9O005FQ9SV | JO460976[b] | 3'UTR | hypothetical protein |
| FXAT9O005FW1Q2 | JO466665 | CDS | hypothetical protein |
| FXAT9O005FRH2F | FXAT9O005F9IMP | 5'UTR | conserved hypothetical protein |

| | | | |
|---|---|---|---|
| FXAT9O005FV19Y | JO466753 | 3'UTR | 40S ribosomal protein S10-like |
| FXAT9O005F7ILI | FXAT9O005FZV7J | CDS | hypothetical protein |
| FXAT9O005FN2SG | JO463155 | CDS | unnamed protein product |
| FXAT9O005GEQK3 | FXAT9O005FR2BM | 3'UTR | predicted protein |
| FXAT9O005FP6ID | FXAT9O005F2TDC | CDS | hypothetical protein |
| FXAT9O005FNSUV | FXAT9O005FL17W | CDS | unnamed protein product |
| FXAT9O005GCEKQ | FXAT9O005GCSS5 | 3'UTR | MLO-like protein |
| FXAT9O005F2AKW | JO463344 | 5'UTR | hypothetical protein |
| FXAT9O005FM998 | FXAT9O005F1Y09 | CDS | serine carboxypeptidase |
| FXAT9O005FWVYL | FXAT9O005F2EAQ | 3'UTR | hypothetical protein |
| FXAT9O005FWIL8 | JO464993 | CDS-3'UTR junction | MYB transcription factor |
| FXAT9O005GGHN5 | FXAT9O005GDUI1 | CDS | hypothetical protein |
| FXAT9O005FO3UE | FXAT9O005FSGK9 | CDS | serine/threonine protein kinase |
| FXAT9O005F3035 | FXAT9O005FZBAQ | 5'UTR-CDS junction | DNA binding protein |
| FXAT9O005F77ZO | FXAT9O005GCUYV | CDS-3'UTR junction | putative transport protein |
| FXAT9O005FNCE0 | FXAT9O005GAA7X | 5'UTR-CDS junction | tRNA synthetase class II (D, K and N) family protein |
| FXAT9O005FSXWQ | JO460913 | CDS | conserved hypothetical protein |
| FXAT9O005FWCLL | JO462698 | 3'UTR | predicted protein |
| FXAT9O005F5S8A | JO463448 | CDS | NAD-dependent malic enzyme 62 kDa isoform |
| FXAT9O005FWZJG | FXAT9O005F88BQ | CDS | predicted protein |
| FXAT9O005FNJQH | JO464913 | CDS | hypothetical protein |
| FXAT9O005FPZTR | FXAT9O005FOBCD | 3'UTR | hypothetical protein |
| FXAT9O005FO75B | FXAT9O005FNWSP | 3'UTR | hypothetical protein |
| FXAT9O005GCPNT [a] | FXAT9O005F9K9H [b] | 3'UTR | predicted protein |
| FXAT9O005GCPNT | FXAT9O005FVGBR [b] | 3'UTR | KDEL motif-containing protein 1 precursor |
| FXAT9O005GCPNT | FXAT9O005FZZZH | 3'UTR | conserved hypothetical protein |
| FXAT9O005GCPNT | FXAT9O005F8SEP [b] | 3'UTR | amino acid binding protein |
| FXAT9O005GCPNT | FXAT9O005FNUFS | 3'UTR | hetrotrimeric G protein alpha subunit |
| FXAT9O005GCPNT | FXAT9O005GF46M [b] | 3'UTR | TRFL10 (TRF-LIKE 10); DNA binding |
| FXAT9O005F55I7 [a] | FXAT9O005F11KI | 3'UTR | sugar transporter |
| FXAT9O005F55I7 | FXAT9O005F1YUT | CDS | hypothetical protein isoform 2 |
| FXAT9O005F3FSZ [a] | FXAT9O005GF46M | 3'UTR | TRFL10 (TRF-LIKE 10); DNA binding |
| FXAT9O005F3FSZ | FXAT9O005F8SEP | 3'UTR | amino acid binding protein |
| FXAT9O005F3FSZ | FXAT9O005FVGBR | 3'UTR | KDEL motif-containing protein 1 precursor |
| FXAT9O005F3FSZ | FXAT9O005F9K9H | 3'UTR | predicted protein |
| FXAT9O005F2C22 | FXAT9O005F14MC | 3'UTR | conserved hypothetical protein |
| FXAT9O005GD3WV | FXAT9O005FMSBC | CDS | predicted protein |
| FXAT9O005F3ZYF | FXAT9O005F7R6N | 3'UTR | hypothetical protein At2g02420 |
| FXAT9O005FQ4WN [a] | FXAT9O005F8DWB | CDS-3'UTR junction | zinc finger protein |
| FXAT9O005FQ4WN | FXAT9O005GD7RO | CDS-3'UTR junction | hypothetical protein |
| FXAT9O005F4UKP | JO464469 | 3'UTR | hypothetical protein isoform 1 |

| | | | |
|---|---|---|---|
| FXAT9O005F15V9 | FXAT9O005FYI5G | CDS | RIKEN cDNA 1500006O09, related |
| FXAT9O005FWUG9 | FXAT9O005FYDEO | CDS | cationic amino acid transporter |
| FXAT9O005FLTHG | FXAT9O005F5VF2 | CDS-3'UTR junction | AXS1 (UDP-D-APIOSE/UDP-D-XYLOSE SYNTHASE 1) |
| FXAT9O005F5T1N | FXAT9O005GDSWO | CDS | hypothetical protein |
| FXAT9O005F8I4X | FXAT9O005F4THW | CDS | histone 2 |
| FXAT9O005GDCZW | FXAT9O005FQ6NP | CDS-3'UTR junction | predicted protein |
| FXAT9O005GBFBK | FXAT9O005F7MOH | CDS | predicted protein |
| FXAT9O005F39MA | FXAT9O005FR21D | CDS | transcription factor |
| FXAT9O005FK7A1 | FXAT9O005GFL4V[b] | CDS | transmembraneemp24 domain-containing protein 3 |
| FXAT9O005FXSF1 | FXAT9O005F5ZGN | CDS-3'UTR junction | chaperone binding protein |
| FXAT9O005FR5EW | JO460614 | CDS | hypothetical protein |
| FXAT9O005FYH5F | FXAT9O005F55DU | 3'UTR | unnamed protein product |
| FXAT9O005FUVGQ | FXAT9O005GF2DK | CDS | Plastid-specific 30S ribosomal protein 3, chloroplast precursor |
| FXAT9O005FNH86 | FXAT9O005FO5S4 | 3'UTR | hypothetical protein RCOM_0804080 |
| FXAT9O005GAL5S | FXAT9O005FSOA2 | CDS | hypothetical protein |
| FXAT9O005F7IXD | FXAT9O005F7B8C | CDS | predicted protein |
| FXAT9O005F1YVD | FXAT9O005GCREM | 3'UTR | hypothetical protein |
| FXAT9O005FV8HC | JO465581 | CDS-3'UTR junction | hypothetical protein |
| FXAT9O005FL21T | JO461565 | CDS-3'UTR junction | RHD3 (ROOT HAIR DEFECTIVE 3) |
| FXAT9O005FOHZ4 [a] | FXAT9O005FNEQT | CDS-3'UTR junction | tRNA-dihydrouridine synthase A |
| FXAT9O005FOHZ4 | FXAT9O005FY7BH | CDS-3'UTR junction | tRNA-dihydrouridine synthase A |
| FXAT9O005GCAO0 | JO461279 | CDS | Splicing factor U2af large subunit B |
| FXAT9O005FPQ4I | FXAT9O005F7XIC | CDS-3'UTR junction | predicted protein |
| FXAT9O005FUJ6I | FXAT9O005GCXSS | CDS-3'UTR junction | predicted protein |
| FXAT9O005F4JSP | FXAT9O005FV42K | 3'UTR | similar to NFD2 (NUCLEAR FUSION DEFECTIVE 2) |
| FXAT9O005F7TBH | FXAT9O005GE5AF | CDS | WRKY transcription factor |
| FXAT9O005FM4WQ | FXAT9O005FOMTD | 5'UTR-CDS junction | UPF0737 protein mc410 |
| FXAT9O005FQHQX | FXAT9O005F2VUW | 5'UTR-CDS junction | predicted protein |
| FXAT9O005F64PW | FXAT9O005GA85X | CDS | hypothetical protein |
| FXAT9O005FZG3Y [a] | JO462092 | 5'UTR-CDS junction | 40S ribosomal protein S7 |
| FXAT9O005FZG3Y | JO462094 | CDS | ribosomal protein S7 |
| FXAT9O005FZ67K | FXAT9O005F72X9 | 3'UTR | predicted protein |
| FXAT9O005FORCD[a] | JO463731 | 5'UTR | CBS domain-containing protein |
| FXAT9O005FORCD | FXAT9O005FNLX4 | 5'UTR | CBS domain-containing protein |
| FXAT9O005F1EZT | JO463659 | CDS | predicted protein |
| FXAT9O005GFAJL | FXAT9O005GGBQX | CDS | nitrilase-associated protein |
| FXAT9O005F0CY9 | JO461555 | CDS | hypothetical protein |
| FXAT9O005F5PGF | FXAT9O005F6NPG | CDS | hypothetical protein |
| FXAT9O005FT4UC | FXAT9O005FTZAD | 5'UTR-CDS junction | amine oxidase |

| | | | |
|---|---|---|---|
| FXAT9O005FTCPY | FXAT9O005FY4QP | 5'UTR | SEU1 protein |
| FXAT9O005F0TSP | FXAT9O005FXOMI | CDS-3'UTR junction | hypothetical protein |
| FXAT9O005GBT8P | JO462107 | 3'UTR | hypothetical protein |
| FXAT9O005F63NS | JO465663 | CDS | hypothetical protein |
| FXAT9O005FXOLJ | JO464089 | CDS | hypothetical protein |
| FXAT9O005GDZW2 | FXAT9O005GFZG1 | CDS | conserved hypothetical protein |
| FXAT9O005GDYW9 | FXAT9O005GEK9B | CDS-3'UTR junction | NMDA receptor-regulated protein |
| FXAT9O005GERZE | FXAT9O005FYSWZ | CDS | ZIP transporter |
| FXAT9O005FRLA9 | FXAT9O005GEE9X | CDS | hypothetical protein |
| FXAT9O005FNR9U | FXAT9O005F5WWH | CDS | conserved hypothetical protein |
| FXAT9O005FOCBK | JO463305 | CDS | hypothetical protein |
| FXAT9O005GEMUT [a] | FXAT9O005F5VV5 | 5'UTR | clasp |
| FXAT9O005GEMUT | JO466354 | CDS | putative COX VIIa-like protein |
| FXAT9O005F52IB | FXAT9O005F2ZY3 | 3'UTR | unknown |
| FXAT9O005FNGRT | FXAT9O005GBEF7 | CDS | predicted protein |
| FXAT9O005FM1LP | FXAT9O005F0TJB | CDS-3'UTR junction | hypothetical protein |
| FXAT9O005FOBCV | JO464015 | CDS | hypothetical protein |
| FXAT9O005F9UM5 | FXAT9O005FO1YG | CDS | hypothetical protein |
| FXAT9O005GALZZ | FXAT9O005F02G7 | CDS-3'UTR junction | CONSTANS interacting protein 5 |
| FXAT9O005FMF3S | JO465831 | CDS | unnamed protein product |
| FXAT9O005FYEJ3 | FXAT9O005F1QNG | 5'UTR | hypothetical protein CNBN0200 |
| FXAT9O005FOIWI | FXAT9O005F2HSY | CDS | hypothetical protein |
| FXAT9O005GAO52 | JO460774 | CDS-3'UTR junction | rac-like small GTP-binding protein |
| FXAT9O005FYKFH | JO461339 | CDS-3'UTR junction | hypothetical protein |
| FXAT9O005FM0A7 | JO465802 | CDS | hypersensitive-induced response protein |
| FXAT9O005F1V5D | FXAT9O005FM3XW | CDS | hypothetical protein |
| FXAT9O005FWCY9 | FXAT9O005FNUKB | CDS | Nicotinamide mononucleotide adenylyltransferase |
| FXAT9O005F82SS | FXAT9O005GDUHY | CDS | hypothetical protein |
| FXAT9O005GDHJU | FXAT9O005FRTUV | 3'UTR | hypothetical protein OsI_00033 |
| FXAT9O005F0SB6 [a] | JO460944 | 3'UTR | hypothetical protein LOC100193910 |
| FXAT9O005F0SB6 | Contig01058 | CDS | similar to 60S ribosomal protein L34 |
| FXAT9O005GC51I | FXAT9O005FNYNJ | CDS | calmodulin-binding heat-shock protein |
| FXAT9O005F01EY | JO461325[b] | 3'UTR | hypothetical protein |
| FXAT9O005FUA8K | FXAT9O005GEVW7 | CDS | hypothetical protein |
| FXAT9O005F305C | JO462532 | CDS | unnamed protein product |
| FXAT9O005FWA3N | FXAT9O005FP0ZS | CDS-3'UTR junction | hypothetical protein |
| FXAT9O005F3IVQ | FXAT9O005GDBM4 | CDS | conserved hypothetical protein |
| FXAT9O005F0DYU | FXAT9O005F4P7E | CDS | predicted protein |
| FXAT9O005FUP4B | FXAT9O005F23R5 | CDS-3'UTR junction | hypothetical protein |
| FXAT9O005GALC7 [a] | JO461648[b] | CDS-3'UTR junction | unnamed protein product |
| FXAT9O005GALC7 | FXAT9O005F23R5 | 5'UTR | hypothetical protein |
| FXAT9O005GBFA7[a] | JO461648 | CDS-3'UTR junction | unnamed protein product |

| | | | |
|---|---|---|---|
| FXAT9O005GBFA7 | FXAT9O005F23R5 | 5'UTR | hypothetical protein |
| FXAT9O005FVZ2Q[a] | JO461648 | CDS | unnamed protein product |
| FXAT9O005FVZ2Q | FXAT9O005F23R5 | CDS | hypothetical protein |
| FXAT9O005FVZ2Q | FXAT9O005FYUIN | CDS | unnamed protein product |
| FXAT9O005FRYWW | FXAT9O005FUKEC | CDS | predicted protein |
| FXAT9O005F6Z7G | JO464579 | CDS | Armadillo repeat-containing protein |
| FXAT9O005GAK1H | JO466307 | CDS-3'UTR junction | hypothetical protein |
| FXAT9O005FOZDO | FXAT9O005FVRHK | CDS | predicted protein |
| FXAT9O005GE9UX | FXAT9O005FPEHD | CDS | hypothetical protein [Vitis vinifera] |
| FXAT9O005FYD4C | FXAT9O005FK4AG | CDS | aromatic amino acid decarboxylase |
| FXAT9O005F11SO | FXAT9O005FZYO5 | CDS-3'UTR junction | similar to plectin-related |
| FXAT9O005FZPZT | FXAT9O005FUUEG | CDS | predicted protein |
| FXAT9O005GG0S2 | FXAT9O005FNEQL | CDS | hypothetical protein |
| FXAT9O005F0U3Z | JO463543 | CDS-3'UTR junction | similar to chaperonin containing TCP-1 complex gamma chain |
| FXAT9O005FQB5Z | FXAT9O005F8DZ7 | CDS | putative transcription factor |
| FXAT9O005FYWNU | FXAT9O005FZ54P | CDS | hypothetical protein |
| FXAT9O005F99FZ | FXAT9O005FU1JJ[b] | CDS | predicted protein |
| FXAT9O005FUDB7 | FXAT9O005FU1JJ | CDS | predicted protein |
| FXAT9O005F5JJ8 | JO464908 | CDS | predicted protein |
| FXAT9O005FQUTZ | FXAT9O005F7A7W | CDS | hypothetical protein |
| FXAT9O005FSSMD | FXAT9O005F6Q1H | CDS-3'UTR junction | protein arginine n-methyltransferase |
| FXAT9O005FY1YH[a] | FXAT9O005FRDS2 | CDS-3'UTR junction | predicted protein |
| FXAT9O005FY1YH | FXAT9O005FMM89 | CDS | hypothetical protein |
| FXAT9O005FT6G0 | JO466897 | CDS | predicted protein |
| FXAT9O005F1H79 | JO462284 | 3'UTR | hypothetical protein |
| FXAT9O005GFV95 | FXAT9O005FX4TF | CDS | hypothetical protein |
| FXAT9O005GEY4H | FXAT9O005FRKIX | CDS | conserved hypothetical protein |
| FXAT9O005FTXHT | FXAT9O005GAWOC | CDS | predicted protein |
| FXAT9O005FU8T1[a] | FXAT9O005F48ZC | CDS | hypothetical protein |
| FXAT9O005FU8T1 | FXAT9O005GDLPI | CDS | predicted protein |
| FXAT9O005FPPU4 | FXAT9O005FMPJO | CDS-3'UTR junction | predicted protein |
| FXAT9O005F2J1G | FXAT9O005F0WCY | CDS | hypothetical protein |
| FXAT9O005GC5H4 | JO466487 | 3'UTR | unknown |
| FXAT9O005F3FC5[a] | FXAT9O005F0M1Q | CDS | unnamed protein product |
| FXAT9O005F3FC5 | FXAT9O005FZAQP | CDS | predicted protein |
| FXAT9O005F3FC5 | FXAT9O005F8GBG | CDS | Superoxide dismutase [Cu-Zn] 2 |
| FXAT9O005FNRF3 | JO465301 | CDS | actin-depolymerizing factor |
| FXAT9O005FZ54T | FXAT9O005FL7XG | CDS-3'UTR junction | hypothetical protein |
| FXAT9O005F6JNJ | FXAT9O005F8H8N | 5'UTR-CDS junction | predicted protein |
| FXAT9O005F3TN0 | JO461197 | 3'UTR | SNF1-related protein kinase |
| FXAT9O005F964C | FXAT9O005FQHPG | CDS | Protein YME1 |
| FXAT9O005GDYZC | FXAT9O005GBKC4 | CDS | hypothetical protein |

| | | | |
|---|---|---|---|
| FXAT9O005F1GNX | JO464278 | 3'UTR | dead box ATP-dependent RNA helicase |
| FXAT9O005FS4CW | FXAT9O005FMQ1A | CDS | hypothetical protein |
| FXAT9O005FN1PS | FXAT9O005F5L1E | CDS-3'UTR junction | mitochondrial ATPase beta subunit |
| FXAT9O005F07SS | FXAT9O005GBFT8 | CDS | hypothetical protein |
| FXAT9O005FQ84D | FXAT9O005FNJEX | CDS | unnamed protein product |
| FXAT9O005GF9JQ | FXAT9O005F788O | CDS | hypothetical protein |
| FXAT9O005FPTSW | JO463912 | CDS | UMP synthase |
| FXAT9O005FYRML | JO461325 | 3'UTR | hypothetical protein |
| FXAT9O005FRV45 | JO461950 | CDS | DREPP2 protein |
| FXAT9O005F5YLB | JO462000 | CDS | eukaryotic translation initiation factor 2 beta subunit-like |
| FXAT9O005FTJU1 | FXAT9O005FXROD | 5'UTR-CDS junction | conserved hypothetical protein |
| FXAT9O005GADO1 [a] | JO464429 | CDS | mta/sah nucleosidase |
| FXAT9O005GADO1 | FXAT9O005F49QU[b] | CDS | predicted protein |
| FXAT9O005F0MIM | JO460458 | CDS | hypothetical protein |
| FXAT9O005F61NY | FXAT9O005F194Z | 5'UTR-CDS junction | Transaldolase |
| FXAT9O005FYDPV | JO466097 | 5'UTR-CDS junction | beta-galactosidase |
| FXAT9O005F2C05 | JO464487 | CDS-3'UTR junction | conserved hypothetical protein |
| FXAT9O005F6W1P [a] | FXAT9O005FO34D | 5'UTR-CDS junction | DNA binding protein |
| FXAT9O005F6W1P | FXAT9O005F0KWL | CDS | hypothetical protein |
| FXAT9O005FXSWQ | FXAT9O005F2NJG | CDS | hypothetical protein |
| FXAT9O005FVX79 | JO462377 | CDS | hypothetical protein |
| FXAT9O005GDE8H | FXAT9O005FQ6RE | CDS | conserved hypothetical protein |
| FXAT9O005FVFI4 | FXAT9O005FN1RJ | CDS | similar to transducin family protein / WD-40 repeat family protein |
| FXAT9O005FU1DI | FXAT9O005GE4GF | CDS | predicted protein |
| FXAT9O005GF20N | FXAT9O005FYBNE | 3'UTR | copine |
| FXAT9O005FWJJ7 | JO466076 | CDS | Poly(rC)-binding protein |
| FXAT9O005F1O9C [a] | JO462690 | CDS | conserved hypothetical protein |
| FXAT9O005F1O9C | FXAT9O005FUW8H | CDS-3'UTR junction | hypothetical protein |
| FXAT9O005FZCDP | JO462428 | CDS-3'UTR junction | Os03g0109700 |
| FXAT9O005FOX82 | JO460364 | CDS | hypothetical protein |
| FXAT9O005FOI0W | JO466537 | CDS | hypothetical protein |
| FXAT9O005FWQ1Z | FXAT9O005FZC1G | CDS | hypothetical protein |
| FXAT9O005FM480 | FXAT9O005FPDYQ | CDS | predicted protein |
| FXAT9O005F4269 | JO463867 | 3'UTR | predicted protein |
| FXAT9O005GBZR9 | JO462004 | CDS | hypothetical protein |
| FXAT9O005FO0OA | FXAT9O005F2M6L | CDS | predicted protein |
| FXAT9O005FZ2TR | FXAT9O005FUNEP | CDS | hypothetical protein |
| FXAT9O005F6XU6 | FXAT9O005FY0LN | CDS | ubiquitin-conjugating enzyme E2 |
| FXAT9O005F3FCK | FXAT9O005GEL2O | 5'UTR-CDS junction | ATP phosphoribosyltransferase |
| FXAT9O005FS6LV | FXAT9O005GE3VJ | 5'UTR-CDS junction | hypothetical protein |
| FXAT9O005FZM3V | FXAT9O005F0EUH | 5'UTR-CDS junction | conserved hypothetical protein |

| | | | |
|---|---|---|---|
| FXAT9O005FV1PR | FXAT9O005F638N | CDS | nucleic acid binding protein |
| FXAT9O005FRNM3 | JO467066 | CDS | methylmalonate-semialdehyde dehydrogenase |
| FXAT9O005FUOYM | FXAT9O005F8LV8 | CDS | hypothetical protein |
| FXAT9O005FNL1K [a] | JO460976 | 5'UTR-CDS junction | 2-cys peroxiredoxin |
| FXAT9O005FNL1K | JO463397 | CDS | peroxiredoxins, prx-1, prx-2, prx-3 |
| FXAT9O005FRSLG | FXAT9O005GAHSO | CDS-3'UTR junction | unknown |
| FXAT9O005FOL4X | FXAT9O005F2ABC | CDS | conserved hypothetical protein |
| FXAT9O005F3C2X | JO463507 | 5'UTR-CDS junction | hypothetical protein |
| FXAT9O005GE1ER | FXAT9O005GCP3D | 5'UTR-CDS junction | hypothetical protein isoform 1 |
| FXAT9O005GAAOB | JO463143 | CDS | transferase, transferring glycosyl groups |
| FXAT9O005F4HX5 [a] | JO464848 | CDS | unknown protein |
| FXAT9O005F4HX5 | FXAT9O005F0FNQ | CDS | Thylakoid membrane phosphoprotein 14 kDa, chloroplast precursor |
| FXAT9O005F7251 [a] | FXAT9O005GC2U5 | CDS-3'UTR junction | predicted protein |
| FXAT9O005F7251 | FXAT9O005FRO7J | CDS | predicted protein |
| FXAT9O005F13RE | FXAT9O005F6334 | 3'UTR | predicted protein |
| FXAT9O005FVI0M | FXAT9O005FT86Y | 3'UTR | hypothetical protein |
| FXAT9O005FYDT4 | FXAT9O005GDJKY | CDS | nonsense-mediated mRNA decay protein |
| FXAT9O005GEXIP | JO460458 | CDS | putative 60S ribosomal protein L39 |
| FXAT9O005GDX02 | FXAT9O005F194Z | CDS | hypothetical protein |
| FXAT9O005F8DH5 | JO466097 | 3'UTR | hypothetical protein |
| FXAT9O005GBB97 | JO464487 | CDS | beta-ketoacyl-ACP synthase II |
| FXAT9O005F1ZGN [a] | FXAT9O005FO34D | 3'UTR | predicted protein |
| FXAT9O005F1ZGN | FXAT9O005F0KWL | 3'UTR | Protein grpE |
| FXAT9O005F8J63 | FXAT9O005F2NJG | 5'UTR | solanesyl diphosphate synthase |
| FXAT9O005GD1W7 | JO462377 | CDS | Phytoene dehydrogenase |
| FXAT9O005F3QD2 | FXAT9O005FQ6RE | CDS | uncharacterized plant-specific domain TIGR01589 family protein |
| FXAT9O005FQE5Y | FXAT9O005FN1RJ | 5'UTR-CDS junction | predicted protein |
| FXAT9O005FW68D | FXAT9O005GE4GF | CDS-3'UTR junction | predicted protein |
| FXAT9O005FZWJ1 | FXAT9O005FYBNE | CDS | unnamed protein product |
| FXAT9O005GD055 | JO466076 | CDS | hypothetical protein |
| FXAT9O005FOW7X | JO462690 | CDS | hypothetical protein |
| FXAT9O005GF9L8 | FXAT9O005FUW8H | CDS-3'UTR junction | protein disulfide isomerase |
| FXAT9O005F5D12 | JO462428 | CDS | unnamed protein product |
| FXAT9O005FMCD0 | FXAT9O005FWDAO[b] | 5'UTR | nucleic acid binding protein |
| FXAT(O005FTPYO | FXAT9O005FWDAO | 5'UTR-CDS junction | |
| FXAT9O005FQ2MG | JO460151 | 3'UTR | hypothetical protein |
| FXAT9O005F531X [a] | JO460566 | 5'UTR-CDS junction | hypothetical protein |
| FXAT9O005F531X | JO460567 | CDS | hypothetical protein |
| FXAT9O005F7H45 | FXAT9O005FZGP5 | CDS | unknown |
| FXAT9O005FQO26 | FXAT9O005FZ8UK | CDS | hypothetical protein |
| FXAT9O005FUJUB [a] | JO461881 | CDS | hypothetical protein |

| | | | |
|---|---|---|---|
| FXAT9O005FUJUB | JO467349 | CDS | hypothetical protein |
| FXAT9O005FQ8EN | FXAT9O005F0WV0 | CDS | PLE |
| FXAT9O005GCYDK | FXAT9O005FXWJB | CDS | hypothetical protein |
| FXAT9O005FP7WS | JO462467 | CDS | predicted protein |
| FXAT9O005FORO9 | FXAT9O005FTY2K | CDS | conserved hypothetical protein |
| FXAT9O005GAL5E | FXAT9O005FZ6EL | 3'UTR | predicted protein |
| FXAT9O005GBSC8 | FXAT9O005FN0VY | 3'UTR | polynucleotide kinase- 3'-phosphatase |
| FXAT9O005GBW9Y | JO461338 | CDS | amino acid transporter |
| FXAT9O005FRM6S | FXAT9O005FVUK2 | CDS | predicted protein |
| FXAT9O005FXMSN | FXAT9O005FXNZ8 | 3'UTR | hypothetical protein |
| FXAT9O005F31A2 | FXAT9O005FO3UI | 5'UTR-CDS junction | hypothetical protein |
| FXAT9O005FR9MT | FXAT9O005F1ZQT | 5'UTR-CDS junction | conservedhypothetical |
| FXAT9O005GDOMW | FXAT9O005FNDYO | 3'UTR | hypothetical protein |
| FXAT9O005F3R8I [a] | FXAT9O005FVFJ2[b] | 5'UTR | hypothetical protein |
| FXAT9O005F3R8I | FXAT9O005FOWHE[b] | 5'UTR-CDS junction | predicted protein |
| FXAT9O005F3R8I | FXAT9O005F6QS8[b] | 5'UTR | hypothetical protein |
| FXAT9O005GGX5A | FXAT9O005FWEHI | 3'UTR | Protein MSP1 |
| FXAT9O005F72MK | FXAT9O005FY6XF | CDS | putative arginine decarboxylase |
| FXAT9O005FR6KL | FXAT9O005F7HZV | 5'UTR-CDS junction | metal ion binding protein |
| FXAT9O005F3PZP | FXAT9O005GAJIB | CDS | SWIb domain-containing protein |
| FXAT9O005FVD38 | JO467521 | CDS | unknown |
| FXAT9O005GDWHE | FXAT9O005FPF8A | 5'UTR-CDS junction | conserved hypothetical protein |
| FXAT9O005FRTXJ | JO462031 | 5'UTR | protein phosphatase 2c |
| FXAT9O005GDGWK [a] | FXAT9O005F6TWI | CDS | glycosyltransferase 5 |
| FXAT9O005GDGWK | JO461045 | CDS | hypothetical protein |
| FXAT9O005GDGWK | JO460326 | 3'UTR | heat shock protein 17.5 |
| FXAT9O005GFQZ3 | FXAT9O005FRI7U | CDS-3'UTR junction | hypothetical protein |
| FXAT9O005F2TCR | FXAT9O005F6QDJ | 3'UTR | hypothetical protein |
| FXAT9O005F85NK | FXAT9O005GEYWL | CDS | hypothetical protein |
| FXAT9O005FS6FU [a] | FXAT9O005F67CD | 5'UTR-CDS junction | mta/sah nucleosidase |
| FXAT9O005FS6FU | FXAT9O005F49QU | CDS | predicted protein |
| FXAT9O005F7Y62 | JO461485 | CDS-3'UTR junction | auxin resistance protein |
| FXAT9O005FPT5R [a] | JO463148 | CDS | cysteine protease |
| FXAT9O005FPT5R | JO463089 | CDS | predicted protein |
| FXAT9O005F8DN9 | FXAT9O005FQJ1L | CDS | hypothetical protein |
| FXAT9O005F2M84 | FXAT9O005FMT4D | CDS | hypothetical protein |
| FXAT9O005F16PH | JO467150 | 5'UTR | protein kinase |
| FXAT9O005FN1PA | JO466303 | CDS | conserved hypothetical protein |
| FXAT9O005GGEFV | FXAT9O005F123M | 5'UTR-CDS junction | predicted protein |
| FXAT9O005FRF3Y[a] | FXAT9O005GBT4Y | CDS | hypothetical protein |
| FXAT9O005FRF3Y | FXAT9O005F9R1Q | CDS | hypothetical protein |
| FXAT9O005FXDND | FXAT9O005FS513[b] | CDS-3'UTR junction | predicted protein |
| FXAT9O005GC2PW | FXAT9O005FS513 | CDS-3'UTR junction | |

| | | | |
|---|---|---|---|
| FXAT9O005F51M0 | JO462708 | CDS | hypothetical protei |
| FXAT9O005F9V0Y | JO467330 | 5'UTR | putative DnaJ protein |
| FXAT9O005F32MA [a] | JO467328[b] | 5'UTR | xyloglucan endotransglucosylase/hydrolase 4 |
| FXAT9O005F32MA | FXAT9O005GC3X8[b] | CDS | acetyl coa carboxylase pRS1 |
| FXAT9O005F32MA | FXAT9O005FLAN4[b] | 5'UTR | hypothetical protein |
| FXAT9O005F32MA | FXAT9O005F2BUA[b] | 5'UTR | GNOM-like 1 protein |
| FXAT9O005F32MA | FXAT9O005F4WF9[b] | CDS | ERF transcription factor 5 |
| FXAT9O005F32MA | FXAT9O005FUPFH[b] | 5'UTR | protein phosphatase 2a, regulatory subunit |
| FXAT9O005F32MA | FXAT9O005FOWHE | 5'UTR | predicted protein |
| FXAT9O005F32MA | FXAT9O005FTIKD[b] | 5'UTR | hypothetical protein |
| FXAT9O005F32MA | FXAT9O005FZX7U[b] | 5'UTR | hypothetical protein |
| FXAT9O005F32MA | FXAT9O005FUAW3[b] | 5'UTR | protein phosphatase 2A 65 kDa regulatory subunit |
| FXAT9O005F32MA | FXAT9O005F0C86[b] | 5'UTR | hypothetical protein |
| FXAT9O005FPWUE [a] | JO467328 | 5'UTR | xyloglucan endotransglucosylase/hydrolase 4 |
| FXAT9O005FPWUE | FXAT9O005FUAW3 | 5'UTR | protein phosphatase 2A 65 kDa regulatory subunit |
| FXAT9O005FPWUE | FXAT9O005FVFJ2 | 5'UTR | hypothetical protein |
| FXAT9O005FPWUE | FXAT9O005F4WF9 | CDS | ERF transcription factor 5 |
| FXAT9O005FPWUE | FXAT9O005FLAN4 | 5'UTR | hypothetical protein |
| FXAT9O005FPWUE | FXAT9O005FUPFH | 5'UTR | protein phosphatase 2a, regulatory subunit |
| FXAT9O005FPWUE | FXAT9O005F0C86 | 5'UTR | hypothetical protein |
| FXAT9O005FPWUE | FXAT9O005GC3X8 | CDS | acetyl coa carboxylase pRS1 |
| FXAT9O005FPWUE | FXAT9O005F2BUA | 5'UTR | GNOM-like 1 protein |
| FXAT9O005FPWUE | FXAT9O005FOWHE | 5'UTR | predicted protein |
| FXAT9O005FPWUE | FXAT9O005FTIKD | 5'UTR | hypothetical protein |
| FXAT9O005FPWUE | FXAT9O005FZX7U | CDS | hypothetical protein |
| FXAT9O005FXDAT [a] | FXAT9O005FUAW3 | 5'UTR | protein phosphatase 2A 65 kDa regulatory subunit |
| FXAT9O005FXDAT | FXAT9O005GC3X8 | CDS | acetyl coa carboxylase pRS1 |
| FXAT9O005FXDAT | FXAT9O005FLAN4 | 5'UTR | hypothetical protein |
| FXAT9O005FXDAT | FXAT9O005F2BUA | 5'UTR | GNOM-like 1 protein |
| FXAT9O005FXDAT | FXAT9O005FUPFH | 5'UTR | protein phosphatase 2a, regulatory subunit |
| FXAT9O005FXDAT | FXAT9O005FOWHE | 5'UTR | predicted protein |
| FXAT9O005FXDAT | FXAT9O005FTIKD | 5'UTR | hypothetical protein |
| FXAT9O005FXDAT | FXAT9O005FZX7U | CDS | hypothetical protein |
| FXAT9O005FXDAT | FXAT9O005F0C86 | 5'UTR | hypothetical protein |
| FXAT9O005FXDAT | JO467328 | 5'UTR | Xyloglucan endotransglucosylase/hydrolase 4 |
| FXAT9O005FW3GF[a] | FXAT9O005F0C86 | 5'UTR | hypothetical protein |
| FXAT9O005FW3GF | FXAT9O005FUPFH | 5'UTR | protein phosphatase 2a, regulatory subunit |
| FXAT9O005FW3GF | FXAT9O005FLAN4 | 5'UTR | hypothetical protein |
| FXAT9O005FW3GF | FXAT9O005FUAW3 | 5'UTR | protein phosphatase 2A 65 kDa regulatory subunit |

| | | | |
|---|---|---|---|
| FXAT9O005FW3GF | FXAT9O005FVFJ2 | 5'UTR | hypothetical protein |
| FXAT9O005FW3GF | FXAT9O005GC3X8 | CDS | acetyl coa carboxylase pRS1 |
| FXAT9O005FW3GF | FXAT9O005F2BUA | 5'UTR | GNOM-like 1 protein |
| FXAT9O005FW3GF | FXAT9O005FOWHE | 5'UTR | predicted protein |
| FXAT9O005FW3GF | FXAT9O005FTIKD | 5'UTR | hypothetical protein |
| FXAT9O005FW3GF | FXAT9O005FZX7U | CDS | hypothetical protein |
| FXAT9O005FXA3E[a] | FXAT9O005FTIKD | 5'UTR | hypothetical protein |
| FXAT9O005FXA3E | FXAT9O005FZX7U | CDS | hypothetical protein |
| FXAT9O005FXA3E | FXAT9O005F2BUA | 5'UTR | GNOM-like 1 protein |
| FXAT9O005FXA3E | FXAT9O005FOWHE | 5'UTR | predicted protein |
| FXAT9O005FXA3E | FXAT9O005FR7Y1[b] | 5'UTR | n6-DNA-methyltransferase |
| FXAT9O005FXA3E | FXAT9O005GC3X8 | CDS | acetyl coa carboxylase pRS1 |
| FXAT9O005FXA3E | FXAT9O005FLAN4 | 5'UTR | hypothetical protein |
| FXAT9O005FXA3E | FXAT9O005FUPFH | 5'UTR | protein phosphatase 2a, regulatory subunit |
| FXAT9O005FXA3E | FXAT9O005FMKVN[b] | CDS | hypothetical protein |
| FXAT9O005FXA3E | FXAT9O005F0C86 | 5'UTR | hypothetical protein |
| FXAT9O005FXA3E | JO462056[b] | 5'UTR | predicted protein |
| FXAT9O005FXA3E | FXAT9O005FRYCU[b] | CDS | hypothetical protein |
| FXAT9O005FXA3E | FXAT9O005F6QS8 | CDS | hypothetical protein |
| FXAT9O005FXA3E | FXAT9O005FUAW3 | 5'UTR | protein phosphatase 2A 65 kDa regulatory subunit |
| FXAT9O005FXA3E | FXAT9O005FL5V2[b] | CDS | polygalacturonase |
| FXAT9O005FXA3E | FXAT9O005FVFJ2 | 5'UTR | hypothetical protein |
| FXAT9O005FXA3E | FXAT9O005FWWD7[b] | CDS | hypothetical protein |
| FXAT9O005FXA3E | FXAT9O005FPQRB | CDS | hypothetical protein |
| FXAT9O005FXA3E | FXAT9O005FVO3C | CDS | small zinc finger-like protein |
| FXAT9O005FXA3E | JO467328 | 5'UTR | xyloglucan endotransglucosylase/hydrolase 4 |
| FXAT9O005FNNS5[a] | FXAT9O005FLAN4 | 5'UTR | hypothetical protein |
| FXAT9O005FNNS5 | FXAT9O005FUPFH | 5'UTR | protein phosphatase 2a, regulatory subunit |
| FXAT9O005FNNS5 | FXAT9O005F0C86 | 5'UTR | hypothetical protein |
| FXAT9O005FNNS5 | FXAT9O005GC3X8 | CDS | acetyl coa carboxylase pRS1 |
| FXAT9O005FNNS5 | FXAT9O005F2BUA | 5'UTR | GNOM-like 1 protein |
| FXAT9O005FNNS5 | FXAT9O005FOWHE | 5'UTR | predicted protein |
| FXAT9O005FNNS5 | FXAT9O005FTIKD | 5'UTR | hypothetical protein |
| FXAT9O005FNNS5 | FXAT9O005FZX7U | CDS | hypothetical protein |
| FXAT9O005FNNS5 | FXAT9O005FVFJ2 | 5'UTR | hypothetical protein |
| FXAT9O005FNNS5 | FXAT9O005FR7Y1 | 5'UTR | n6-DNA-methyltransferase |
| FXAT9O005FNNS5 | FXAT9O005FUAW3 | 5'UTR | protein phosphatase 2A 65 kDa regulatory subunit |
| FXAT9O005FNNS5 | FXAT9O005FMKVN | CDS | hypothetical protein |
| FXAT9O005FNNS5 | FXAT9O005F6QS8 | CDS | hypothetical protein |
| FXAT9O005F63SF[a] | FXAT9O005FZX7U | CDS | hypothetical protein |
| FXAT9O005F63SF | FXAT9O005FTIKD | 5'UTR | hypothetical protein |

| | | | |
|---|---|---|---|
| FXAT9O005F63SF | FXAT9O005F2BUA | 5'UTR | GNOM-like 1 protein |
| FXAT9O005F63SF | FXAT9O005FOWHE | 5'UTR | predicted protein |
| FXAT9O005F63SF | FXAT9O005GC3X8 | CDS | acetyl coa carboxylase pRS1 |
| FXAT9O005F63SF | FXAT9O005FLAN4 | 5'UTR | hypothetical protein |
| FXAT9O005F63SF | FXAT9O005FUPFH | 5'UTR | protein phosphatase 2a, regulatory subunit |
| FXAT9O005F63SF | FXAT9O005FR7Y1 | 5'UTR | n6-DNA-methyltransferase |
| FXAT9O005F63SF | FXAT9O005F0C86 | 5'UTR | hypothetical protein |
| FXAT9O005F63SF | FXAT9O005FMKVN | CDS | hypothetical protein |
| FXAT9O005F63SF | JO462056 | 5'UTR | predicted protein |
| FXAT9O005F63SF | FXAT9O005FWWD7 | CDS | hypothetical protein |
| FXAT9O005F63SF | FXAT9O005FRYCU | CDS | hypothetical protein |
| FXAT9O005F63SF | FXAT9O005F6QS8 | CDS | hypothetical protein |
| FXAT9O005F63SF | FXAT9O005FUAW3 | 5'UTR | protein phosphatase 2A 65 kDa regulatory subunit |
| FXAT9O005F63SF | FXAT9O005FL5V2 | CDS | polygalacturonase |
| FXAT9O005F63SF | FXAT9O005FVFJ2 | 5'UTR | hypothetical protein |
| FXAT9O005F2XOS[a] | FXAT9O005GC3X8 | CDS | acetyl coa carboxylase pRS1 |
| FXAT9O005F2XOS | FXAT9O005FLAN4 | 5'UTR | hypothetical protein |
| FXAT9O005F2XOS | FXAT9O005FUPFH | 5'UTR | protein phosphatase 2a, regulatory subunit |
| FXAT9O005F2XOS | FXAT9O005F0C86 | 5'UTR | hypothetical protein |
| FXAT9O005F2XOS | FXAT9O005F2BUA | 5'UTR | GNOM-like 1 protein |
| FXAT9O005F2XOS | FXAT9O005FOWHE | 5'UTR | predicted protein |
| FXAT9O005F2XOS | FXAT9O005FTIKD | 5'UTR | hypothetical protein |
| FXAT9O005F2XOS | FXAT9O005FZX7U | CDS | hypothetical protein |
| FXAT9O005F2XOS | FXAT9O005FR7Y1 | 5'UTR | n6-DNA-methyltransferase |
| FXAT9O005F2XOS | FXAT9O005FUAW3 | 5'UTR | protein phosphatase 2A 65 kDa regulatory subunit |
| FXAT9O005F2XOS | FXAT9O005FVFJ2 | 5'UTR | hypothetical protein |
| FXAT9O005F2XOS | FXAT9O005FMKVN | CDS | hypothetical protein |
| FXAT9O005F2XOS | JO467328 | 5'UTR | xyloglucan endotransglucosylase/hydrolase 4 |
| FXAT9O005F2XOS | FXAT9O005FRYCU | CDS | hypothetical protein |
| FXAT9O005F2XOS | FXAT9O005F6QS8 | CDS | hypothetical protein |
| FXAT9O005F2XOS | FXAT9O005FL5V2 | CDS | polygalacturonase |
| FXAT9O005F2XOS | JO462056 | 5'UTR | predicted protein |
| FXAT9O005FQFJJ | FXAT9O005F8OTJ | CDS | hypothetical protein |
| FXAT9O005GEZ76 | FXAT9O005F6N3V | 5'UTR | predicted protein |
| FXAT9O005F9CFA | JO466688 | CDS-3'UTR junction | predicted protein |
| FXAT9O005F6POF | FXAT9O005FZZUR | 3'UTR | hypothetical protein |
| FXAT9O005GATGN | FXAT9O005F7S38 | CDS | 4Fe-4S ferredoxin, iron-sulfur binding |
| FXAT9O005FUREW | FXAT9O005F6SW7 | CDS | ubiquitin-conjugating enzyme |
| FXAT9O005GFVPW[a] | FXAT9O005GFL4V | CDS | unknown |
| FXAT9O005GFVPW | FXAT9O005FTZ45 | 5'UTR | unknown protein |
| FXAT9O005GFVPW | FXAT9O005F7JI9 | 5'UTR | predicted protein |

| | | | |
|---|---|---|---|
| FXAT9O005GFVPW | FXAT9O005FVO3C | 5'UTR | small zinc finger-like protein |
| FXAT9O005GFVPW | FXAT9O005FKU6J | 5'UTR | hypothetical protein |
| FXAT9O005GELM2 | FXAT9O005FMKPV | CDS | hypothetical protein |
| FXAT9O005GDLZ6 | JO464210 | CDS | hypothetical protein |
| FXAT9O005GHG2Y | FXAT9O005FW9EJ | 3'UTR | ZEITLUPE |
| FXAT9O005FYBD9 | FXAT9O005FNNL3 | CDS | hypothetical protein |
| FXAT9O005F8YKK | JO463826 | CDS | hypothetical protein |

Location refers to region of protein-coding genes that show homologous or complementary to mlncRNA . CDS: coding sequences ; UTR: untranslated region. [a]. mlncRNAs hit more than one protein-coding genes ; [b]. protein-coding genes hit more than one mlncRNAs.
