# Supplementary material for: Comprehensive transcriptome analysis reveals novel genes involved in cardiac glycoside biosynthesis and mlncRNAs associated with secondary metabolism and stress response in Digitalis purpurea
Source: BMC Genomics. 2012 Jan 10;13:15. doi: 10.1186/1471-2164-13-15 (PMC3269984; doi:10.1186/1471-2164-13-15)
Supplement: Additional file 8 — The putative biosynthetic pathway of cardiac glycosides in Digitalis purpurea. The pathway roughly comprises terpenoid backbone biosynthesis, steroid biosynthesis and cardenolide biosynthesis three stages. The enzymes with corresponding unigenes are framed. [file 1471-2164-13-15-S8.PDF]

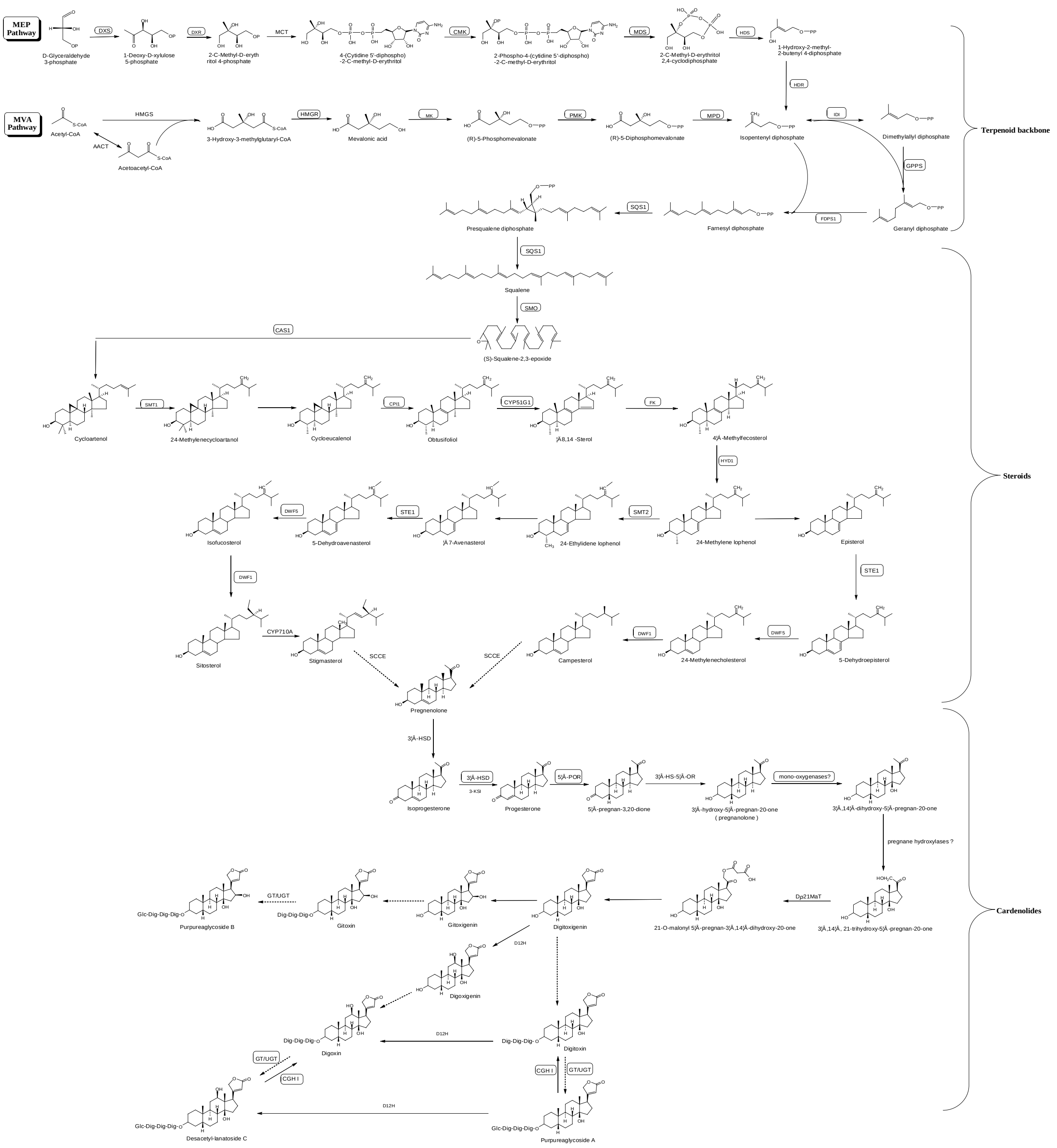

Additional file 8. The putative biosynthetic pathway of cardiac glycosides in *Digitalis Purpurea*.

The pathway roughly comprises terpenoid backbone biosynthesis, steroid biosynthesis and cardenolide biosynthesis three stages. The enzymes with corresponding unigenes are framed. Abbreviation: DXS,1-deoxy-D-xylulose-5-phosphate synthase; DXR,1-deoxy-D-xylulose-5-phosphate reductoisomerase; MCT, 2-C-methyl-D-erythritol 4-phosphate cytidylyltransferase; CMK, 4-diphosphocytidyl-2-C-methyl-D-erythritol kinase; MDS,2-C-methyl-D-erythritol 2,4-cyclodiphosphate synthase; HDS,4-hydroxy-3-methylbut-2-en-1-yl diphosphate synthase; HDR,4-hydroxy-3-methylbut-2-enyl diphosphate reductase; AACT,acetyl-CoA C-acetyltransferase; HMGS,hydroxymethylglutaryl-CoA synthase; HMGR,hydroxymethylglutaryl-CoA reductase; MK, mevalonate kinase; PMK, phosphomevalonate kinase; MPD, diphosphomevalonate decarboxylase;

IDI,isopentenyl-diphosphate delta-isomerase; GGPPS1,geranylgeranyl pyrophosphate synthase 1; FDPS1,farnesyl diphosphate synthase 1; SQS1,squalene synthase 1; SMO,squalene monooxygenase; CAS1,cycloartenol synthase 1; SMT1,sterol 24-C-methyltransferase; CPI1,cyclopropyl isomerase; CYP51G1,cytochrome P450, family 51, subfamily A (sterol 14-demethylase); FK, delta14-sterol reductase; HYD1， cholestenol delta-isomerase; SMT2，sterol methyltransferase 2; STE1， C-5 sterol desaturase; DWF5， sterol delta7 reductase ; DWF1，delta24-sterol reductase; SCCE， cholesterol monooxygenase (side-chain-cleaving); 3βHSD， 3-beta-hydroxysteroid dehydrogenase; 3-KSI，△5-3-ketosteroid isomerase; 5βPOR，progesterone 5β-reductase; 3βHS- P5βOR，3β-hydroxysteroid 5β-oxidoreductase; Dp21MaT，21-hydroxypregnane 21-O-malonyltransferase; D12H，digitoxin 12β-hydroxylase; CGHⅠ，cardenolide 16-O-glucohydrolase; GT，glycosyltransferase/ glucuronosyltransferase; UGT，UDP- glycosyltransferase/ glucuronosyltransferase.
