# Supplementary material for: Comprehensive transcriptome analysis reveals novel genes involved in cardiac glycoside biosynthesis and mlncRNAs associated with secondary metabolism and stress response in Digitalis purpurea
Source: BMC Genomics. 2012 Jan 10;13:15. doi: 10.1186/1471-2164-13-15 (PMC3269984; doi:10.1186/1471-2164-13-15)
Supplement: Additional file 9 — Primers used in qPCR. Complete set of the primers used in qPCR. [file 1471-2164-13-15-S9.PDF]

Additional file 9. Primers used in qPCR.

Gene Name	Primer sequence (5'-3')
ubiqutin	Forward: CTCTTCATTTGGTGTTGAGGCTTC<br>Reverse: TGCCTTCACATTATCGATGGTGTC
actin	Forward: TTCTTTGATCGGAATGGAATCTGC<br>Reverse: GTTGAACCACCACTGAGAACGATG
18s rRNA	Forward:TGACGGGTGACGGAGAATTAGG<br>Reverse:CCGTGTCAGGATTGGGTAATTTG
mlncR1	Forward: CATAGCGGAGATAACCGACATAG<br>Reverse: CAATCTTTCATCCACTCCACCTC
mlncR2	Forward: AAGAAAGCATGAAACGACAAAGCC<br>Reverse: TGCGAATATAATCAGGCTAGCACG
mlncR3	Forward: TCAACATCGTCCCAGATCTTTACGG<br>Reverse: GCGTGATCTCCGATTGTTTAT GGTC
mlncR4	Forward: TTATTAGACGAGGGCAGTGTTGTCA<br>Reverse: TGCACAATGCTCATCCTGACA G
mlncR5	Forward: CGCTAAGTGATTCCAGGACATCC<br>Reverse: GAAGAGGTGCTCACACATTAACGC
mlncR6	Forward: CACGCAATTTCTTGGATTTGCCGA<br>Reverse: GGTGTAGGTAGGCATCATTGAAAC
mlncR7	Forward: GATCGATTTCTTTCATTGGAGTTGG<br>Reverse: TGAGGCGATATCGAGTAACAGGG
mlncR8	Forward: TGCCTGGACATTCAGACTAAATTG<br>Reverse: CATCGTCGTCTTCTTCTTGAAACC
mlncR10	Forward: AACCCAACCCAACCTTGATCTG<br>Reverse: CCGAACCCGATTTCTCAATTACC
mlncR11	Forward: ACAGTCGCACGAAATTTGTAAGG<br>Reverse: CATAATTTAGCGCACTCTGCAAAC
mlncR12	Forward: GGAGTTGCGATAGCCAAGCATC<br>Reverse: TGGCGTGACAGTTCCCAGTG
mlncR13	Forward: CAAAGATTAGACGCCCACATACCG<br>Reverse: TCATGGTCGTATAGTGTTTGCGATG
mlncR14	Forward: TTCTGATGTTGAGCAATTCGATGG<br>Reverse: TGACACCATGAGCTCACCTGATATG
mlncR15	Forward: GTTTGAAAGGACACACCCATGGT<br>Reverse: AGGAACCCGTTGCTACTCGTG
mlncR16	Forward: CTTTGAGCTTGGGTGGTAAGGATG<br>Reverse: CGCAAGAAACAGCAACATGA AATC
mlncR17	Forward: TCATCAGTAAACGAGTGTGCAACC<br>Reverse: TTCTGAGTCCATATGAATCCA CACA
mlncR18	Forward: AAGGTCTAGCTTTGATTGCATCGG<br>Reverse: AGCAACACCATACGAACATAACCG
mlncR19	Forward: GGAAACTGAATAAAGTGGCCTCAAATC<br>Reverse: TCAAATGCTACTCTTGTTAGGCCTCA

mlncR20	Forward: GATCTCACTTCCGCCGCAAC<br>Reverse: TTTGATCAGCCACCATTCATGC
mlncR21	Forward: CCATCAAACTCAGACCAAACAAGC<br>Reverse: GCATTGAGATGTTTGCTTCCTT AGG
mlncR22	Forward: TTCAAGCTGGGTTGTTCAAGACTTC<br>Reverse: GACATGCATTGAAGATCCACCATC
mlncR23	Forward: CCAAACGAGCTGGGTCAGATCTC<br>Reverse: ACCCGGTCGTAACCCGATCA
mlncR24	Forward: CCTAGGATCCAGCTACGCCAAG<br>Reverse: CATGGGATTTGAACCTCCTACCTC
mlncR25	Forward: TGTAAATATCAACAGCAATCCAGGG<br>Reverse: ACTTCAGTTTGCTATCGTTCTCCG
mlncR26	Forward: CAACGAGTTTGGCGATTACTGCC<br>Reverse: TGGTGGAATTCGTACAAGGTATCGC
mlncR27	Forward: CCACAAATTTGAGATCCAACCAG<br>Reverse: GACGATAAATGTTGTGCCATGTG
mlncR28	Forward: CCAGTGACCTTACCTTCTCGAGC<br>Reverse: CAGCCTTAGGACTTCAGTGACCC
mlncR29	Forward: CGTTCGAATTTGCTTCACATAGAG<br>Reverse: TGTCAGGTTCCATATCTCACAGTG
mlncR30	Forward: GTTGAAACGACGACGACTGTGG<br>Reverse: TGGGCCATGTAGCACCACAC
mlncR31	Forward: ATTGCCACACAAGATATACGGGCC<br>Reverse: GGTTCATAGCCACATCCATTGCTTC
VHA-E	Forward: AGTCGAGAAGCATTCAGTTGCATC<br>Reverse: GAAGTTGCAGCTAGTGGAAGCG
SnRK	Forward: GGAAGATGAACTTTGAACCAAGGG<br>Reverse: CCAAGTCATTTGTCATCTGGAGCT
HDS	Forward: CGACCTCCAAGAGATTAGTGCAGA<br>Reverse: ACATAACCGAAATCAGCATCAGCC
SPS	Forward: AAAGTGACACGCGGAGAGGAAAG<br>Reverse: GATTGGCCAGGTACCATGATGAC
