# Supplementary material for: Comprehensive transcriptome analysis reveals novel genes involved in cardiac glycoside biosynthesis and mlncRNAs associated with secondary metabolism and stress response in Digitalis purpurea
Source: BMC Genomics. 2012 Jan 10;13:15. doi: 10.1186/1471-2164-13-15 (PMC3269984; doi:10.1186/1471-2164-13-15)
Supplement: Additional file 10 — Primers used for indentifying the transcriptional direction of mlncRNAs. Complete set of the primers used for indentifying the transcriptional direction of mlncRNAs. [file 1471-2164-13-15-S10.PDF]

Additional file 10. Primers used for indentifying the transcriptional direction of mlncRNAs.

Primer name	Sequence (5'-3')
GeneRacer™ Oligo dT Primer	GCTGTCAACGATACGCTACGTAACGGCATGACAGTG(T)24
GeneRacer™ 3′ Primer	CTGTCAACGATACGCTACGTAACG
GeneRacer 3' nested primer	GATACGCTACGTAACGGCATGAC
mlncR1 nesting primer	GGATTTGTTCATCATCTATTTTGGAGG
mlncR1 nested primer	CCTCCAATTGTGGAACCTCGTTG
mlncR6 nesting primer	GGGAGTGTAGCTGTGTCGGGTATG
mlncR6 nested primer	GGAAAGATAAACTTTCGAGGATGCC
mlncR8 nesting primer	AGGCAAAGGTCCCAACGACCCAAC
mlncR8 nested primer	GTTCCTTGAAGACCTGATTCAGAGG
mlncR31 nesting primer	GGTTCATAGCCACATCCATTGCTTC
mlncR31 nested primer	GCCCGTATATCTTGTGTGGCAATAC
